# The mitochondrial E3 ligase MAPL SUMOylates Drp1 to facilitate mitochondrial fission in intervertebral disc degeneration

**DOI:** 10.1038/s41413-025-00449-6

**Published:** 2025-08-12

**Authors:** Zhidi Lin, Xiao Lu, Guangyu Xu, Jian Song, Hongli Wang, Xinlei Xia, Feizhou Lu, Jianyuan Jiang, Wei Zhu, Zuochong Yu, Xiaosheng Ma, Fei Zou

**Affiliations:** 1 Department of Orthopedics, Huashan Hospital, Fudan University, Shanghai, China; 2 Department of Orthopedics, Jinshan Hospital, Fudan University, Shanghai, China

**Keywords:** Bone, Mitochondria

## Abstract

Intervertebral disc degeneration (IVDD) is the primary contributor to a range of spinal diseases. Dynamin-related protein 1 (Drp1)-mediated mitochondrial fission has recently been identified as a new cause of nucleus pulposus cell (NPC) death and IVDD, but the underlying mechanisms remain unclear. Although the effects of Drp1 phosphorylation in IVDD have been studied, it is currently unknown if small ubiquitin-like modifications (SUMOylation) of Drp1 regulate IVDD. This study aimed to investigate the functions and mechanisms of mitochondria-anchored protein ligase (MAPL), a mitochondrial SUMO E3 ligase, during IVDD progression. The expression of genes related to SUMOylation and mitochondrial dynamics in TNF-α-stimulated NPCs was analysed via RNA sequencing. The levels of total and mitochondrial SUMO1 conjugates were elevated with MAPL upregulation in TNF-α-treated NPCs. Additionally, mitochondrial fragmentation and dysfunction were induced by TNF-α stimulation. MAPL overexpression promoted mitochondrial SUMOylation and SUMO1 modification of Drp1, thereby facilitating the mitochondrial translocation of Drp1 and mitochondrial fission. MAPL-induced ROS accumulation and ΔΨm loss led to increased NPC apoptosis. Mutation of the SUMO-acceptor lysine residues of Drp1 hindered its SUMOylation and rescued the mitochondrial phenotypes caused by MAPL. SENP5 overexpression phenocopied MAPL silencing, negatively modulating the SUMO1 modification of Drp1 and mitochondrial fission in NPCs. In a rat IVDD model, forced expression of MAPL by using an adeno-associated virus (AAV) vector aggravated IVD tissue damage, whereas the knockdown of MAPL delayed IVDD progression. Our findings highlight the importance of SUMOylation in IVDD. The inhibition of MAPL-mediated Drp1 SUMOylation alleviates mitochondrial fission and limits IVDD development, providing a potential strategy for IVDD treatment.

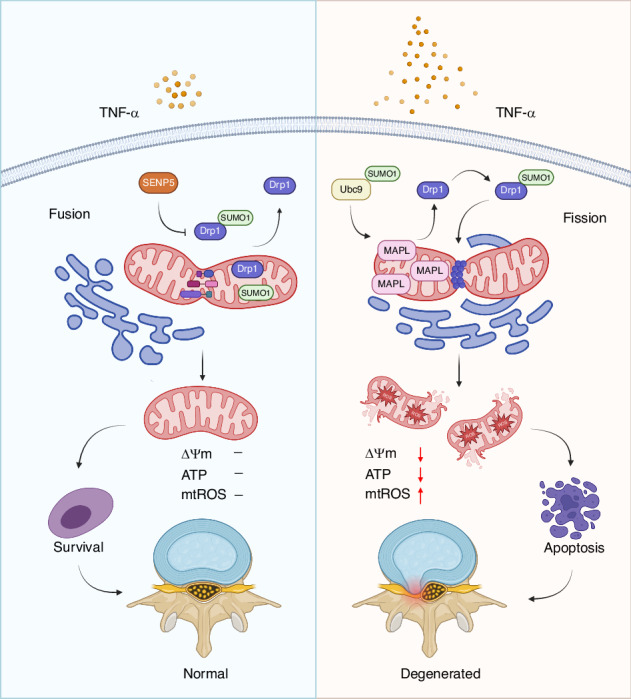

## Introduction

Intervertebral disc degeneration (IVDD) triggers a series of spinal diseases, such as discogenic low back pain, disc herniation, spinal stenosis, degenerative spondylolisthesis and scoliosis. To date, conservative treatment for IVDD is still limited to pain control and fails to halt the progression of IVD tissue damage.^1^ Discectomy and/or interbody fusion are widely performed and are considered gold-standard surgical treatments for various degenerative spinal diseases. Although these techniques achieve both neurological decompression and intervertebral stabilisation, adjacent segment degeneration/disease (ASD) is unavoidable and can even lead to reoperation.^2^ The identification of new therapeutic strategies is urgently needed. The intervertebral disc (IVD) is a fibrocartilaginous structure consisting of the central gelatinous nucleus pulposus (NP), surrounding annulus fibrosus (AF), and cartilaginous endplate (CEP) between adjacent vertebral bodies. Due to its avascular nature, the NP must receive nutrients and oxygen by diffusion through the CEP.^3^ Thus, the regenerative healing capacity of the NP is limited, and the NP is vulnerable to stress and ageing. Excessive cell death, especially NPC apoptosis, is one of the most crucial pathological characteristics of IVDD. Recently, from the perspective of epigenetics, our team reported that N6-methyladenosine (m6A)-methylated circGPATCH2L was endoribonucleolytically cleaved by a YTHDF2-RPL10-RNase P/MRP complex under physiological conditions.^4^ In degenerative NP tissues, circGPATCH2L, which has m6A hypomethylation, was upregulated. By acting as a protein decoy for tripartite motif containing 28 (TRIM28), circGPATCH2L inhibited the phosphorylation of TRIM28 and P53 degradation, contributing to DNA damage accumulation and NPC apoptosis. As the regulatory mechanisms underlying NPC death are intricate and remain elusive, the role of posttranslational modifications (PTMs) in IVDD is also of interest.

Mitochondrial homeostasis has attracted increasing attention in the field of IVDD.^5^ Mitochondria serve as energy factories and signal transduction platforms, modulating the intrinsic pathway of apoptosis.^6^ As highly dynamic organelles, mitochondria undergo rapid and continuous fusion and fission cycles, termed mitochondrial dynamics. Through the exchange of mitochondrial contents, fusion promotes complementation between damaged mitochondria in response to stress. Fission creates new mitochondria while also facilitating the selective removal of dysfunctional mitochondria.^7^ When mitochondrial dynamics fail to compensate for high levels of cell stress that lead to apoptosis, excessive fission is activated and contributes to mitochondrial fragmentation and dysfunction. Both fusion and fission are controlled by a series of highly conserved large guanosine triphosphatases (GTPases) in the dynamin family. Dynamin-related protein 1 (Drp1) is a key mediator of mitochondrial fission and predominantly resides in the cytosol. Once fission is initiated, Drp1 is recruited to the outer mitochondrial membrane (OMM) and self-assembles ring-like oligomers to constrict and cleave mitochondria in a GTP-dependent manner.^8^ Drp1 activity is regulated by posttranslational modifications (PTMs), such as phosphorylation, ubiquitination, SUMOylation, S-nitrosylation and O-GlcNAcylation.^9^ Structural and functional abnormalities in mitochondria have been observed in individuals with IVDD. Recently, our team designed engineered exosomes (CAP-Nrf2-Exos) displaying chondrocyte affinity peptide (CAP) on the surface and carrying the antioxidant transcription factor nuclear factor E2-related factor 2 (Nrf2).^10^ We found that CAP-Nrf2-Exos efficiently delivered Nrf2 into CEP cells, activated the endogenous antioxidant defence system and alleviated mitochondrial fragmentation and dysfunction by inhibiting Drp1 S616 phosphorylation and mitochondrial translocation. Our results shed more light on the importance of PTMs in modulating Drp1 functions. However, research into mitochondrial dynamics in IVDD is at an early stage.^11^ The roles of PTMs in IVDD, with the exception of phosphorylation, have rarely been studied.

Small ubiquitin-like modifiers (SUMOs) are ubiquitin-like proteins that conjugate to lysine residues in target proteins to regulate their stability, activity, subcellular localisation and molecular interactions. SUMOylation plays an essential regulatory role in multiple biological processes, including transcription, RNA processing, the DNA damage response, proteostasis and cell cycle progression.^12^ Like ubiquitination, SUMOylation is a three-enzyme cascade involving an activating enzyme (E1), a conjugating enzyme (E2) and an E3 ligase. Although five SUMO isoforms have been identified in mammalian cells, only the functions of SUMO1, SUMO2 and SUMO3 have been validated. SUMOylation is a dynamic and reversible process. Deconjugation or deSUMOylation is mediated by proteases, including SUMO-specific proteases (SENPs).^13^ An increasing number of studies have shown that SUMOylation is involved in the pathogenesis of osteoporosis,^14^ rheumatoid arthritis,^15^ and primary and metastatic bone tumours.^16^ However, very limited evidence is available concerning whether and how SUMOylation regulates IVDD. Owing to the avascular nature of IVDs, disc cells live in a physiologically hypoxic microenvironment. Wang et al.^17^ reported that SUMOylation-related proteins were expressed in IVD cells. When SENP1 expression was upregulated, both NP and AF cells tolerated hypoxia. Interestingly, distinct regulatory patterns of SUMOylation were observed in NP and AF cells in response to oxygen deficiency. SUMO1, SUMO2/3, SAE1/2 and UBC9 were differentially expressed between the NP and AF cells. Additionally, SENP1 was required for the stabilisation of HIF-1α, highlighting how SUMOylation is a highly specific and complex process.

The present study aimed to further explore the molecular mechanisms of PTMs in Drp1-mediated mitochondrial fission during IVDD. E3 ligases determine the specificity of SUMOylation, as they catalyse the final step of SUMOylation by transferring SUMOs from E2 to the target substrate. We, therefore, investigated whether SUMO E3 ligases regulate Drp1 SUMOylation in IVDD.

## Results

### SUMOylation and mitochondrial dynamics are associated with NPC apoptosis

**Fig. 1:**
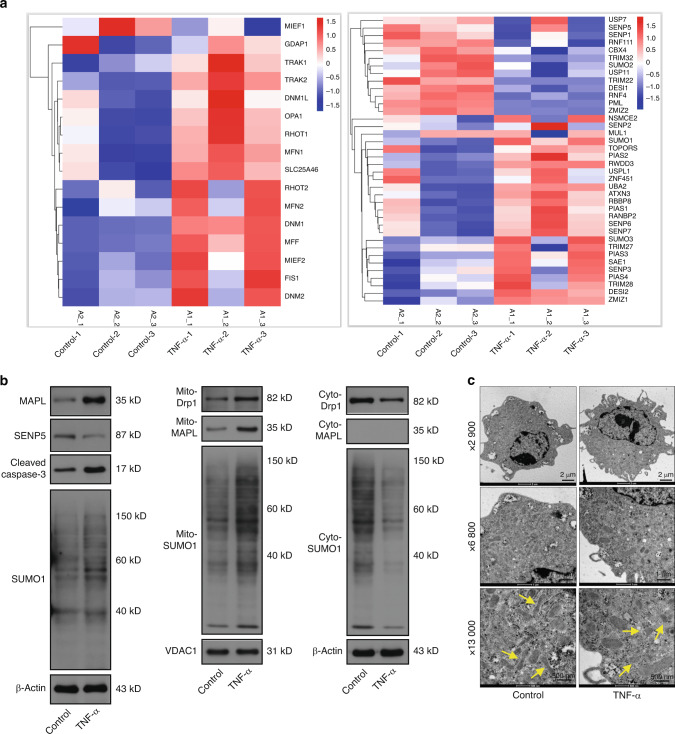
SUMOylation and mitochondrial dynamics are associated with NPC apoptosis. **a** RNA sequencing of NPCs treated with or without TNF-α to analyse the expression of SUMOylation-related genes and mitochondrial dynamics-related genes. **b** Western blot analysis of total MAPL, SENP5, cleaved caspase-3 and SUMO1 levels in TNF-α-treated NPCs. The mitochondrial and cytosolic levels of Drp1, MAPL and SUMO1 were also evaluated. **c** The morphological ultrastructural appearance of mitochondria in NPCs was observed via TEM. (*n* = 3; *P < 0.05, **P < 0.01, and ***P < 0.001)

The transcriptomic profiles of NPCs treated with or without TNF-α were analysed to determine whether SUMOylation participates in IVDD. The results of RNA sequencing suggested that the genes encoding SUMO enzymes, SUMO proteins and deSUMOylases were differentially expressed between the TNF-α group and the control group (Fig. 1a). The mRNA expression of fusion- and fission-related genes was also altered in NPCs under inflammatory conditions. Gene Ontology (GO) and Kyoto Encyclopedia of Genes and Genomes (KEGG) pathway enrichment analyses were conducted to characterise the biological functions of differentially expressed genes (DEGs) in the TNF-α-treated NPCs. Transcriptomic profiling revealed significant enrichment of GO terms associated with type-I interferon signalling, the inflammatory response, apoptotic processes, positive regulation of I-kappaB kinase/NF-kappaB signalling and TNF-α-mediated signalling (Fig. S1A, B). KEGG pathway analysis further identified dysregulation in TNF signalling, NOD-like receptor signalling, and the cytokine-cytokine receptor interaction pathways in the degenerated NPCs (Fig. S1C, D). These findings suggest that TNF-α transcriptionally activates genes involved in immune-inflammatory responses and apoptosis. However, neither the GO nor the KEGG analyses revealed significant enrichment of pathways related to SUMOylation or mitochondrial dynamics. Biological processes such as SUMOylation and mitochondrial dynamics might rely more heavily on protein-level regulation than on transcriptional changes.

Next, we investigated whether SUMOylation was correlated with mitochondrial dynamics. Given that MAPL was characterised as the first mitochondrial-anchored SUMO E3 ligase, we evaluated the protein level of MAPL by Western blotting. Interestingly, MAPL expression was increased in the TNF-α-induced NPCs, and SUMO1-mediated SUMOylation was increased (Fig. 1b and Fig. S1E–M). Notably, our RNA-seq data did not detect significant upregulation of MAPL mRNA following the TNF-α treatment (*P* = 0.338; FDR = 0.439). The increase in MAPL protein expression likely reflects TNF-α-induced post-transcriptional mechanisms, such as increased protein stability, reduced degradation or increased translational efficiency. Next, we found that Drp1, the central molecule involved in mitochondrial fission, was recruited from the cytosol to the mitochondria after the TNF-α treatment. The increased colocalization of Drp1 and MitoTracker Red was further validated by confocal microscopy (Fig. S2A). NPCs were stained with MitoTracker Green to observe the mitochondrial morphology. The results revealed that TNF-α shortened the branch lengths of the mitochondria, causing mitochondrial fragmentation (Fig. S2B). Small and fragmented mitochondria were also observed via transmission electron microscopy (TEM) (Fig. 1c). MitoSOX Red staining revealed increased accumulation of mitochondrial ROS, suggesting that activated fission led to mitochondrial dysfunction (Fig. S2C and Fig. S1N).

### MAPL SUMOylates Drp1 and facilitates its recruitment to mitochondria

**Fig. 2:**
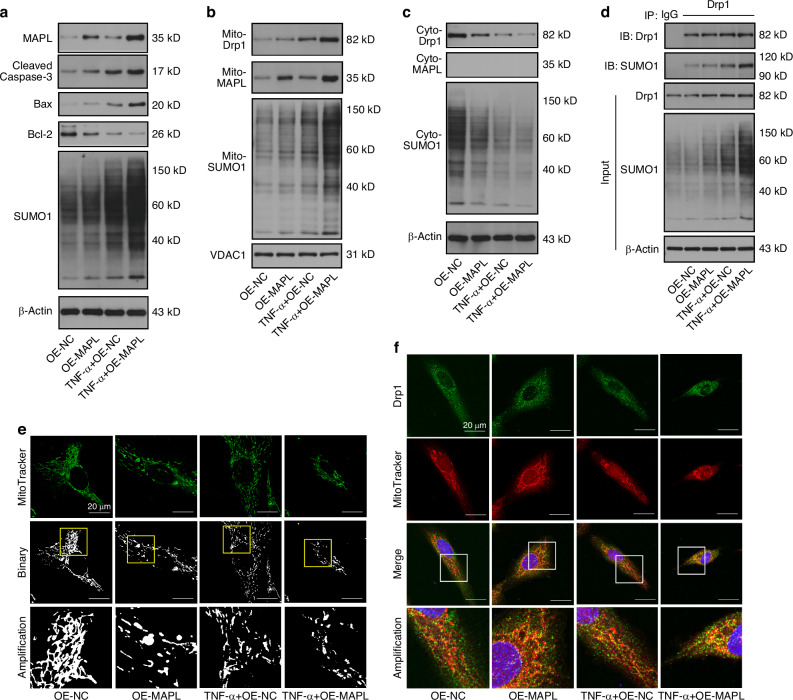
MAPL SUMOylates Drp1 and facilitates its recruitment to mitochondria. **a** The levels of total MAPL, cleaved caspase-3, Bax, Bcl-2 and SUMO1 in TNF-α-stimulated NPCs transfected with the MAPL overexpression plasmid were analysed by Western blotting. **b**, **c** Western blot analysis of the mitochondrial and cytosolic levels of Drp1, MAPL, and SUMO1 in NPCs after MAPL overexpression with or without TNF-α treatment. **d** SUMO1 modification of Drp1 was analysed by co-IP in MAPL-overexpressing NPCs under inflammatory conditions. **e** Mitochondrial fragmentation caused by MAPL overexpression was observed via MitoTracker Green staining. **f** The colocalization of Drp1 and MitoTracker Red was observed via confocal microscopy. (*n* = 3; *P < 0.05, **P < 0.01, and ***P < 0.001)

We performed gain-of-function experiments in NPCs with or without TNF-α treatment to explore the functions of MAPL-mediated SUMOylation in IVDD. The overexpression of MAPL increased the total level of cellular SUMO1 modification and the levels of proapoptotic proteins and decreased the level of the antiapoptotic protein Bcl-2 (Fig. 2a and Fig. S3A–E). After the mitochondrial and cytosolic fractions were separated from the NPCs, MAPL increased mitochondrial SUMOylation and facilitated the translocation of Drp1 from the cytosol to the mitochondria (Fig. 2b, c and Fig. S3F–J). Next, we investigated whether Drp1 was modified by SUMO1 in the NPCs. Coimmunoprecipitation (co-IP) was employed for this experiment. These data revealed that Drp1 interacted with SUMO1 and that MAPL promoted the SUMO1 modification of Drp1. The effects of MAPL were further enhanced under inflammatory stimulation (Fig. 2d and Fig. S3K). A series of assays were performed using mitochondrial dyes, immunofluorescence staining and flow cytometry to validate the alterations in mitochondrial dynamics and function. MitoTracker Green staining revealed that MAPL overexpression induced mitochondrial fragmentation. After the TNF-α treatment, MAPL further disrupted the mitochondrial network in the NPCs (Fig. 2e). Simultaneous staining of mitochondria and Drp1 indicated increased mitochondrial translocation of Drp1 when MAPL was overexpressed (Fig. 2f). JC-1 staining verified that MAPL exacerbated the TNF-α-induced loss of the mitochondrial membrane potential (ΔΨm) in the NPCs (Fig. S4B). MitoSOX Red (Figs. S4A and S3L) and DCFH-DA staining (Fig. S4C) revealed that MAPL increased mitochondrial and cellular ROS production. Annexin V-FITC/PI staining and flow cytometry revealed that MAPL increased the percentage of apoptotic NPCs (Fig. S4D). These results suggest that MAPL contributes to excessive fission, mitochondrial dysfunction and NPC death by promoting the recruitment of SUMOylated Drp1 to mitochondria.

### MAPL knockdown restricts Drp1 SUMOylation and mitochondrial fragmentation

**Fig. 3:**
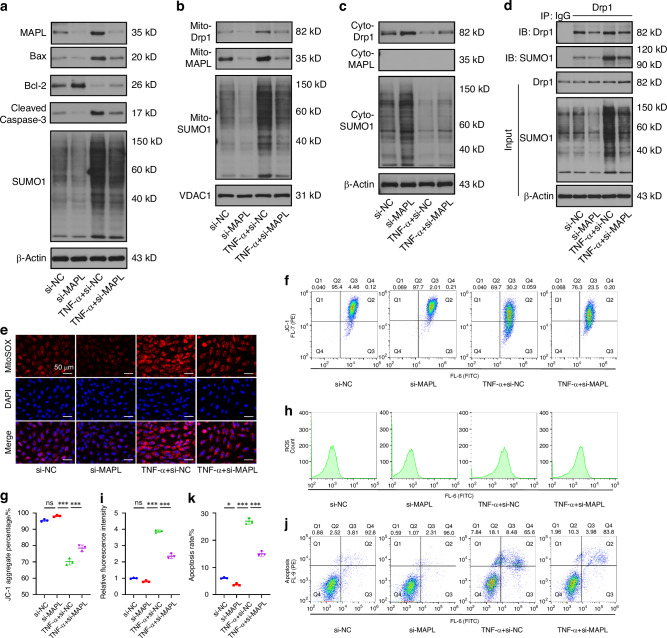
MAPL knockdown restricts Drp1 SUMOylation and mitochondrial fragmentation. **a** Western blots showing the levels of MAPL, Bax, Bcl-2, cleaved caspase-3 and SUMO1 in TNF-α-treated NPCs transfected with si-MAPL. **b**, **c** Western blots showing the levels of Drp1, MAPL and SUMO1 in the cytosolic fraction and mitochondrial compartment of NPCs after MAPL knockdown. **d** Co-IP of SUMOylated Drp1 in NPCs after treatment with si-MAPL. **e** Mitochondrial ROS production in NPCs transfected with si-MAPL. **f**, **g** JC-1 staining and flow cytometry were used to assess the ΔΨm in NPCs after MAPL knockdown. **h**, **i** DCFH-DA staining and flow cytometry were used to detect ROS accumulation in NPCs. **j**, **k** Flow cytometry with Annexin V-FITC/PI staining was used to evaluate the apoptosis rate of NPCs transfected with si-MAPL. (*n* = 3; *P < 0.05, **P < 0.01, and ***P < 0.001)

Genetic knockdown of MAPL was achieved using the siRNA to evaluate the necessity of MAPL in regulating the subcellular location of Drp1 and mitochondrial phenotypes. Western blot analysis revealed that the MAPL knockdown decreased the levels of cellular and mitochondrial SUMO1 conjugates and NPC apoptosis induced by TNF-α (Fig. 3a and Fig. S5A–E). A reduction in the amount of mitochondrial Drp1 translocated from the cytosol after MAPL silencing was observed, suggesting that MAPL-mediated mitochondrial SUMOylation was required for Drp1 redistribution (Fig. 3b, c and Fig. S5F–J). We subsequently performed co-IP assays and found that MAPL insufficiency reduced the level of Drp1-SUMO1 conjugates in the TNF-α-induced NPCs (Fig. 3d and Fig. S5K). Confocal microscopy revealed that the number of punctate or short rod-shaped mitochondria was decreased in the NPCs lacking MAPL following inflammatory stimulation (Fig. S6A). The subcellular distribution of Drp1 was greater in the cytosol than in the mitochondria after MAPL knockdown (Fig. S6B). Inhibition of MAPL also improved mitochondrial function, as indicated by the recovery of the ΔΨm and elimination of mitochondrial and cellular ROS (Fig. 3e–i and Fig. S5L). Moreover, MAPL downregulation decreased the rate of NPC apoptosis under the inflammatory conditions (Fig. 3j, k). Therefore, MAPL might be a novel therapeutic target for IVDD.

### Silencing Drp1 reverses MAPL-induced mitochondrial phenotypes and NPC death

We next silenced Drp1 using siRNAs to verify whether Drp1 acted downstream of MAPL. The MAPL-induced upregulation of cleaved caspase-3 and Bax and downregulation of Bcl-2 were reversed by Drp1 silencing (Fig. S7A and Fig. S8A–E). Both TEM and MitoTracker Green staining revealed that the Drp1 knockdown elongated mitochondrial branches and restored the mitochondrial networks in the MAPL-overexpressing NPCs (Fig. S7B, C). With respect to mitochondrial function, the inhibition of Drp1 expression suppressed MAPL-induced ROS accumulation and ΔΨm collapse (Fig. S7D–F and Fig. S8F). Consistently, silencing Drp1 rescued apoptotic NPC death mediated by MAPL (Fig. S7G). These results indicate the existence of the MAPL-Drp1 axis.

### Mutations of key lysine residues block Drp1 SUMOylation and rescue mitochondria-mediated NPC apoptosis

Drp1 consists of an N-terminal GTPase domain, a middle domain, an insert B domain, and a C-terminal GTPase-effector (GED) domain. Figueroa-Romero et al.^18^ performed a systematic mutational analysis to identify SUMO1 modification sites in Drp1. They reported that SUMOylation of Drp1 occurred at multiple nonconsensus sites within the B domain. Two lysine clusters in this region could function as SUMO-acceptor residues, including the N-terminal lysine residues (K532, K535, K558, and K568) and the C-terminal lysine residues (K594, K597, K606, and K608). We mutated these SUMO-acceptor lysine resides to arginines and monitored alterations in Drp1 distribution, mitochondrial phenotypes, and cell death in NPCs to precisely investigate the role of Drp1 SUMOylation in NPCs. The Drp1 plasmid with mutations at K532, K535, K558, and K568 is abbreviated as 4KR-N. The Drp1 plasmid with mutations at K594, K597, K606, and K608 is abbreviated as 4KR-C. The Drp1 plasmid with mutations at all eight lysine residues is termed 8KR.

**Fig. 4:**
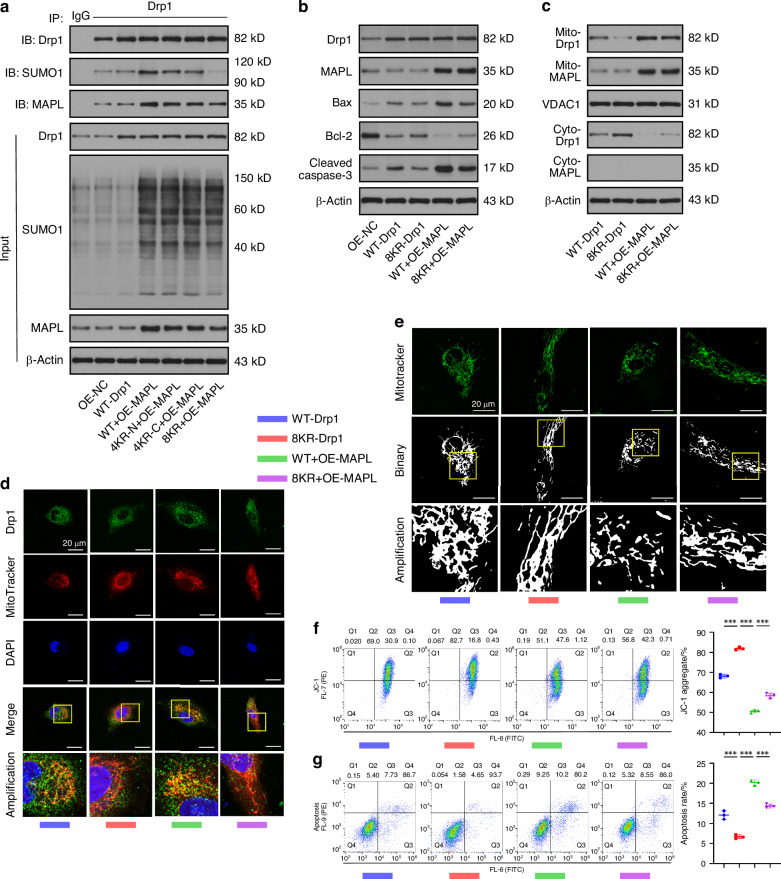
Mutation of key lysine residues blocks Drp1 SUMOylation and rescues mitochondria-mediated NPC apoptosis. **a** MAPL overexpression-mediated SUMOylation of Drp1 was analysed via co-IP in NPCs transfected with WT-Drp1 or the 4KR-N, 4KR-C and 8KR mutants. **b** Western blots showing the levels of total Drp1, MAPL, cleaved caspase-3, Bax, and Bcl-2 in NPCs transfected with the WT-Drp1 or 8KR plasmid after MAPL was overexpressed. **c** Western blot analysis of Drp1 and MAPL levels in the cytosolic fraction and mitochondrial compartment of NPCs after MAPL overexpression and lysine mutation of Drp1. **d** The translocation of Drp1 in NPCs was observed using MitoTracker Red staining and immunofluorescence staining for Drp1. **e** Changes in the morphology of mitochondria in MAPL-overexpressing NPCs transfected with WT-Drp1 or 8KR, as shown by MitoTracker Green staining. **f** JC-1 staining and flow cytometry were used to detect the ΔΨm in NPCs transfected with WT-Drp1 or 8KR after MAPL overexpression. **g** Flow cytometry with Annexin V-FITC/PI staining was used to detect the apoptotic rate of NPCs. (*n* = 3; *P < 0.05, **P < 0.01, and ***P < 0.001)

**Fig. 5:**
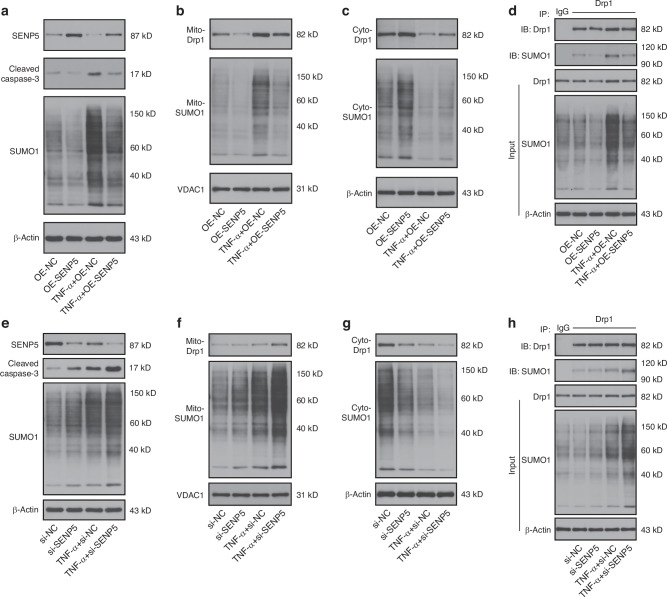
SENP5 negatively modulates Drp1 SUMOylation and mitochondrial fission. **a** Western blots showing the levels of total SENP5, cleaved caspase-3 and SUMO1 in NPCs after transfection with the SENP5 overexpression plasmid under inflammatory conditions. **b**, **c** Drp1 and SUMO1 expression in the cytosolic and mitochondrial fractions of the TNF-α-treated NPCs after SENP5 overexpression was measured via Western blotting. **d** Co-IP analysis of the SENP5-induced SUMO1 modification of Drp1 after TNF-α treatment. **e** Western blot analysis of total SENP5, cleaved caspase-3 and SUMO1 levels in NPCs after SENP5 knockdown and TNF-α treatment. **f**, **g** Mitochondrial and cytosolic levels of Drp1 and SUMO1 in TNF-α-treated NPCs transfected with si-SENP5 were assessed by Western blotting. **h** Drp1 SUMOylation after SENP5 silencing was analysed via co-IP. (*n* = 3; *P < 0.05, **P < 0.01, and ***P < 0.001)

We cotransfected WT-Drp1, 4KR-N, 4KR-C or 8KR with a plasmid overexpressing MAPL into NPCs and performed co-IP assays to detect the SUMO1 modification of Drp1. MAPL overexpression increased the level of SUMOylated Drp1 in the NPCs transfected with the WT-Drp1 plasmid. However, the SUMOylation of Drp1 was significantly decreased after the transfection of the 4KR-N or 4KR-C mutant. Notably, mutation of the eight key lysine residues almost completely eliminated the SUMO1 modification of exogenous Drp1 (Fig. 4a and Fig. S9A). Our further experiments revealed that the non-SUMOylated 8KR mutant blocked the MAPL-mediated translocation of Drp1 from the cytosol to the mitochondria. The protein levels of cleaved caspase-3 and Bax were decreased, and those of Bcl-2 were increased in the 8KR group, indicating that inhibition of Drp1 SUMOylation prevented MAPL-induced NPC apoptosis (Fig. 4b, c and Fig. S9B–I). In addition, confocal microscopy further revealed that the transfection of the 8KR mutant reversed the enhanced colocalization of Drp1 with mitochondria and excessive mitochondrial fragmentation caused by the overexpression of MAPL (Fig. 4d, e). Although MAPL promoted mitochondrial dysfunction, the non-SUMOylated mutant inhibited ΔΨm loss and ROS accumulation in the NPCs (Fig. 4f, Fig. S10A, B and Fig. S9J). The increase in the percentage of apoptotic NPCs caused by the overexpression of MAPL was reversed after the 8KR mutant was transfected (Fig. 5g). Therefore, MAPL regulated Drp1-dependent mitochondrial fission by modulating the SUMO1 modification of Drp1 in NPCs.

### SENP5 negatively modulates Drp1 SUMOylation and mitochondrial fission

Although SUMOylation is predominantly a nuclear protein modification and most SUMO-regulated cellular processes occur in the nucleus, there is increasing recognition that the SUMOylation machinery and substrates are present and active in extranuclear compartments.^19^ Previous studies have demonstrated the mitochondrial and cytosolic distribution of SENP5, which is essential for its interaction with Drp1.^20^ Based on this evidence, we investigated the deSUMOylase SENP5 to determine whether SENP5 and MAPL exert opposing effects on the SUMO1-mediated modification of Drp1. This approach aims to further elucidate the regulatory mechanisms underlying Drp1 SUMOylation. Gain-of-function and loss-of-function experiments were performed in an in vitro TNF-α-induced IVDD model. Western blot analysis revealed that SENP5 overexpression reduced the levels of SUMO1 conjugates and cleaved caspase-3 in NPCs after the TNF-α treatment (Fig. 5a and Fig. S11A‒C). The TNF-α-induced increases in mitochondrial SUMOylation and Drp1 translocation were reversed by SENP5 overexpression (Fig. 5b, c and Fig. S11D–G). Co-IP analysis revealed that SENP5 reduced the SUMO1 modification of Drp1 in the TNF-α-induced NPCs (Fig. 5d and Fig. S11H). We then used siRNAs to knock down the expression of SENP5 and found that SENP5 silencing increased cellular and mitochondrial SUMOylation and enhanced the recruitment of Drp1 to mitochondria (Fig. 5e–g and Fig. S11I-O). Similar to MAPL overexpression, SENP5 knockdown promoted the SUMO1 modification of Drp1 (Fig. 5h and Fig. S11P). MitoTracker Green staining revealed that SENP5 restored the mitochondrial network, whereas SENP5 knockdown contributed to mitochondrial fission (Fig. S12A, B). These results demonstrate that SENP5 inhibits Drp1 SUMOylation and mitochondrial fragmentation.

Furthermore, SENP5 knockdown increased the intracellular and mitochondrial ROS levels under the basal conditions relative to those in the si-NC group. Under inflammatory stimulation, SENP5 depletion further increased ROS accumulation compared with that in the TNF-α+si-NC group, demonstrating that SENP5 deficiency exacerbates ROS generation during pathological stress (Fig. S13A, B and Fig. S13E, F). JC-1 staining revealed that SENP5 depletion induced ΔΨm loss under the basal and TNF-α-treated conditions, suggesting that the SENP5 knockdown triggered mitochondrial dysfunction (Fig. S13C, D). Flow cytometry analysis with Annexin V/PI staining revealed that SENP5 silencing not only increased baseline apoptotic activity but also increased TNF-α-induced cell death (Fig. S13G, H). Collectively, these findings establish SENP5 as a key protective regulator of ROS accumulation and apoptosis in NPCs, with particularly pronounced effects under inflammatory challenge. Mechanistically, these findings align with our proposed model in which SENP5-mediated deSUMOylation antagonises MAPL-dependent SUMOylation of Drp1, thereby suppressing excessive mitochondrial fission.

### The SUMOylation and mitochondrial translocation of Drp1 are coregulated by MAPL and SENP5

**Fig. 6:**
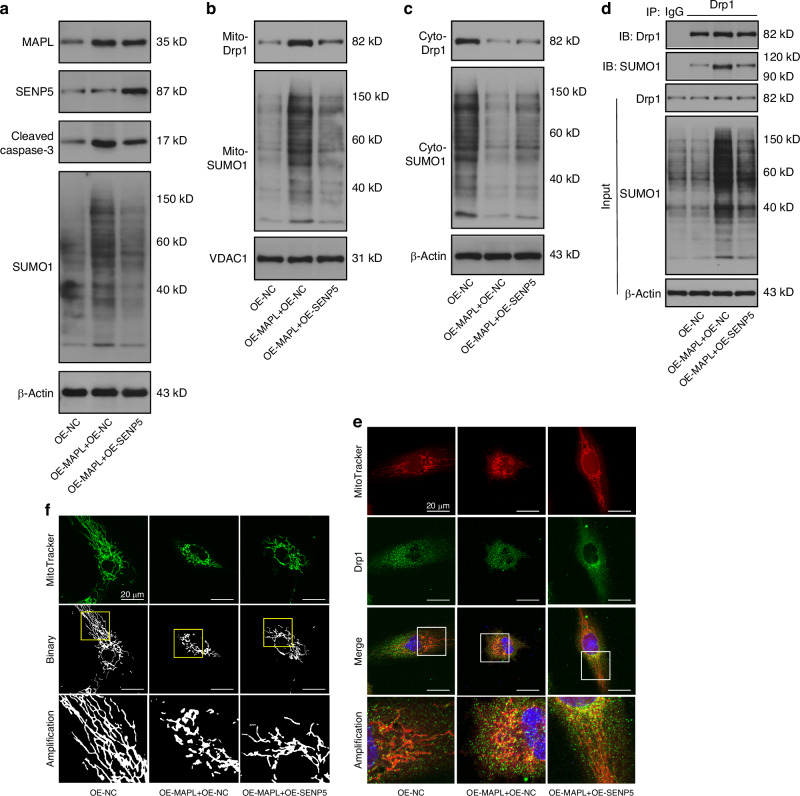
SUMOylation and mitochondrial translocation of Drp1 are coregulated by MAPL and SENP5. **a** Western blots showing the levels of total MAPL, SENP5, cleaved caspase-3, and SUMO1 in NPCs transfected with OE-MAPL or OE-SENP5 plasmids. **b**, **c** Mitochondrial and cytosolic levels of Drp1 and SUMO1 in MAPL-overexpressing and SENP5-overexpressing NPCs were assessed using Western blotting. **d** Co-IP of SUMOylated Drp1 in NPCs overexpressing MAPL and SENP. **e** MitoTracker Red staining and immunofluorescence staining for Drp1 in NPCs after MAPL or SENP5 overexpression. **f** Changes in the morphology of mitochondria in NPCs transfected with OE-MAPL or OE-SENP5, as shown by MitoTracker Green staining. (*n* = 3; *P < 0.05, **P < 0.01, and ***P < 0.001)

MAPL and SENP5 were overexpressed simultaneously in NPCs to confirm that Drp1 SUMOylation is coregulated by MAPL and SENP5 and that Drp1 redistribution is a common downstream event of these two proteins. As shown by Western blot analysis, MAPL increased the levels of cellular and mitochondrial SUMO1 modifications, but SENP5 suppressed these effects. SENP5 also reversed MAPL-mediated Drp1 SUMOylation and mitochondrial translocation (Fig. 6a–e and Fig. S14A–I). Confocal microscopy revealed that the number of fragmented mitochondria observed after MAPL overexpression was reduced by SENP5 overexpression (Fig. 6f). Thus, MAPL and SENP5 constitute a pair of modulators that manipulate Drp1 SUMOylation.

### Inhibition of MAPL delayed IVDD in a rat model

**Fig. 7:**
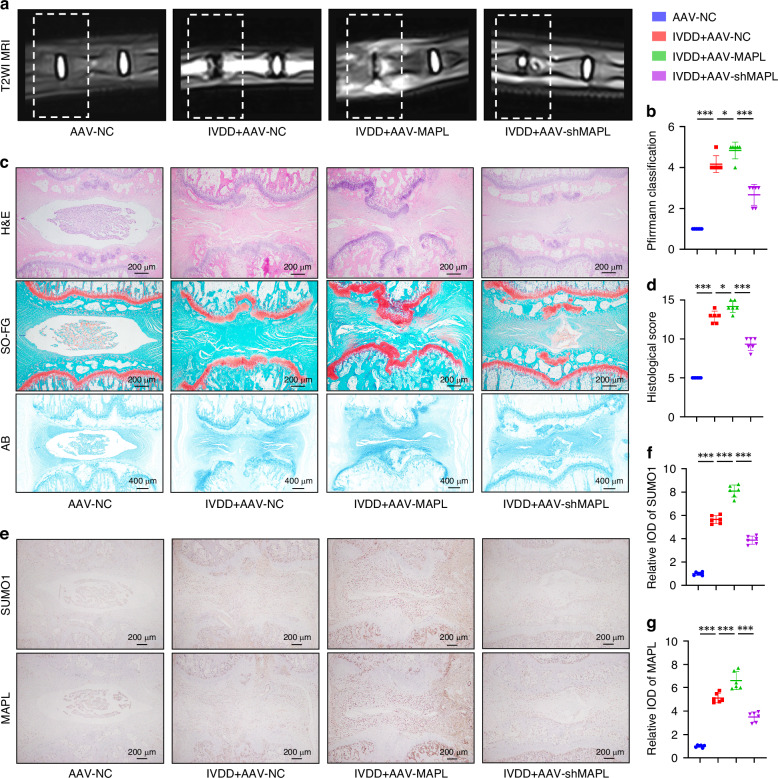
Inhibition of MAPL delays IVDD in a rat model. **a**, **b** The coccygeal vertebrae of the rats were examined by MRI, and the degree of IVDD was evaluated by using the Pfirrmann classification. **c** H&E staining, Safranin O‒Fast Green staining and Alcian blue staining of rat IVD samples. **d** Evaluation of IVDD by histological scoring. **e**–**g** Immunohistochemical analysis of SUMO1 and MAPL levels. (*n* = 6; *P < 0.05, **P < 0.01, and ***P < 0.001)

We established an IVDD rat model by a needle puncture. Rats were divided into the AAV-NC group, IVDD + AAV-NC group, IVDD + AAV-MAPL group or IVDD + AAV-shMAPL group. After four weeks, MRI results revealed a significant reduction in T2-weighted signals within the IVDs of the rats subjected to needle puncture. Furthermore, MAPL overexpression induced disc space collapse and a loss of distinction between the NP and AF. In contrast, MAPL knockdown facilitated the repair of degenerated IVDs (Fig. 7a, b). Histological analysis using H&E, Safranin O-Fast Green and Alcian blue staining showed that the needle puncture treatment triggered IVDD, as evidenced by tissue damage in the NP, a loss of NPCs and disruption of the NP/AF border. Notably, MAPL overexpression exacerbated IVD structural disorganisation, whereas MAPL knockdown attenuated IVDD progression (Fig. 7c, d). Immunohistochemistry revealed elevated expression levels of MAPL, SUMO1 and cleaved caspase-3 in the degenerated IVD tissues, with the highest levels observed in the IVDD + AAV-MAPL group. However, MAPL silencing reduced the expression of these proteins, suggesting that MAPL-mediated SUMOylation contributes to IVD cell death in vivo (Fig. 7e–g and Fig. S15A, B). Collectively, these findings confirmed that targeting MAPL depletion could represent a promising novel therapeutic strategy for IVDD treatment.

## Discussion

Accumulating evidence has shown that excessive mitochondrial fission leads to the progression of IVDD. However, our previous literature review indicated that very few studies have focused on posttranslational modifications of dynamics-related proteins, interorganelle contacts or the crosstalk between fission and mitophagy in the field of IVDD.^11^ Recently, Yang and collaborators have conducted two elegant and interesting studies. In a study of the interaction between mitochondrial dynamics and mitophagy, they found that NLRX1, a mitochondrial NOD-like receptor, can maintain selective mitochondrial fission and mitophagy to restore adaptive mitochondrial morphology in senescent NPCs.^21^ Downregulation of NLRX1 in IVDD activated the compensatory PINK1–Parkin pathway to induce overactivated mitophagy and aggravated mitochondrial dysfunction. Mechanistically, NLRX1 interacts with the Zn^2+^ transporter SCL397A and forms an NLRX1-SLC39A7 complex to modulate mitochondrial Zn^2+^ trafficking, orchestrating mitochondrial dynamics and mitophagy. With respect to the crosstalk between mitochondria and the endoplasmic reticulum (ER), they reported that mitochondria-associated ER membrane (MAM) disruption and mitochondrial Zn^2+^ overload in senescent NPCs subjected to oxidative stress.^22^ The formation of the NLRX1-SLC39A7 complex was inhibited by the loss of MAM tethering. The OMM protein SYNJ2BP facilitated both MAM and NLRX1-SLC39A7 complex formation, thus improving mitochondrial Zn^2+^ homeostasis and ameliorating IVDD. SUMOylation is an essential PTM, but most existing studies on mitochondrial dynamics and IVDD have concentrated only on Drp1 phosphorylation. We showed that MAPL, a SUMO E3 ligase present in mitochondria, can promote the SUMO1 modification of Drp1, facilitate its mitochondrial translocation and induce detrimental fission, leading to mitochondrial dysfunction, NPC apoptosis, and IVDD.

Mitochondrial quality control mechanisms are involved in multiple degenerative joint diseases, such as IVDD and osteoarthritis (OA). IVDD and OA have considerable pathophysiological overlap, involving mainly aberrant mechanical loading, inflammation, oxidative stress, senescence, cell death, and extracellular matrix (ECM) degradation. Like chondrocytes, NPCs govern the homeostasis of the IVD by controlling ECM metabolism. The dysfunction and death of these two types of cells accelerate IVDD and OA progression.^23^ Although the role of SUMOylation in IVDD remains largely unknown, some progress has been made in understanding the relationship between chondrocytes and SUMOylation. Wang et al.^24^ observed abnormal adipogenesis of chondrocytes and decreased growth differentiation factor 11 (GDF11) expression in subjects with OA. GDF11 is an inhibitor of the differentiation of bone marrow mesenchymal stem cells into adipocytes. The downregulation of GDF11 promoted adipogenesis in degenerated cartilage and chondrocytes. Rather than affecting the proliferator-activated receptor γ (PPARγ) expression levels, GDF11 increased the SUMO1 modification of PPARγ and inhibited its effects on abnormal adipogenesis in subjects with OA. Gong et al.^25^ found that circRREB1 and the lipogenic enzyme fatty acid synthase (FASN) are highly expressed in senescent chondrocytes. CircRREB1 enhances the interaction between FASN and the SUMO E2 enzyme UBC9, as well as the interaction between FASN and the SUMO E3 ligase RanBP3. Thus, circRREB1 increases the SUMO2/3 modification of FASN and impedes its proteasomal degradation. RanBP2-mediated SUMOylation and stabilisation of FASN maintains its ability to aggravate senescence and OA phenotypes. Osteochondroprogenitors (OCPs) are the common mesenchymal precursors of chondrocytes and osteoblasts. Yang et al.^26^ reported that the loss of the deSUMOylase SENP6 caused impaired skeletal growth, kyphosis and premature ageing in mice. Knockout of SENP6 increased OCP and chondrocyte senescence and apoptosis. Mechanistically, SENP6 directly interacted with TRIM28 and deconjugated the SUMO3 modification at K554, K804, and K779. SENP6-mediated deSUMOylation and stabilisation of TRIM28 suppressed the p53 signalling pathway. Hence, targeting the SUMOylation/deSUMOylation machinery may be a novel strategy for treating cartilage-related disorders and degenerative joint diseases.

How SUMOylation modulates mitochondrial dynamics is an attractive topic. McBride’s group has achieved groundbreaking success in the study of mitochondria-derived vesicles (MDVs), mitochondrial SUMOylation and mitochondrial dynamics. They reported that the overexpression of MAPL resulted in a fragmented mitochondrial phenotype in 2008.^27^ MAPL participated in the regulation of Drp1-mediated mitochondrial fission in a RING finger-dependent manner. Selective packaging of MAPL facilitated the transport of MDVs to peroxisomes. They further explored MAPL as the first mitochondrial SUMO E3 ligase, providing new insights into the importance of nonnuclear SUMOylation.^28^ In HeLa cells, MAPL increased SUMO1 conjugation to Drp1 and stimulated mitochondrial fission. MAPL-induced mitochondrial SUMOylation is required for programmed cell death.^29^ SUMOylated Drp1 stabilised ER/mitochondrial contact sites, mediating calcium flux, cristae remodelling and cytochrome c release. In addition, the authors reported that the SUMO protease SENP5 negatively regulated mitochondrial fission by deSUMOylating SUMO1 from Drp1. In COS-7 cells, the silencing of SENP5 resulted in mitochondrial fragmentation and increased ROS production.^30^ Our data confirmed that mitochondrial SUMOylation was increased and that mitochondrial fission was activated in TNF-α-induced NPCs. Gain-of-function and loss-of-function assays demonstrated that MAPL promoted the SUMO1 modification of Drp1 during IVDD. SUMOylated Drp1 is translocated from the cytosol to the mitochondria, where it triggers excessive mitochondrial fission. Drp1 knockdown reversed the phenotypes caused by MAPL overexpression in the NPCs, indicating that Drp1 is downstream of MAPL. Overexpression of the deSUMOylase SENP5 or the mutation of key lysine residues prevented Drp1 SUMOylation and mitochondrial translocation, rescuing mitochondrial fragmentation, ΔΨm loss, ROS accumulation and NPC apoptosis. In an IVDD rat model, MAPL knockdown ameliorated NP cell loss and IVD tissue damage. Indeed, SUMOylation is involved in the regulation of IVDD. In addition to phosphorylation, Drp1 activity is also modulated by SUMO1 modification in NPCs. Inhibiting MAPL-mediated Drp1 SUMOylation may provide new therapeutic strategies for IVDD treatment.

In summary, we constructed a bridge between SUMOylation-mediated mitochondrial fission and IVDD, emphasising the importance of Drp1 PTMs in the pathological process of NPC death. However, our study has several limitations worth mentioning. First, whether SUMOylation itself directly promotes the mitochondrial translocation of Drp1 or only enables Drp1 to remain in the mitochondria after being manipulated by phosphorylation remains unverified. Second, further studies are needed to describe the interactions among different PTMs, which may create an intricate regulatory network influencing the biological functions of Drp1. Third, although Drp1 is the central mediator of mitochondrial fission, more attention should be given to other mitochondrial dynamics-related proteins, especially in the field of IVDD. Moreover, physiological fission is indispensable for cellular homeostasis, and the overinhibition of mitochondrial fission can be detrimental. The ideal therapeutic goal is to restore the balance between fission and fusion. Consequently, multitarget drugs or combinations of drugs may prevent the occurrence of potential side effects attributed to the disruption of mitochondrial dynamics.

## Materials and methods

### Ethics statement

This study was approved by the Ethics Committee of Huashan Hospital, Fudan University (KY2024-1020). All the experiments involving animals were approved by the Animal Care and Use Committee of Fudan University (No. 202410048S).

### Human NPC isolation and culture

Primary NPCs were isolated from IVD tissues obtained from 26 patients diagnosed with Hirayama disease who underwent anterior cervical discectomy and fusion. Magnetic resonance imaging (MRI) was used to evaluate the degree of IVDD according to the Pfirrmann grading system. To guarantee that only healthy, non-degenerated IVD tissues were included, the Pfirrmann grade I classification was strictly adhered to during sample selection. The NP tissues were cut into pieces and digested with 0.2% collagenase II (Thermo Fisher Scientific, USA) for 8 h at 37 °C. The released cells were collected by centrifugation for 5 min at 500 × *g*. The NPCs were then resuspended and cultured in Dulbecco’s modified Eagle’s medium (DMEM) supplemented with 15% foetal bovine serum (FBS) and maintained in an incubator at 37 °C with 5% CO_2_. The culture medium was changed every three days. NPCs at the second passage were used for further experiments. To mimic NP degeneration in vitro the NPCs were treated with 10 ng/mL TNF-α for 24 h.

### RNA sequencing

Total RNA was extracted from the control and TNF-α groups using TRIzol reagent (Invitrogen, USA). After being qualitatively checked, the mRNA was enriched with oligo (dT)-coated magnetic beads and fragmented for reverse transcription into cDNA with random primers. Then, the cDNA fragments were purified, terminally modified and ligated to sequencing adapters. The ligation products were size selected by gel electrophoresis, amplified by PCR and used for cDNA library preparation. Libraries were sequenced using the HiSeq X Ten system (Illumina, USA). The raw sequencing data were processed and mapped to the human reference genome using STAR software. Genes that were differentially expressed (a P value cut-off of 0.05 and a fold-change cut-off of 2) between the two groups were analysed using the DESeq2 package.

### RNA interference and plasmid transfection

The plasmids pcDNA3.1-MAPL, pcDNA3.1-SENP5, WT-Drp1, 4KR-N Drp1 (K532R, K535R, K558R, K568R), 4KR-C Drp1 (K594R, K597R, K606R, K608R) and 8KR Drp1 (K532R, K535R, K558R, K568R, K594R, K597R, K606R, K608R) were constructed by GeneChem (Shanghai, China). A set of siRNAs was designed and synthesised by GeneChem (Shanghai, China). The sequences used were as follows: Homo si-MAPL: 5′-AGGAGCTGTGCGGTCTGTTAAAGAA-3′; si-NC (MAPL): 5′-GATACTTGACTAGGAAACCCACACA-3′; Homo si-Drp1: 5′- GCTGTTTCTAAAGTTTCCCAGTATA-3′; si-NC (Drp1): 5′- CCATGGTCTAATTGTCACATCATGT-3′; Homo si-SENP5: 5′- GCATCAGGTTGTAGTTGCATCTTTA-3′; and si-NC (SENP5): 5′- CAGAGAACTTACAGTGTACAACATA-3′. The NPCs were transfected with plasmids or siRNAs using Lipofectamine 3000 (Invitrogen, USA) according to the manufacturer’s instructions.

### Western blot analysis

NPCs were lysed in RIPA buffer (AspenTech, China) on ice, and the protein concentration was quantified using a BCA protein assay kit (AspenTech, China). The isolated proteins were separated by sodium dodecyl sulfate‒polyacrylamide gel electrophoresis (SDS‒PAGE) (Millipore, USA). The proteins were subsequently transferred onto polyvinylidene fluoride (PVDF) membranes (Millipore, USA), which were subsequently blocked with 5% milk. Primary antibodies were added, and the membranes were incubated overnight at 4 °C. After incubation with horseradish peroxidase-conjugated secondary antibodies, the membranes were visualised with chemiluminescence reagents (AspenTech, China) and an imaging system. The primary antibodies used were as follows: anti-MAPL (16133-1-AP, 1:1 000, Proteintech), anti-SENP5 (ab58420, 1:500, Abcam), anti-SUMO1 (67559-1-Ig, 1:1 000, Proteintech), anti-cleaved caspase-3 (AF7022, 1:500, Affinity Biosciences), anti-Bax (#2772, 1:1 000, Cell Signalling Technology), anti-Bcl-2 (1:1 000, ab196495, Abcam), anti-Drp1 (#8570, 1:1 000 (IB) or 1:100 (IP), Cell Signalling Technology), anti-VDAC1 (ab15895, 1:3 000, Abcam) and anti-β-actin (TDY051, 1:10 000, TDY Biotech).

### Coimmunoprecipitation

After the indicated treatments, the NPCs were washed twice with precooled PBS and lysed with NP-40 lysis buffer (AspenTech, China) supplemented with a 1% protease inhibitor cocktail (AspenTech, China) and 1% PMSF (AspenTech, China) on ice for 30 min. After centrifugation at 12 000 r/min for 15 min at 4 °C, the supernatant was collected, and the protein concentration was determined with a BCA protein assay kit (AspenTech, China). The same amount of protein from each group was added to 10 μL of anti-Drp1 antibody (Cell Signalling Technology, USA) and incubated with Protein A/G magnetic beads (Bio-Rad, USA) at 4 °C overnight. The immunoprecipitate was eluted with SDS‒PAGE loading buffer at 95 °C for 10 min and analysed by Western blot analysis.

### Immunofluorescence staining

NPCs were fixed with 4% paraformaldehyde for 20 min, permeabilised with 0.5% Triton X-100 for 15 min and blocked with 5% bovine serum albumin for 30 min. The samples were then incubated with the primary anti-Drp1 antibody (1:100) at 4 °C overnight. After three washes with PBS, the samples were incubated with the corresponding secondary antibodies for 40 min in the dark. The nuclei were stained with DAPI (C1006; Beyotime, China) for 20 min. Finally, representative images were acquired using a fluorescence microscope (Olympus, Japan) and fluorescence confocal microscope (Zeiss, Germany).

### Apoptosis assay

NPC apoptosis was evaluated by flow cytometry and Annexin V-FITC/PI staining. An apoptosis analysis kit (BD Biosciences, USA) was used to stain the NPCs. Briefly, the NPCs were incubated with 5 μL of Annexin V-FITC and 5 μL of PI for 15 min in the dark after being resuspended. The rate of apoptosis was analysed using a flow cytometer (Beckman Coulter, USA).

### Mitochondrial membrane potential (ΔΨm) assessment

The ΔΨm was measured using a JC-1 Assay Kit (Beyotime, China). NPCs were incubated with JC-1 staining working solution for 20 min at 37 °C. The samples were subsequently washed twice with JC-1 buffer solution. After being resuspended, the stained NPCs were analysed via flow cytometry. The ratio of JC-1 aggregates to monomers was estimated as an indicator of alterations in the mitochondrial membrane potential.

### Measurement of mitochondrial reactive oxygen species (mtROS) and cellular ROS levels

Cellular ROS and mtROS levels were examined with a ROS assay kit (Beyotime, China) and MitoSOX Red (Thermo Fisher Scientific, USA), respectively. NPCs were stained with MitoSOX Red for 30 min or DCFH-DA for 20 min at 37 °C in the dark. The cells were then washed three times with PBS. The NPCs were observed under a fluorescence microscope (Olympus, Japan) to detect mtROS. Flow cytometry (Beckman Coulter, USA) was used to measure the cellular ROS level.

### Mitotracker staining

MitoTracker Green (Beyotime, China) and MitoTracker Red CMXRos (Beyotime, China) were used to stain mitochondria according to the manufacturer’s instructions. Briefly, after the media were discarded, the NPCs were incubated with MitoTracker Green working solution or MitoTracker Red working solution at 37 °C for 30 min. A fluorescence confocal microscope (Zeiss, Germany) was used to capture representative images.

### Transmission electron microscopy

NPCs were fixed with 2.5% glutaraldehyde for 1 h at 4 °C. The cells were then fixed with 1% osmium tetroxide for 2 h at 37 °C. The samples were subsequently dehydrated and embedded in Epon 812 (Shell Chemicals, USA). Ultrathin sections were cut at thicknesses of 60–70 nm with an ultramicrotome (Leica, Germany) and stained with uranyl acetate and lead citrate. Images were obtained with a transmission electron microscope (Hitachi, Japan).

### Animal model and intradiscal injection

We used adeno-associated virus (AAV)-mediated overexpression and knockdown of MAPL in animal experiments. The AAVs were purchased from GeneChem (Shanghai, China). Twenty-four Sprague‒Dawley rats (male, 8 weeks old) were randomly assigned to four groups (*n* = 6): the AAV-NC group, IVDD + AAV-NC group, IVDD + AAV-MAPL group, and IVDD + AAV-shMAPL group. The rats were anaesthetised via an intraperitoneal injection of 2% pentobarbital (40 mg/kg). The experimental level IVD at Co7/8 was located by digital palpation of the coccygeal vertebrae. A 20-gauge needle was used for vertical puncture at the centre of the disc through the AF and into the NP. The depth of penetration was approximately 5 mm. The needle was rotated 360° and kept in the disc for 30 s. A total of 2 µL of solution containing the AAV vector, AAV-MAPL or AAV-sh-MAPL (1 × 10^9^ pfu/mL) was intradiscally injected into the centre of the NP region with a microlitre syringe once a week for four weeks to investigate the biological functions of MAPL. Finally, the rats were returned to their cages with unrestricted activity and sacrificed one month after the surgery.

### MRI

Radiographic evaluation of the rat coccygeal vertebrae was performed with MRI (BioSpec 70/30 USR, Bruker, Germany) to assess signal intensity alterations and structural abnormalities in sagittal T2-weighted sequences. The severity of IVDD was classified according to the Pfirrmann grading system, a well-established clinical classification method.^31^

### Histological staining and immunohistochemistry

The samples were collected, fixed, decalcified, dehydrated, embedded in paraffin, and cut into sections. H&E, Safranin O-fast green and Alcian blue staining were performed to analyse the degree of IVDD. Immunohistochemistry was performed to analyse the expression levels of MAPL (16133-1-AP, 1:200, Proteintech), SUMO1 (67559-1-Ig, 1:500, Proteintech), and cleaved caspase-3 (AF7022, 1:200, Affinity Biosciences). The integrated optical density (IOD) of the immunohistochemical images was quantified using ImageJ software.

### Statistical analysis

The data in this study are presented as the means ± standard deviations (SDs). Three biological replicates were performed for the in vitro experiments, and six biological replicates were performed for the in vivo experiments. The statistical analyses were performed using SPSS 21.0 and GraphPad Prism 9.0 software. The data were analysed via Student’s *t* test for comparisons between two groups and one-way analysis of variance followed by Tukey’s test for comparisons among multiple groups. A value of *P* < 0.05 indicated statistical significance (*P < 0.05, **P < 0.01 or ***P < 0.001).

## Supplementary information

Supplemental Materials

Original Western Blots

## Data Availability

The data are available upon reasonable request.
