# Supplementary material for: The mitochondrial E3 ligase MAPL SUMOylates Drp1 to facilitate mitochondrial fission in intervertebral disc degeneration
Source: Bone Res. 2025 Aug 12;13:72. doi: 10.1038/s41413-025-00449-6 (PMC12343876; doi:10.1038/s41413-025-00449-6)
Supplement: Supplementary file 1 — Supplemental Materials [file 41413_2025_449_MOESM1_ESM.docx]

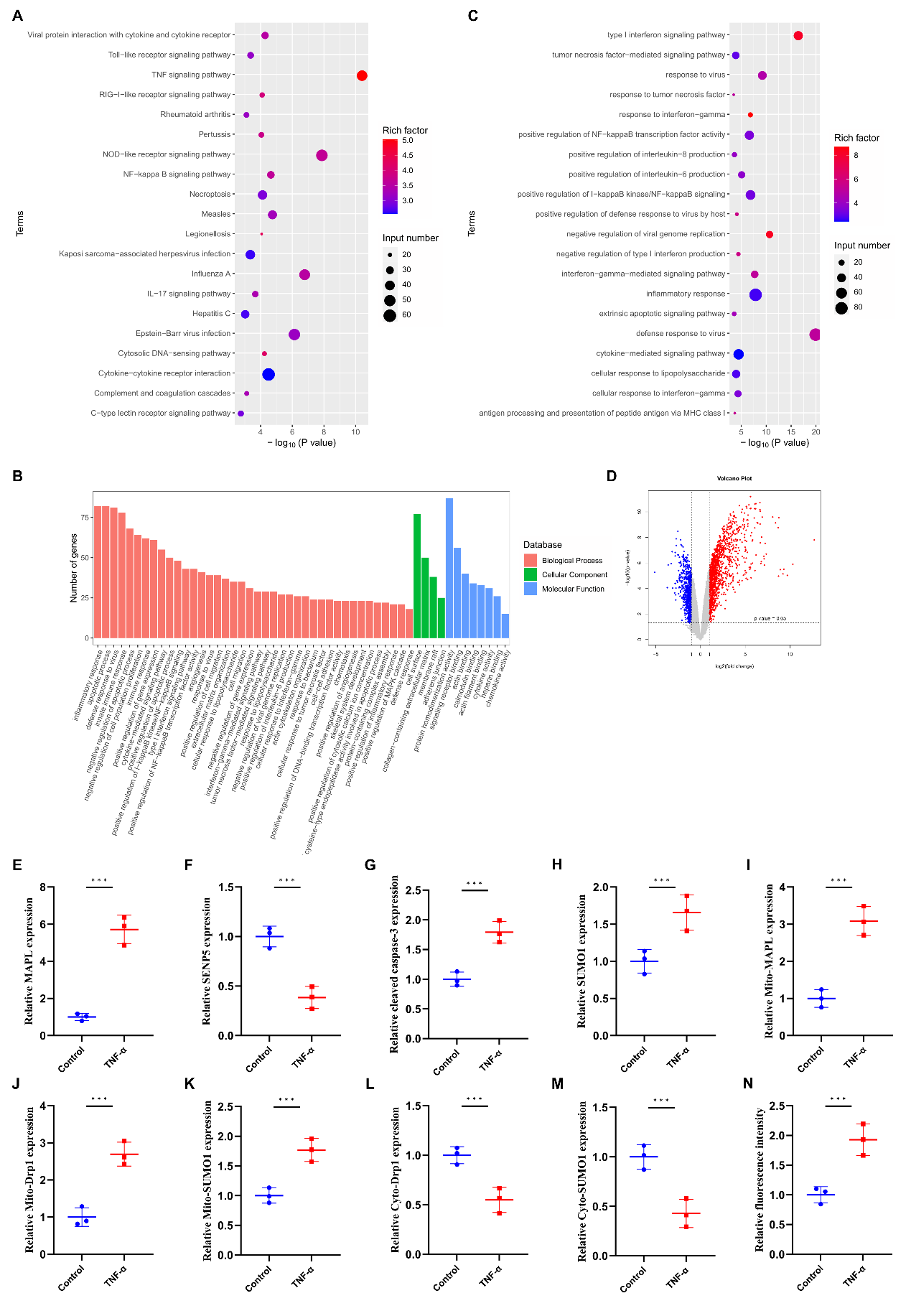


Figure S1 (A, B) Gene Ontology (GO) enrichment analysis (biological process, cellular component and molecular function) of NPCs treated with or without TNF-α. (C, D) Kyoto Encyclopedia of Genes and Genomes (KEGG) pathway analysis of the differential expressed genes shown in the volcano plot. (E-H) Normalized quantification of the expression levels of MAPL, SENP5, cleaved caspase-3 and SUMO1 in NPCs with the indicated treatment. (I-K) Normalized quantification of the expression levels of mitochondrial MAPL, Drp1 and SUMO1 in NPCs with the indicated treatment. (L, M) Normalized quantification of the expression levels of cytosolic Drp1 and SUMO1 in NPCs with the indicated treatment. (N) Normalized quantification of the fluorescence intensity of MitoSOX Red dyes in NPCs with the indicated treatment. (n = 3; *p < 0.05, **p < 0.01, and ***p < 0.001)


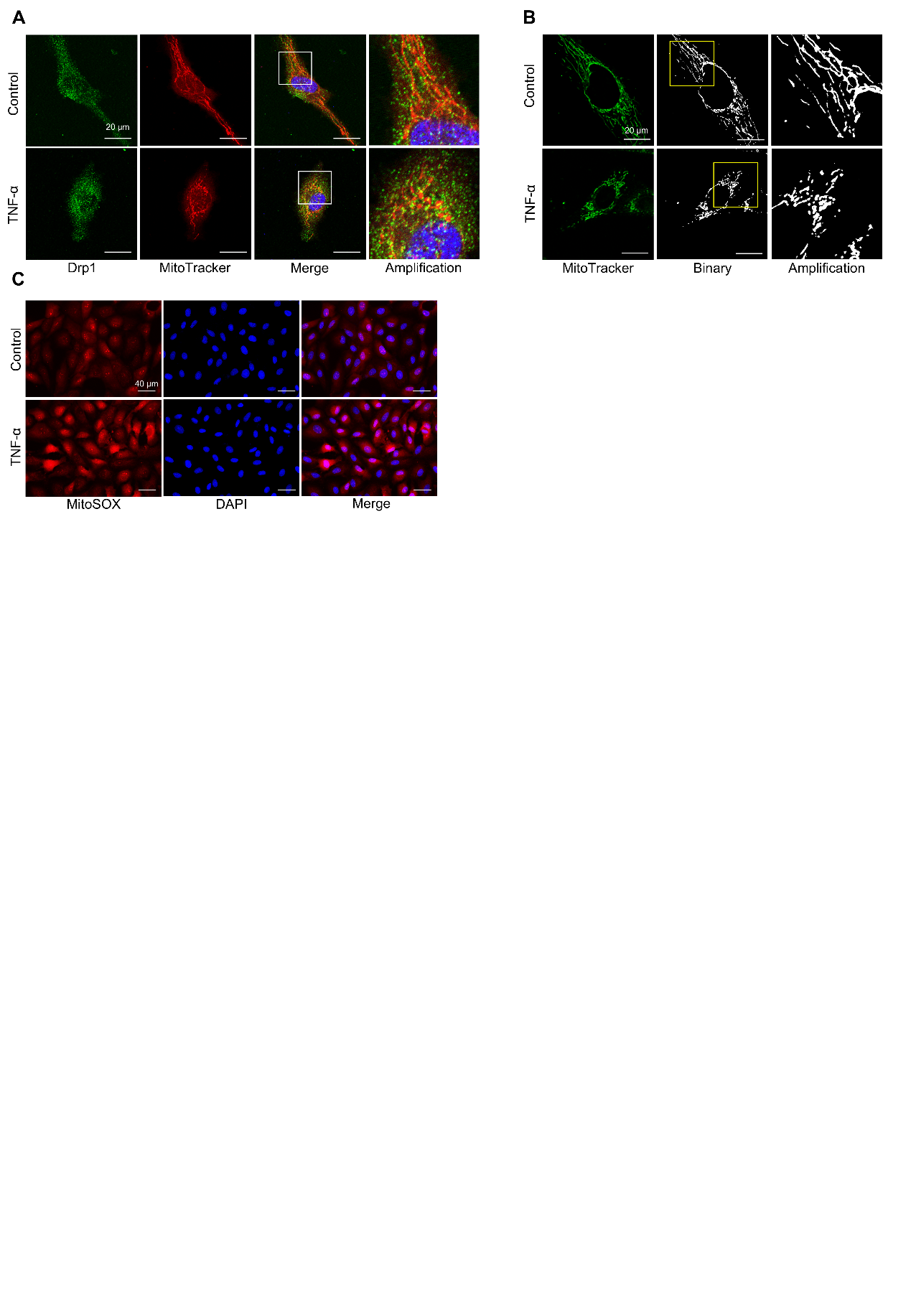


Figure S2 (A) MitoTracker Red staining and immunofluorescence staining for Drp1 in NPCs after treatment with TNF-α. (B) MitoTracker Green staining showing the mitochondrial morphology in NPCs after treatment with TNF-α. (C) The accumulation of mitochondrial ROS in NPCs was measured via MitoSOX Red staining. (n = 3; *p < 0.05, **p < 0.01, and ***p < 0.001)


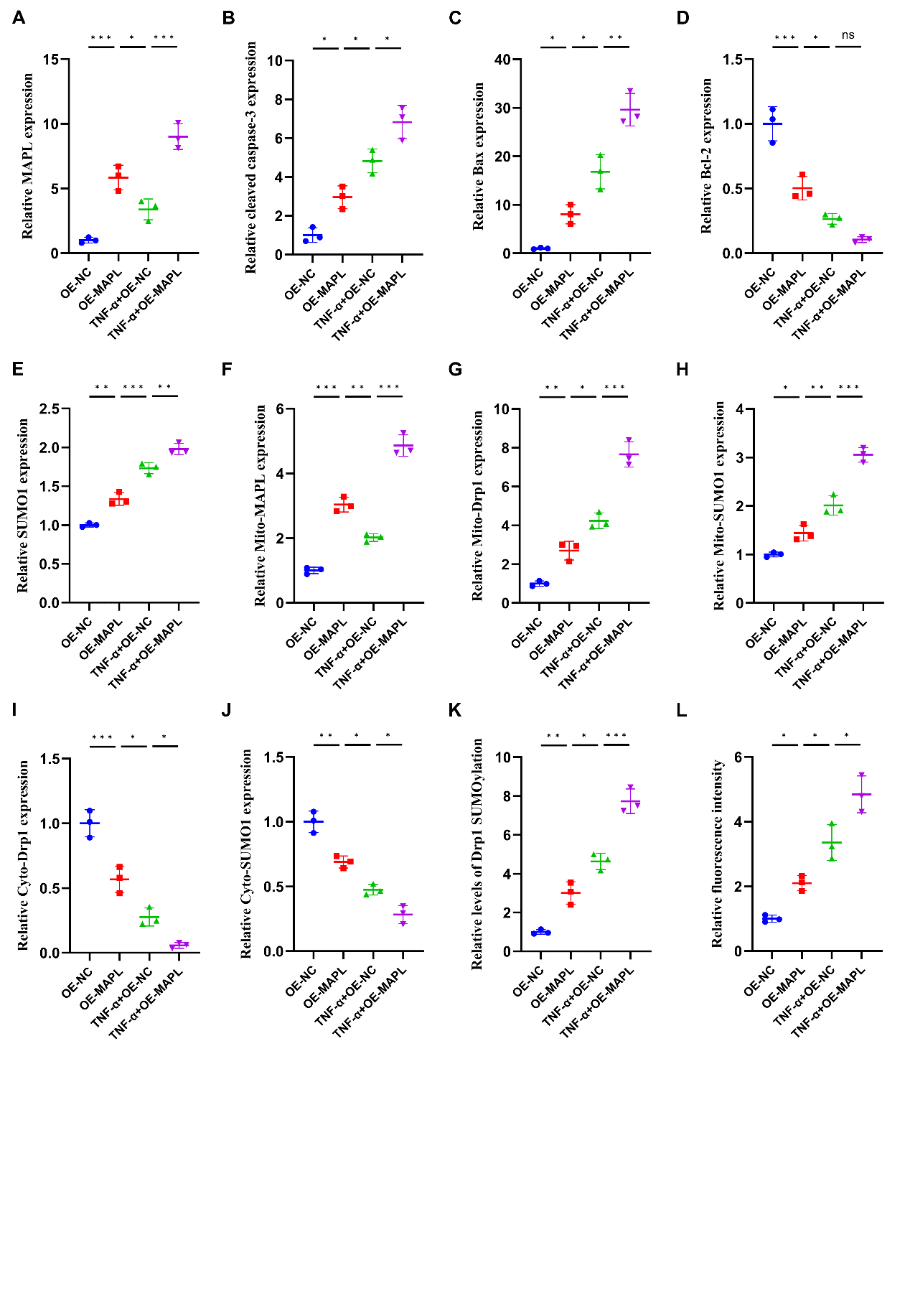


Figure S3 (A-E) Normalized quantification of the expression levels of MAPL, cleaved caspase-3, Bax, Bcl-2 and SUMO1 in NPCs with the indicated treatment. (F-H) Normalized quantification of the expression levels of mitochondrial MAPL, Drp1 and SUMO1 in NPCs with the indicated treatment. (I, J) Normalized quantification of the expression levels of cytosolic Drp1 and SUMO1 in NPCs with the indicated treatment. (K) Normalized quantification of the levels of Drp1 SUMOylation in NPCs with the indicated treatment. (L) Normalized quantification of the fluorescence intensity of MitoSOX Red dyes in NPCs with the indicated treatment. (n = 3; *p < 0.05, **p < 0.01, and ***p < 0.001)


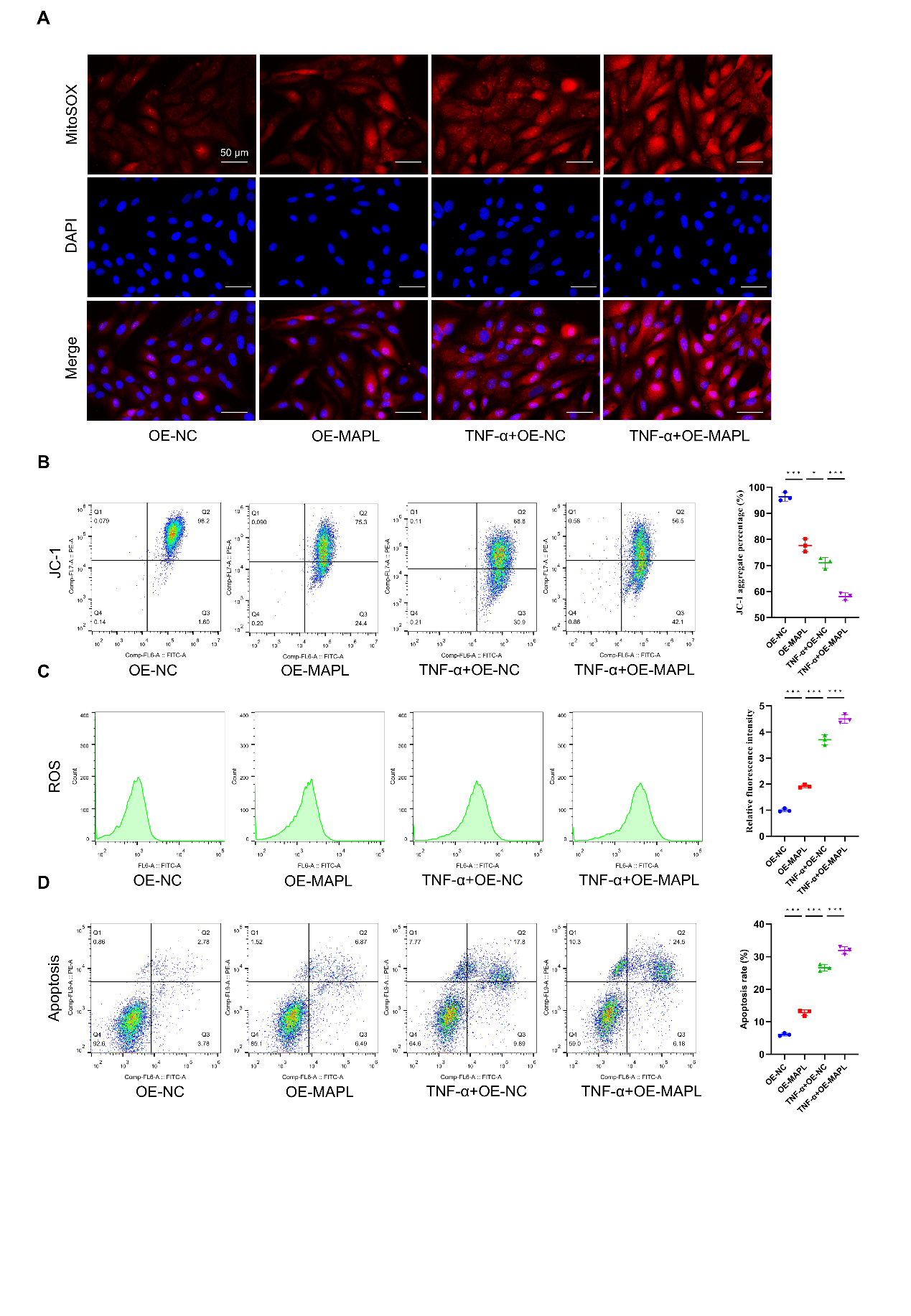


Figure S4 (A) MitoSOX Red staining revealed ROS production in NPC mitochondria after MAPL overexpression. (B) MAPL-induced ΔΨm loss in NPCs was assessed via JC-1 staining via flow cytometry. (C) Cellular ROS levels in NPCs transfected with the OE-MAPL plasmid were detected by DCFH-DA staining and flow cytometry. (D) Flow cytometry with Annexin V-FITC/PI staining was used to verify the percentage of apoptotic NPCs after MAPL was overexpressed. (n = 3; *p < 0.05, **p < 0.01, and ***p < 0.001)


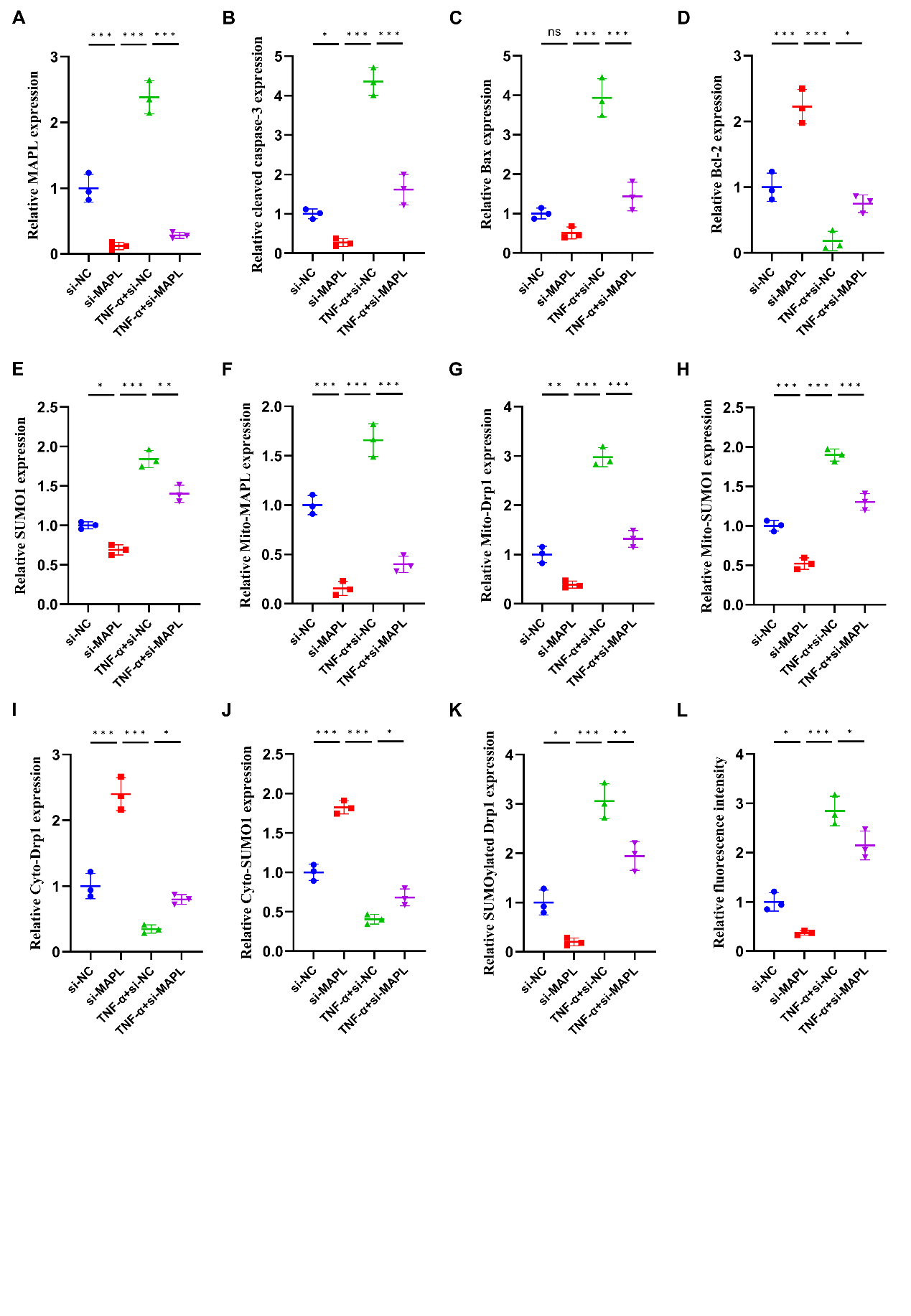


Figure S5 (A-E) Normalized quantification of the expression levels of MAPL, cleaved caspase-3, Bax, Bcl-2 and SUMO1 in NPCs with the indicated treatment. (F-H) Normalized quantification of the expression levels of mitochondrial MAPL, Drp1 and SUMO1 in NPCs with the indicated treatment. (I, J) Normalized quantification of the expression levels of cytosolic Drp1 and SUMO1 in NPCs with the indicated treatment. (K) Normalized quantification of the levels of Drp1 SUMOylation in NPCs with the indicated treatment. (L) Normalized quantification of the fluorescence intensity of MitoSOX Red dyes in NPCs with the indicated treatment. (n = 3; *p < 0.05, **p < 0.01, and ***p < 0.001)


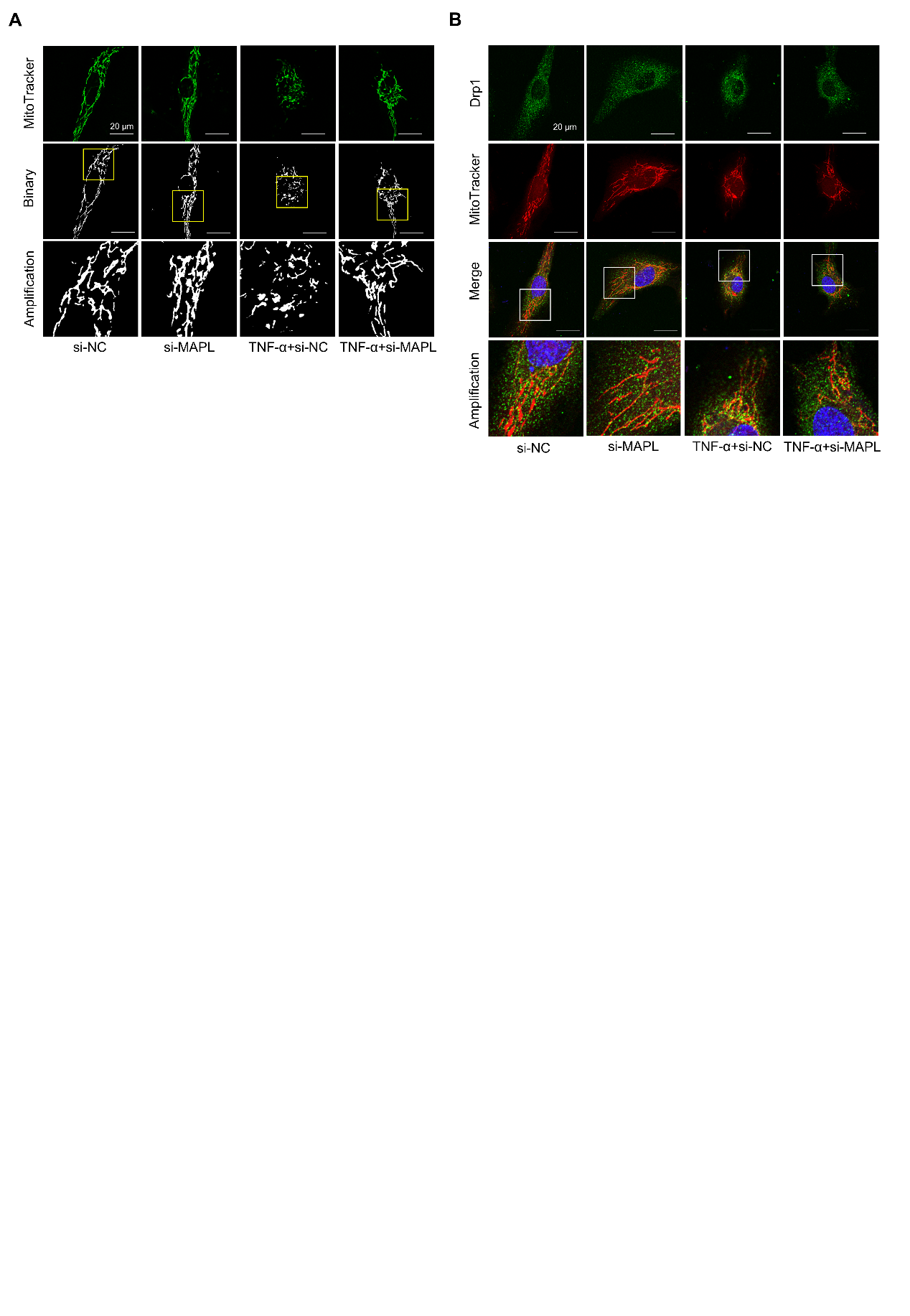


Figure S6 (A) Mitochondrial morphology of NPCs after MAPL inhibition, as shown by MitoTracker Green staining. (B) The subcellular distribution of Drp1 was measured by MitoTracker Red staining and immunofluorescence staining for Drp1 in NPCs after MAPL was inhibited. (n = 3; *p < 0.05, **p < 0.01, and ***p < 0.001)


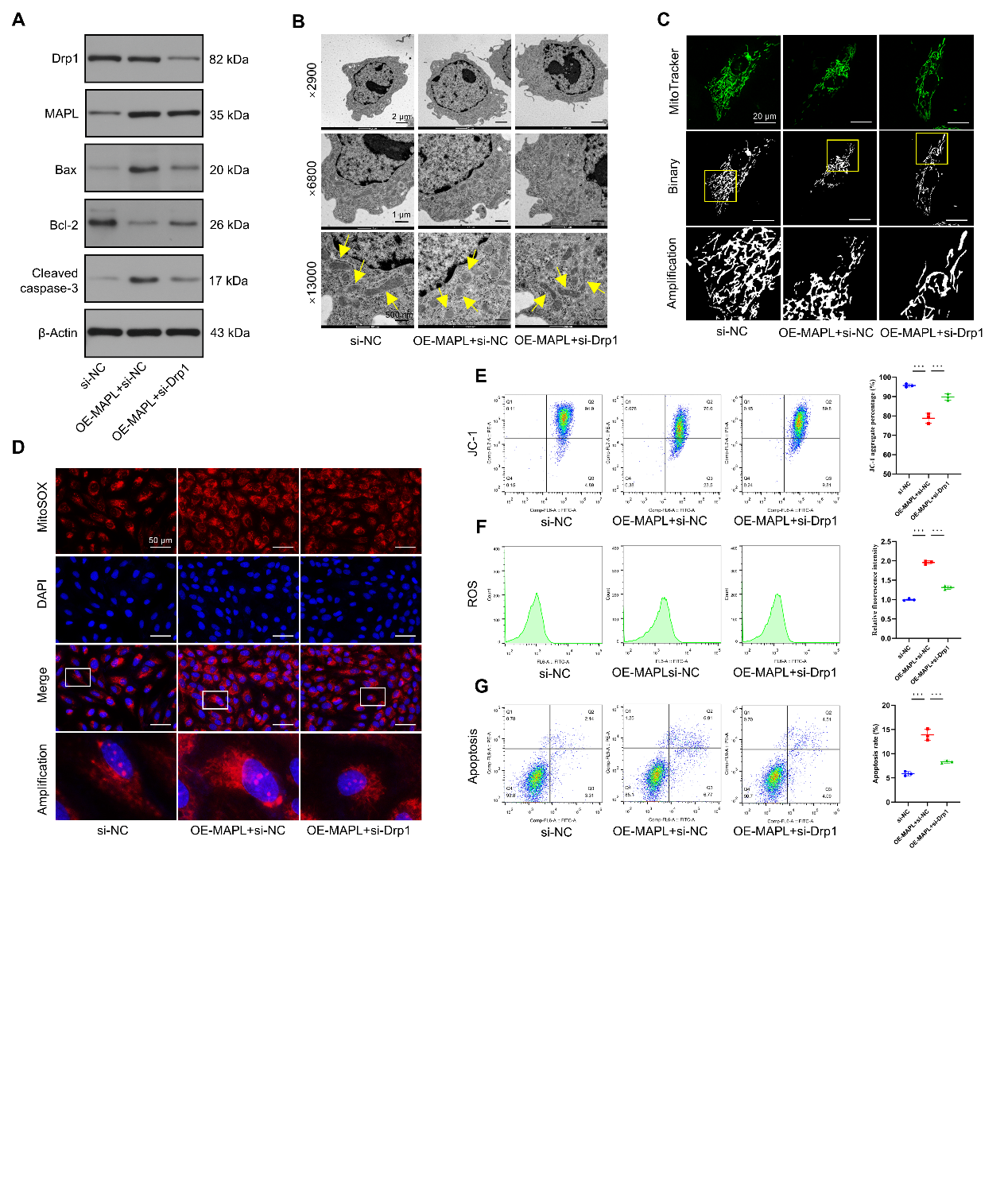


Figure S7 (A) Western blot analysis of Drp1, MAPL, cleaved caspase-3, Bax and Bcl-2 levels in NPCs transfected with the MAPL plasmid and si-Drp1. (B) Ultrastructural analysis of mitochondria in si-Drp1-treated NPCs after MAPL overexpression. (C) MitoTracker Red staining showing the mitochondrial morphology in NPCs. (D) ROS production in NPC mitochondria after transfection with the MAPL plasmid and si-Drp1 was measured by MitoSOX staining. (E) JC-1 staining and flow cytometry were used to detect the ΔΨm in NPCs after MAPL overexpression and Drp1 silencing. (F) ROS accumulation in NPCs was assessed by DCFH-DA staining and flow cytometry. (G) Flow cytometry with Annexin V-FITC/PI staining of apoptosis in NPCs transfected with the MAPL plasmid and si-Drp1. (n = 3; *p < 0.05, **p < 0.01, and ***p < 0.001)


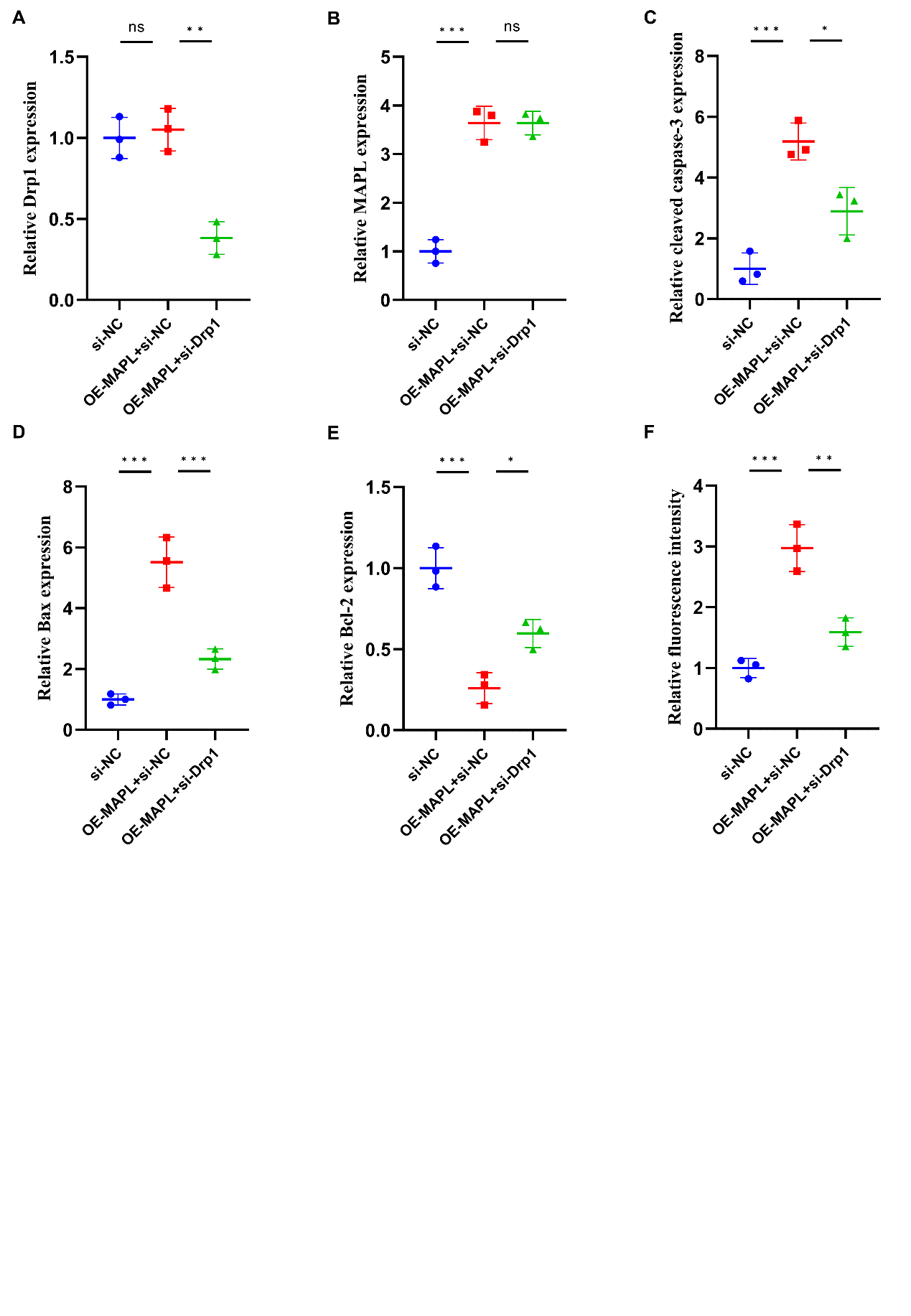


Figure S8 (A-E) Normalized quantification of the expression levels of Drp1, MAPL, cleaved caspase-3, Bax and Bcl-2 in NPCs with the indicated treatment. (F) Normalized quantification of the fluorescence intensity of MitoSOX Red dyes in NPCs with the indicated treatment. (n = 3; *p < 0.05, **p < 0.01, and ***p < 0.001)


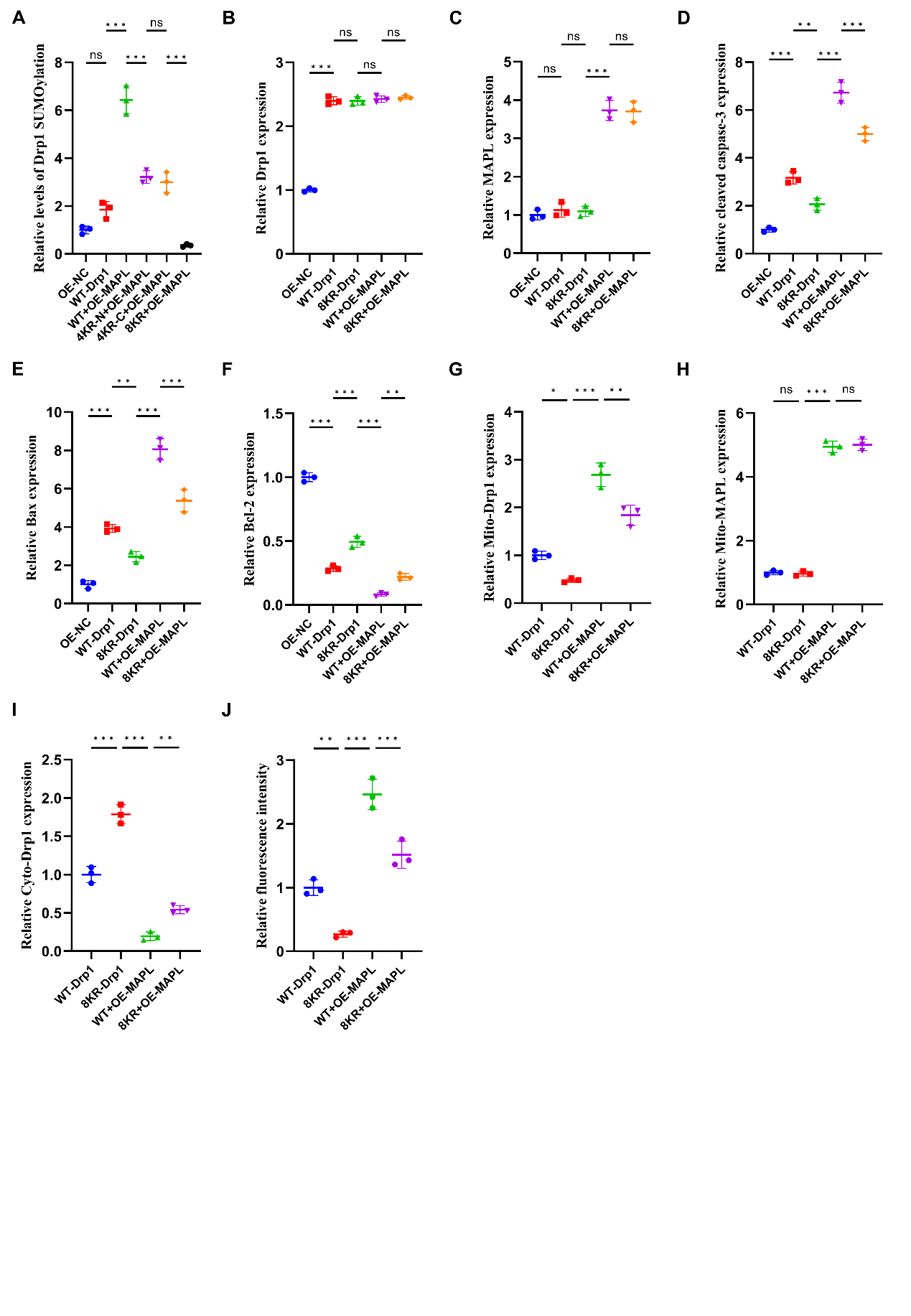


Figure S9 (A) Normalized quantification of the levels of Drp1 SUMOylation in NPCs with the indicated treatment. (B-F) Normalized quantification of the expression levels of Drp1, MAPL, cleaved caspase-3, Bax and Bcl-2 in NPCs with the indicated treatment. (G, H) Normalized quantification of the expression levels of mitochondrial Drp1 and MAPL in NPCs with the indicated treatment. (I) Normalized quantification of the expression levels of cytosolic Drp1 in NPCs with the indicated treatment. (J) Normalized quantification of the fluorescence intensity of MitoSOX Red dyes in NPCs with the indicated treatment. (n = 3; *p < 0.05, **p < 0.01, and ***p < 0.001)


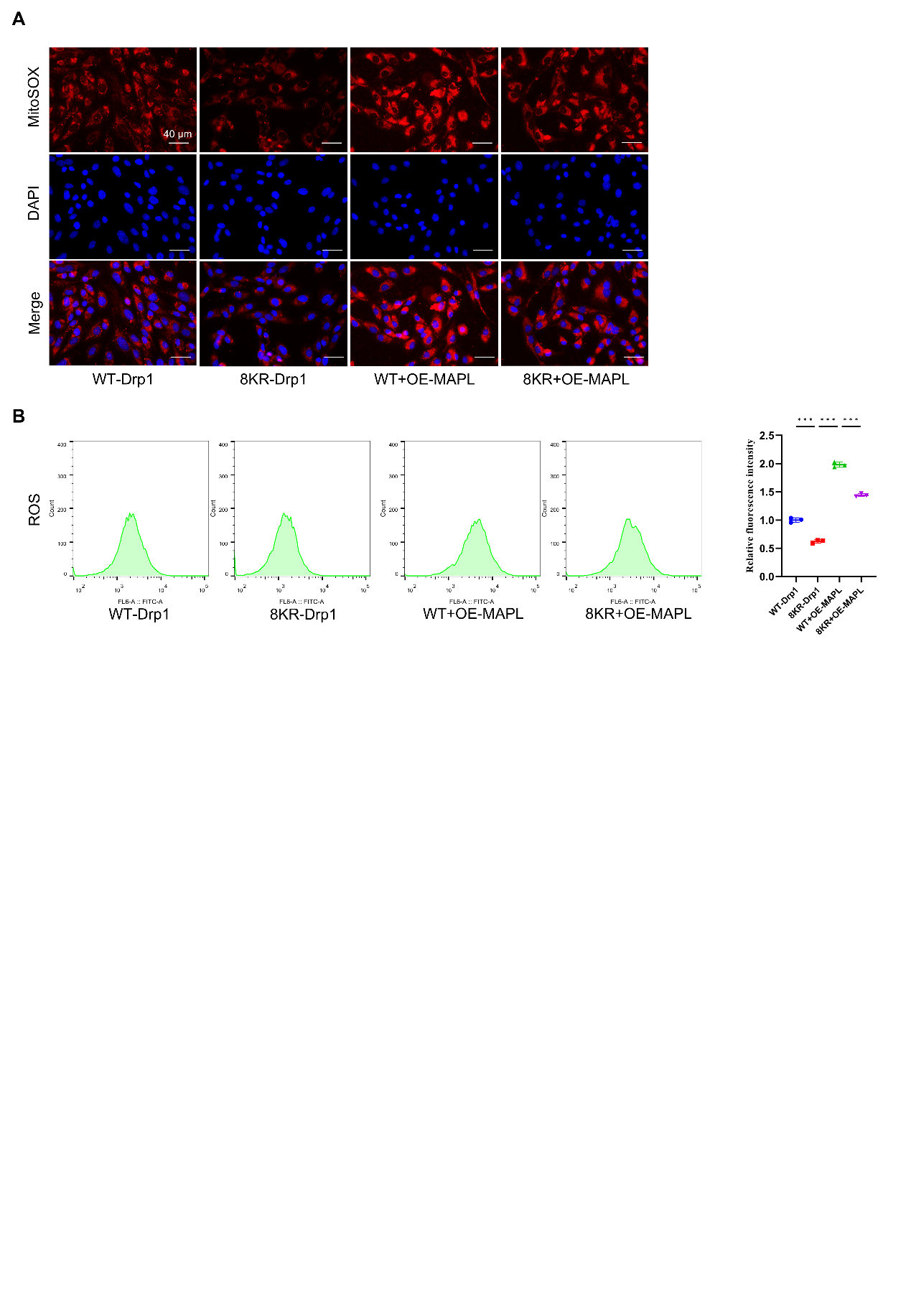


Figure S10 (A) Mitochondrial ROS production in MAPL-overexpressing NPCs after transfection with WT-Drp1 or 8KR was measured by MitoSOX staining. (B) ROS accumulation in NPCs was assessed by DCFH-DA staining and flow cytometry. (n = 3; *p < 0.05, **p < 0.01, and ***p < 0.001)


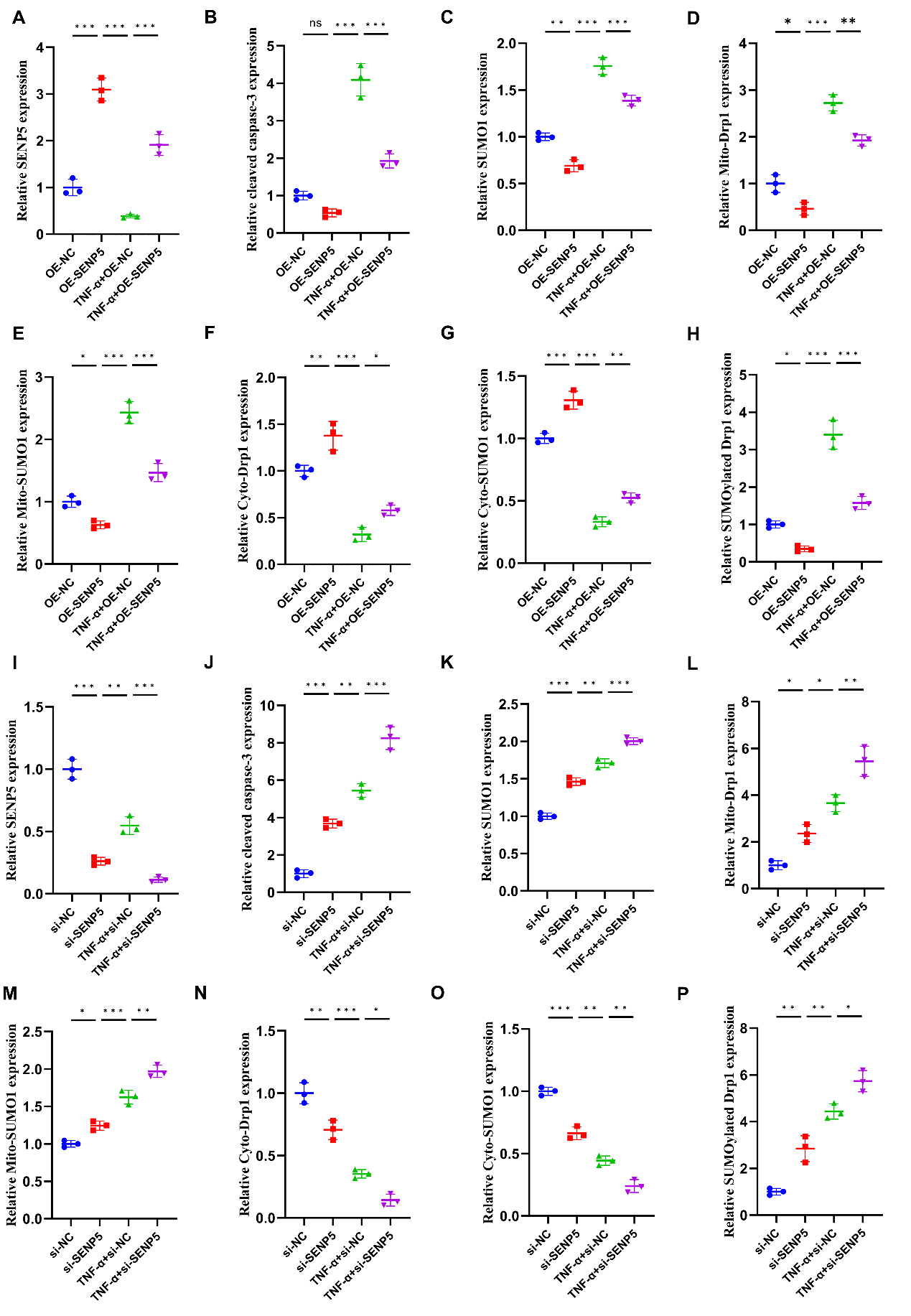


Figure S11 (A-C) Normalized quantification of the expression levels of SENP5, cleaved caspase-3 and SUMO1 in NPCs with the indicated treatment. (D, E) Normalized quantification of the expression levels of mitochondrial Drp1 and SUMO1 in NPCs with the indicated treatment. (F, G) Normalized quantification of the expression levels of cytosolic Drp1 and SUMO1 in NPCs with the indicated treatment. (H) Normalized quantification of the levels of Drp1 SUMOylation in NPCs with the indicated treatment. (I-K) Normalized quantification of the expression levels of SENP5, cleaved caspase-3 and SUMO1 in NPCs with the indicated treatment. (L, M) Normalized quantification of the expression levels of mitochondrial Drp1 and SUMO1 in NPCs with the indicated treatment. (N, O) Normalized quantification of the expression levels of cytosolic Drp1 and SUMO1 in NPCs with the indicated treatment. (P) Normalized quantification of the levels of Drp1 SUMOylation in NPCs with the indicated treatment. (n = 3; *p < 0.05, **p < 0.01, and ***p < 0.001)


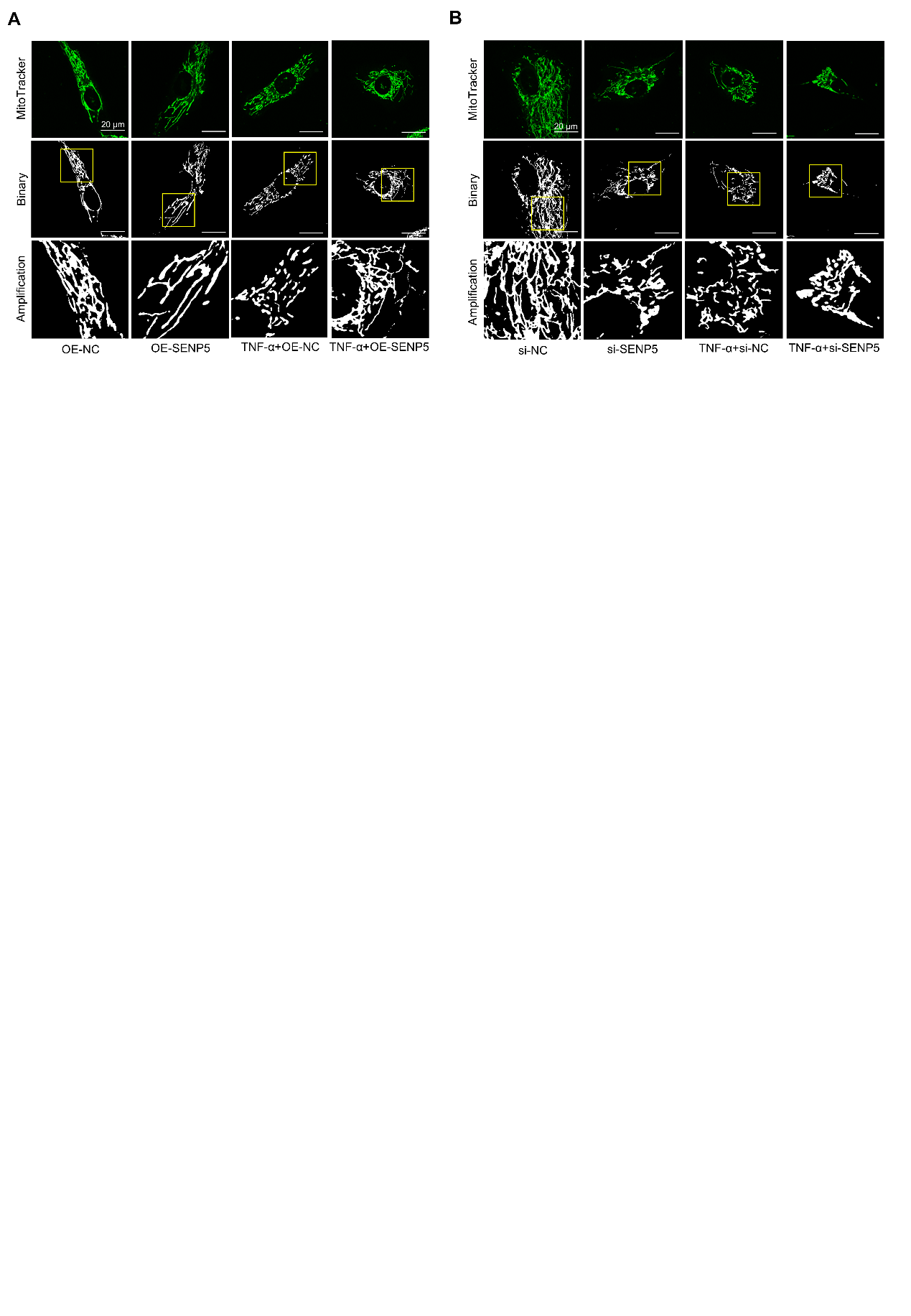


Figure S12 (A, B) Mitochondrial morphology in NPCs transfected with OE-SENP5 or si-SENP5 was observed using MitoTracker Green staining. (n = 3; *p < 0.05, **p < 0.01, and ***p < 0.001)


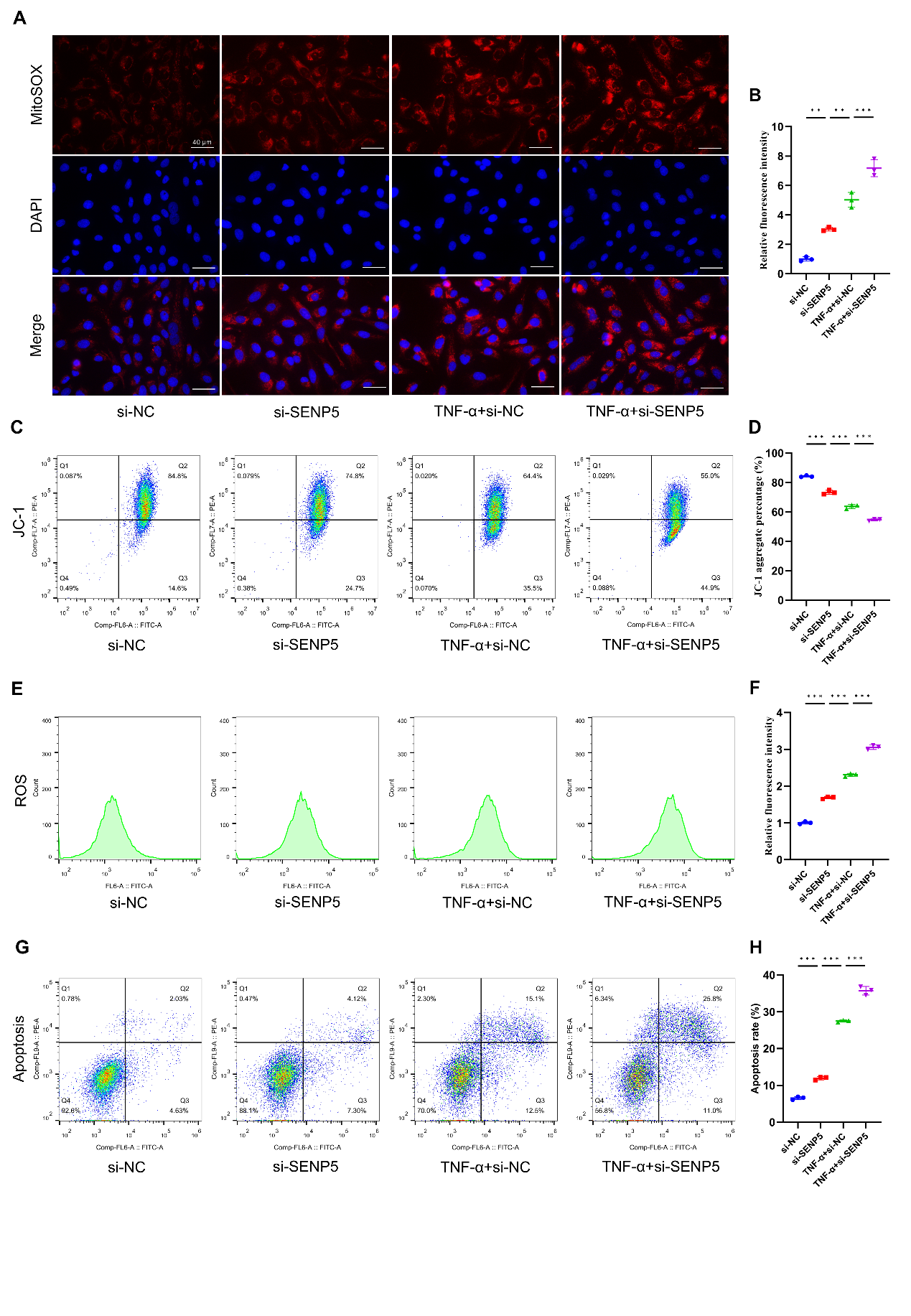


Figure S13 (A, B) MitoSOX Red staining indicated mitochondrial ROS accumulation in NPCs after SENP5 knockdown. (C, D) ΔΨm loss in NPCs was assessed by JC-1 staining and flow cytometry. (E, F) Cellular ROS levels in NPCs after SENP5 silencing were detected by DCFH-DA staining and flow cytometry. (G, H) Flow cytometry with Annexin V-FITC/PI staining was used to verify the percentage of apoptotic NPCs after SENP5 knockdown. (n = 3; *p < 0.05, **p < 0.01, and ***p < 0.001)


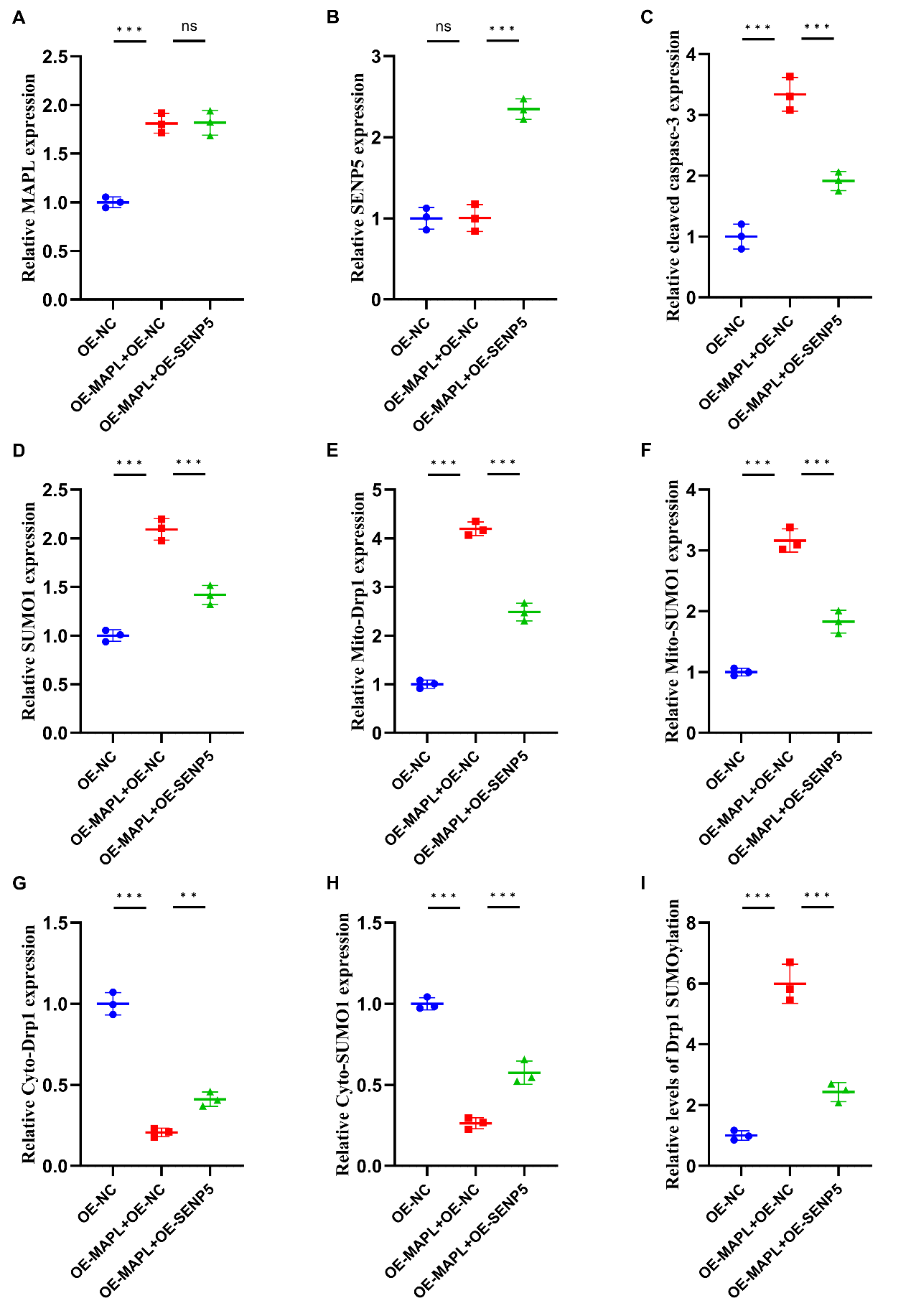


Figure S14 (A-D) Normalized quantification of the expression levels of MAPL, SENP5, cleaved caspase-3 and SUMO1 in NPCs with the indicated treatment. (E, F) Normalized quantification of the expression levels of mitochondrial Drp1 and SUMO1 in NPCs with the indicated treatment. (G, H) Normalized quantification of the expression levels of cytosolic Drp1 and SUMO1 in NPCs with the indicated treatment. (I) Normalized quantification of the levels of Drp1 SUMOylation in NPCs with the indicated treatment. (n = 3; *p < 0.05, **p < 0.01, and ***p < 0.001)


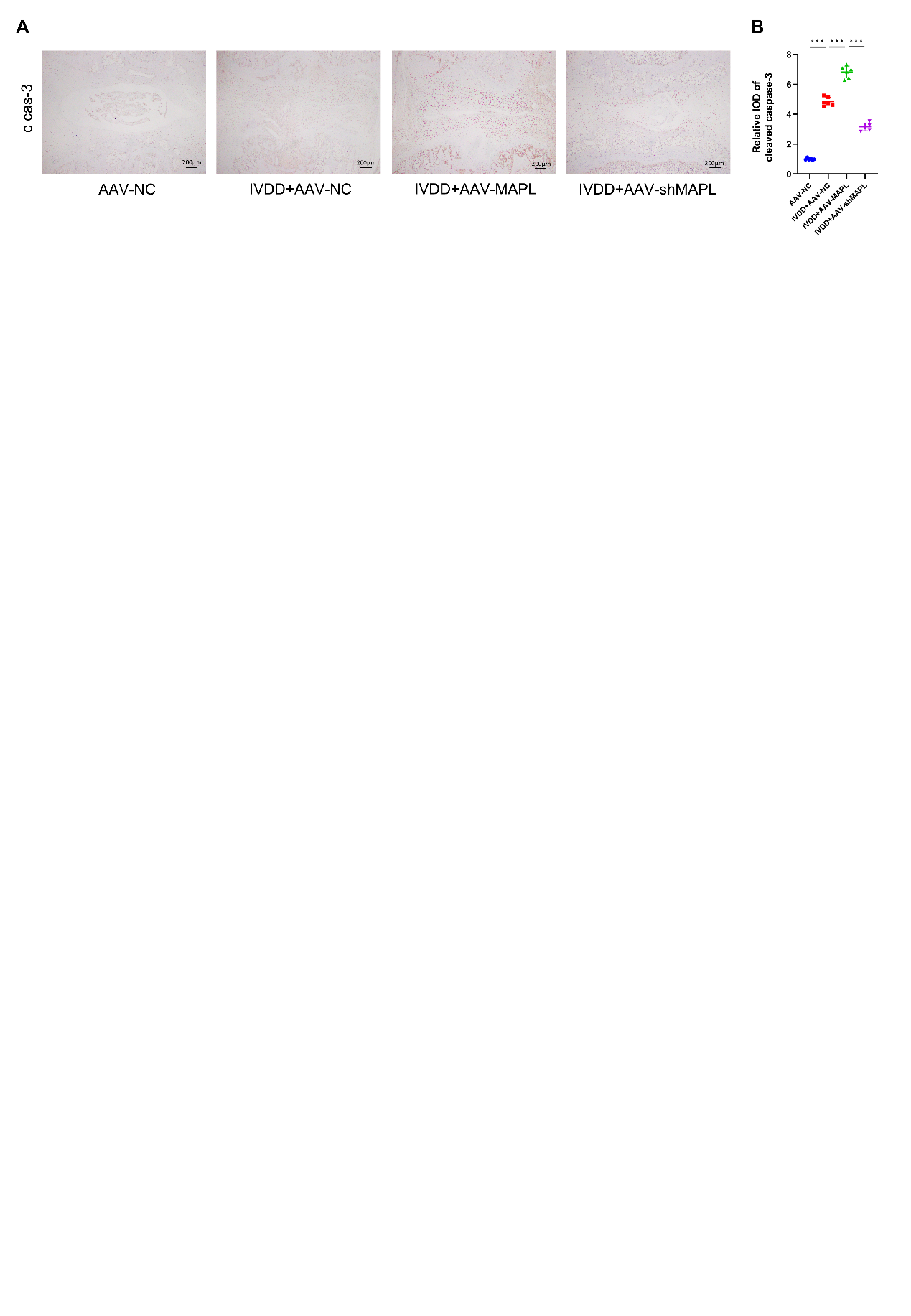


Figure S15 (A, B) Immunohistochemical analysis of cleaved caspase-3 levels. (n = 6; *p < 0.05, **p < 0.01, and ***p < 0.001)
