# Supplementary figures and images for: The mitochondrial E3 ligase MAPL SUMOylates Drp1 to facilitate mitochondrial fission in intervertebral disc degeneration
Source: Bone Res. 2025 Aug 12;13:72. doi: 10.1038/s41413-025-00449-6 (PMC12343876; doi:10.1038/s41413-025-00449-6)

**Figure 1**


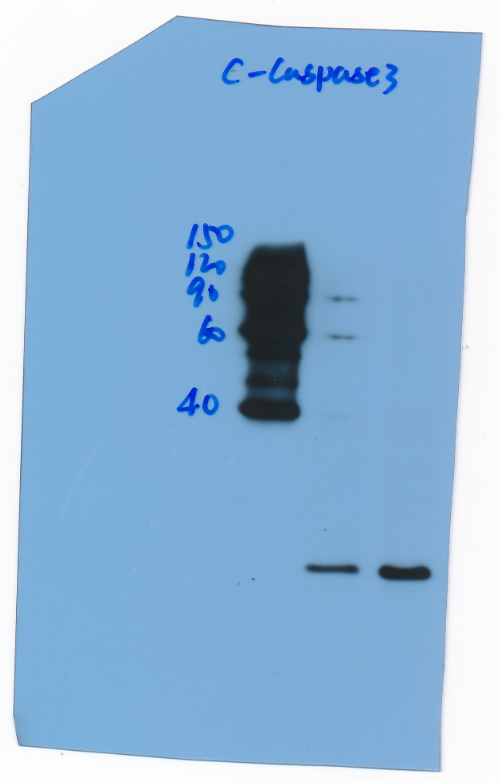

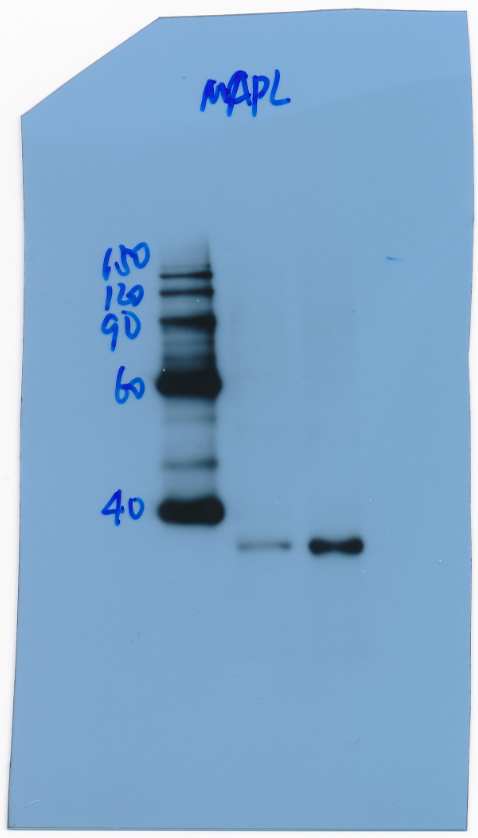

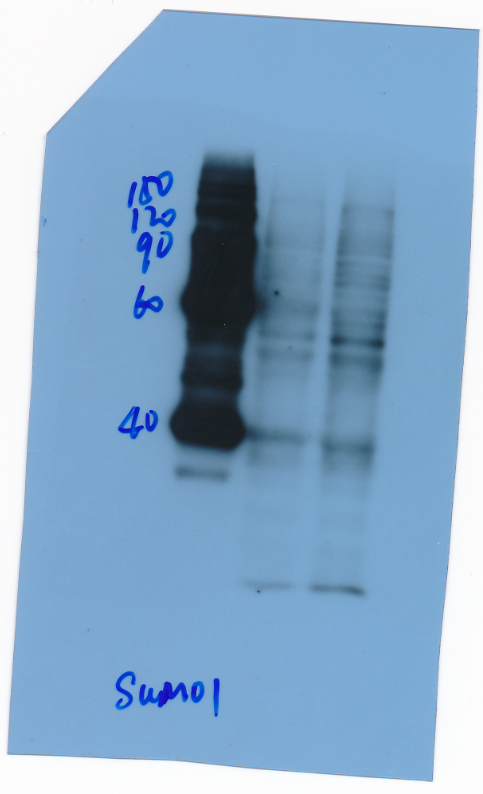

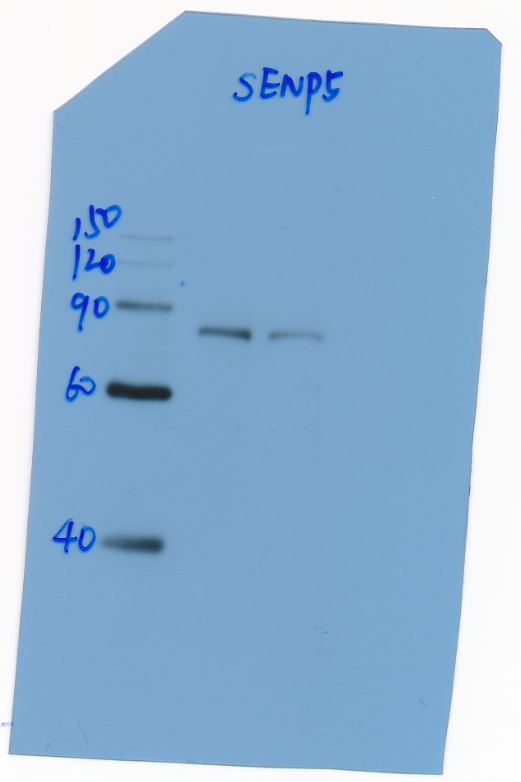

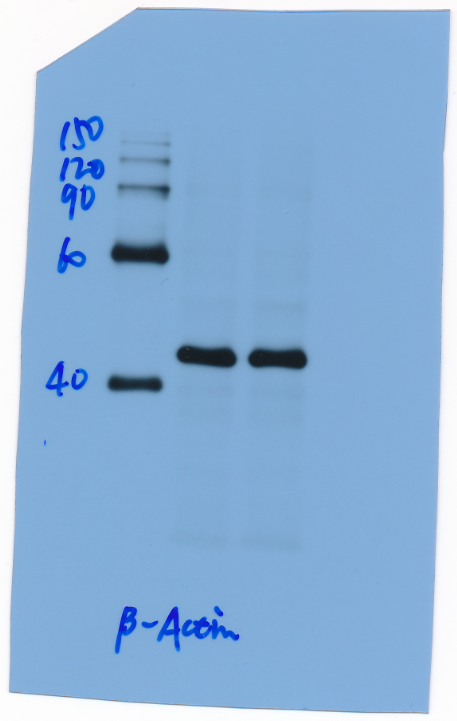


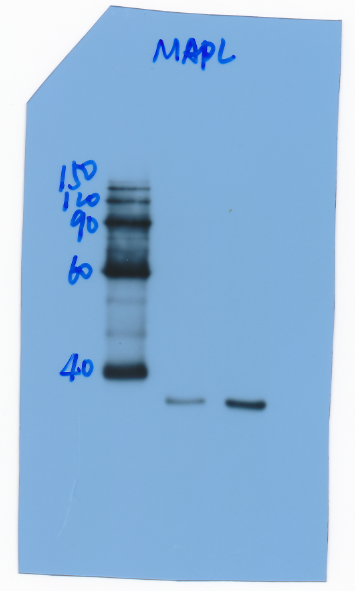

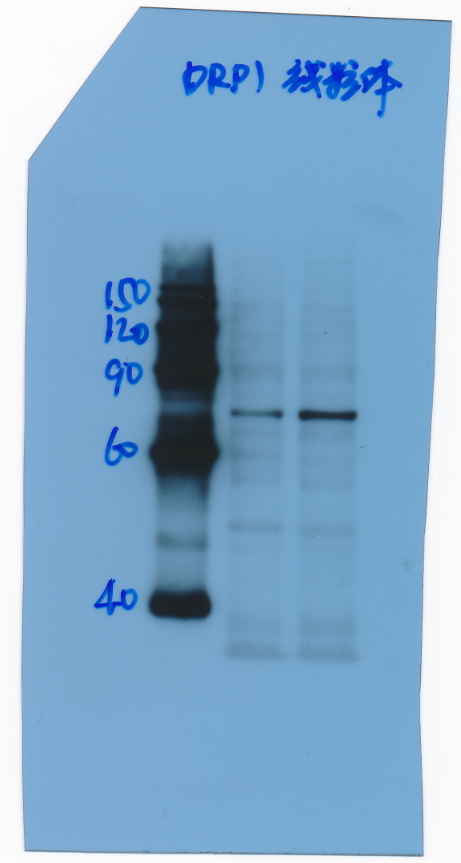

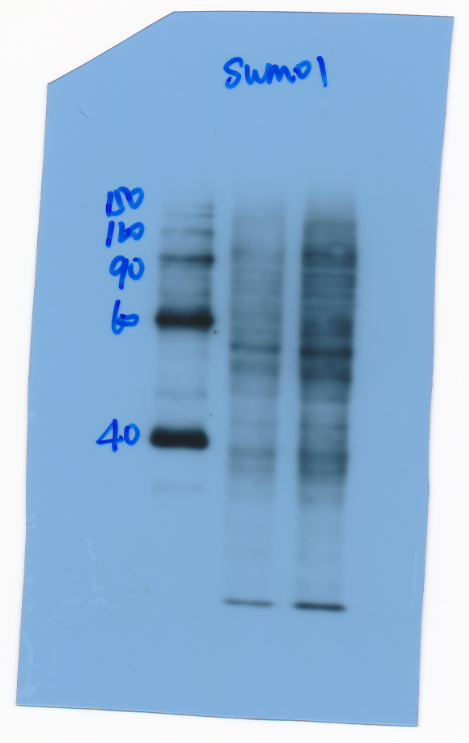

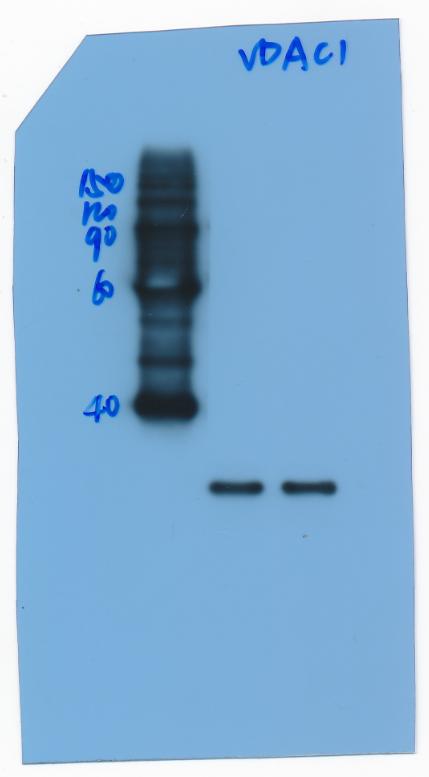


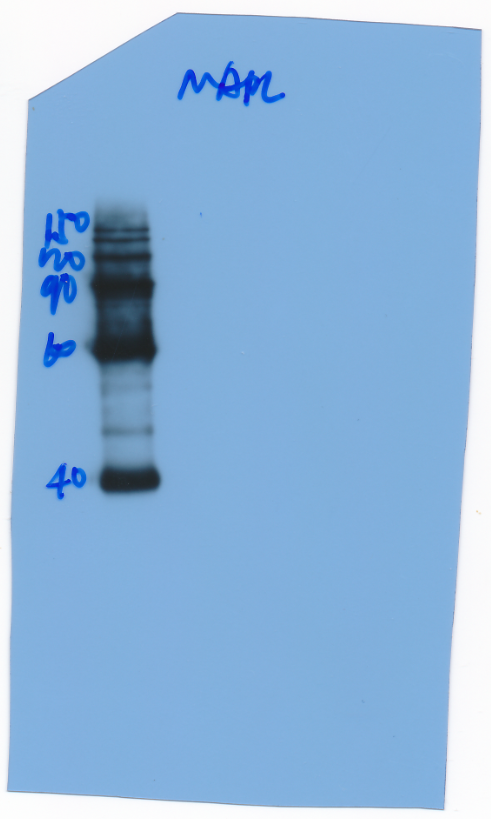

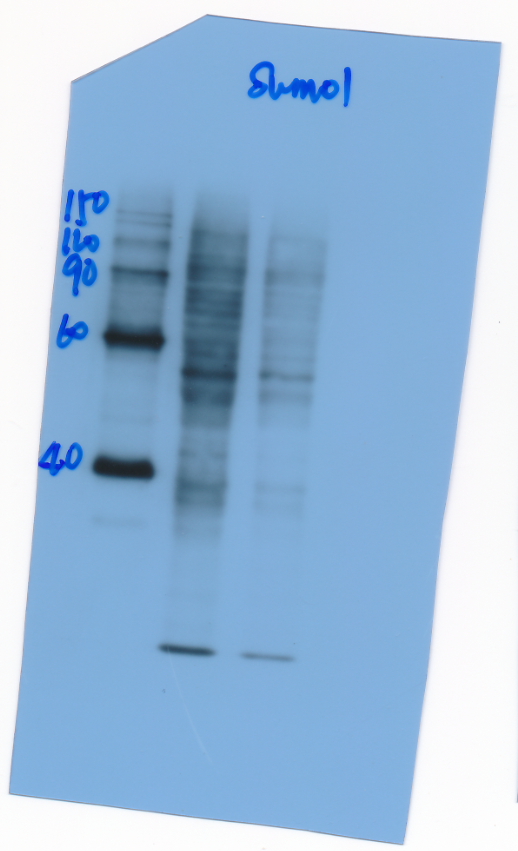

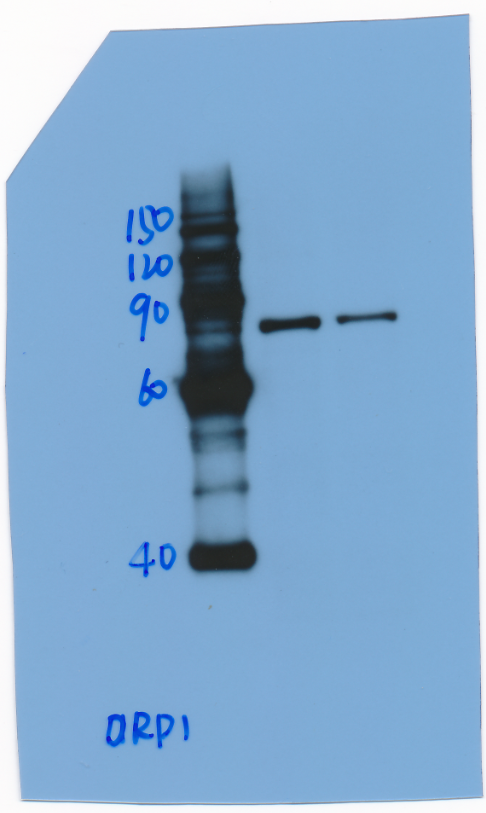


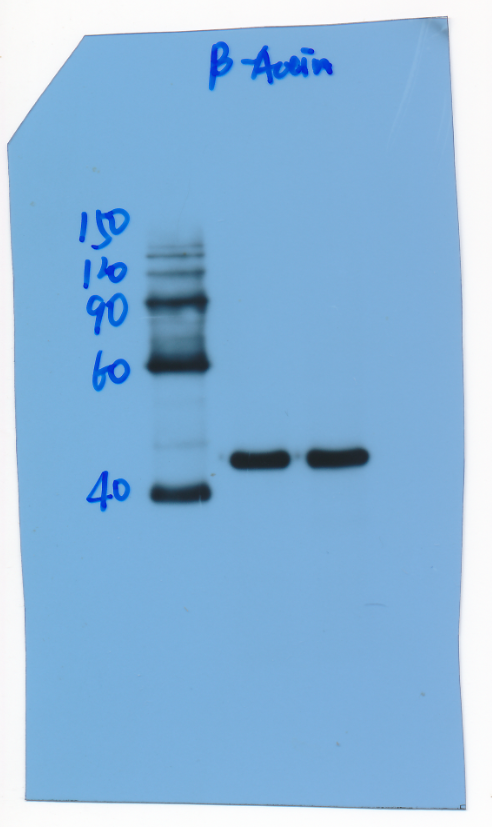


**Figure 2**


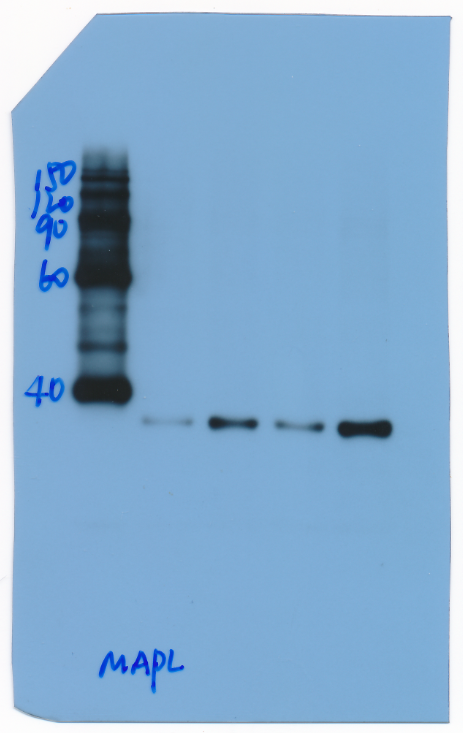

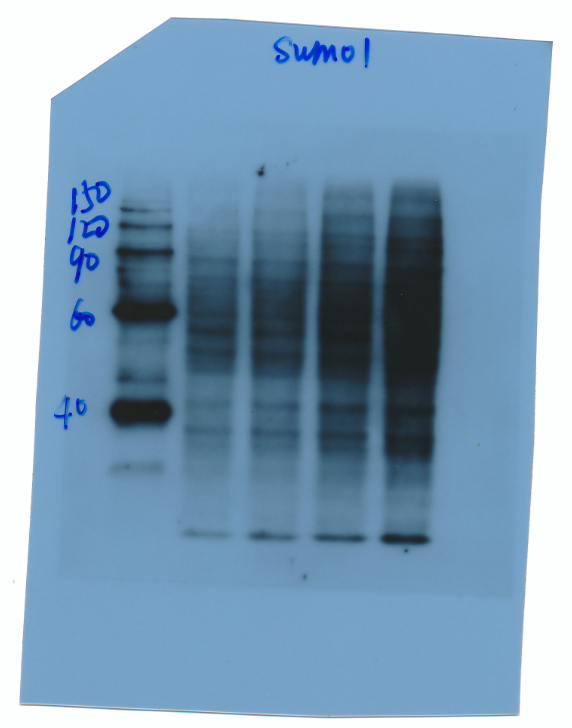

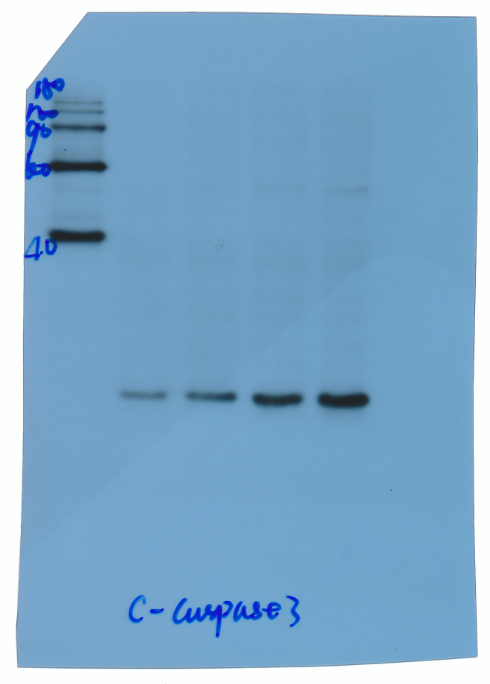

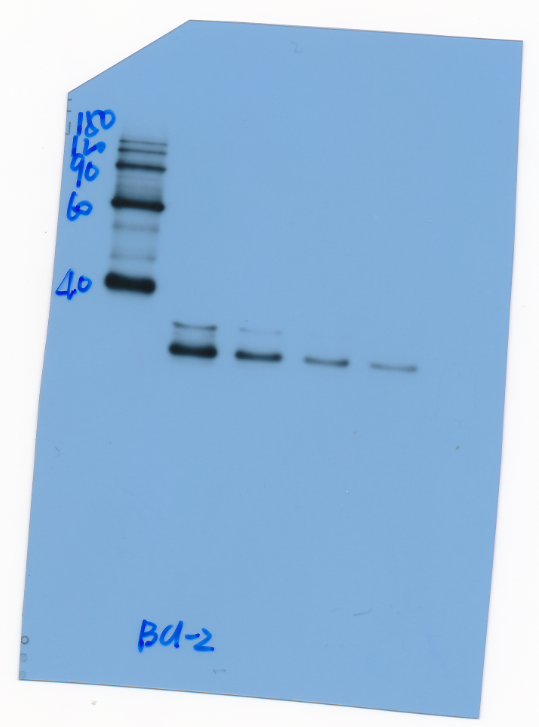

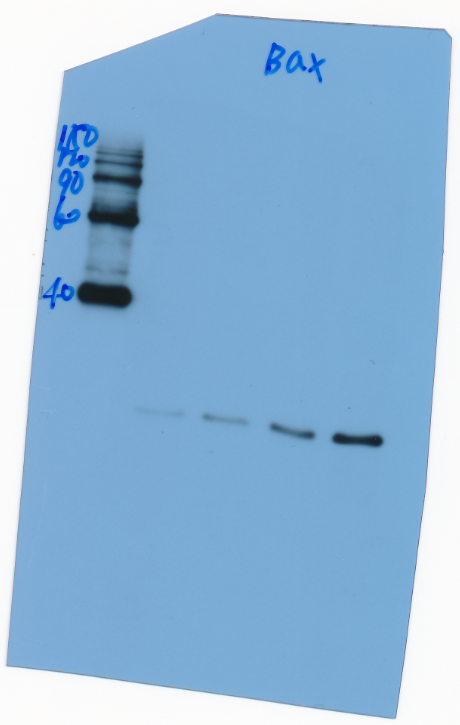

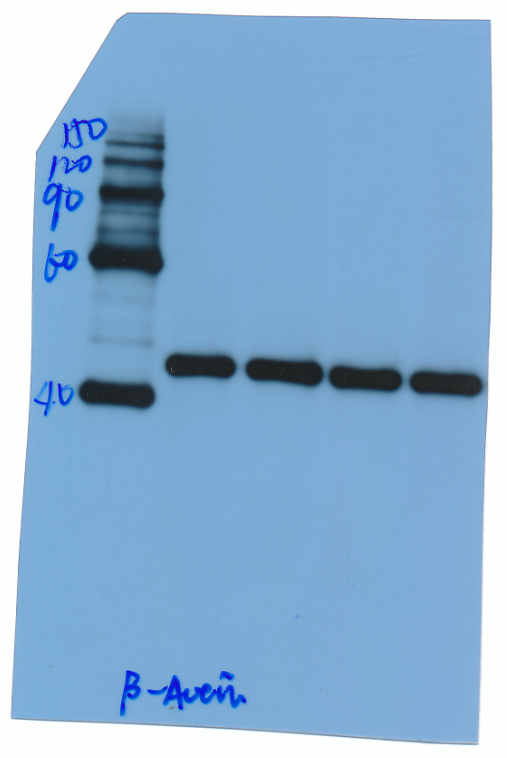


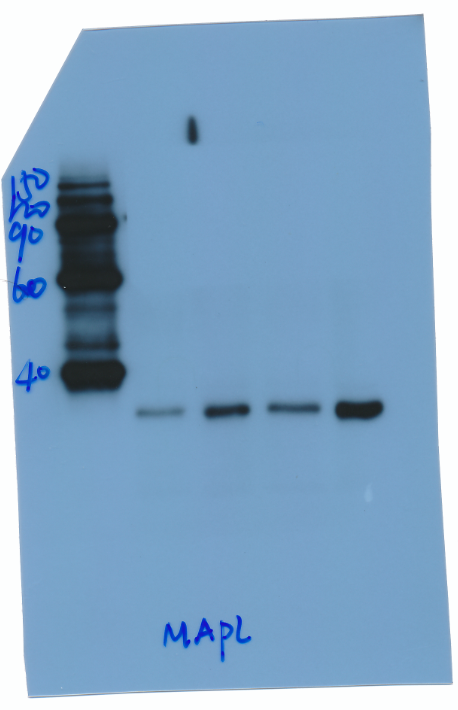

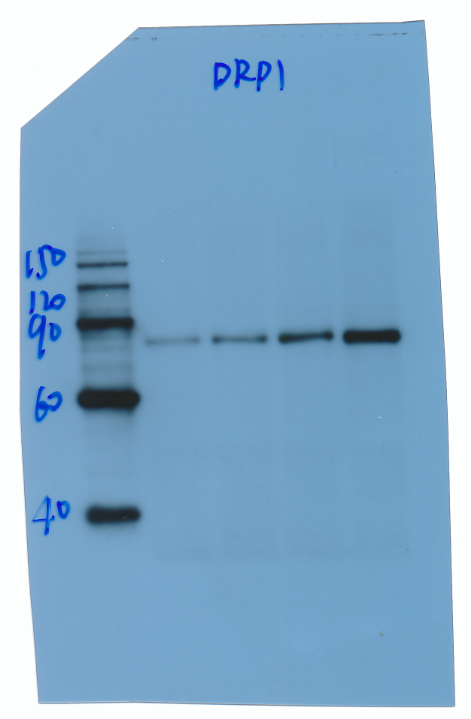

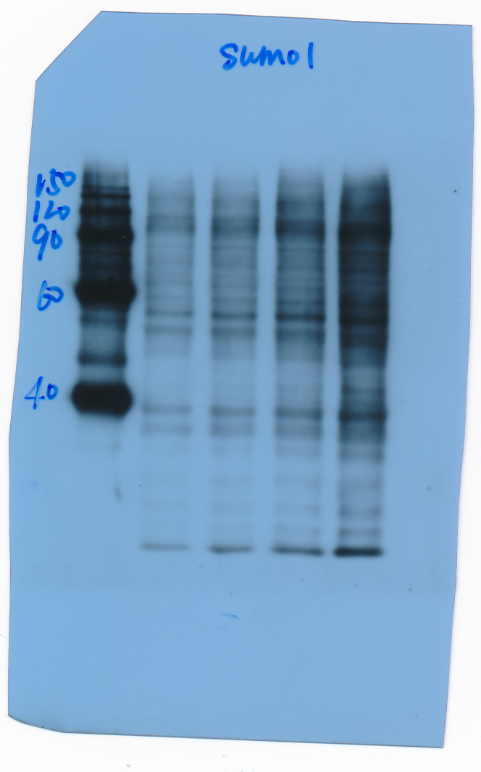

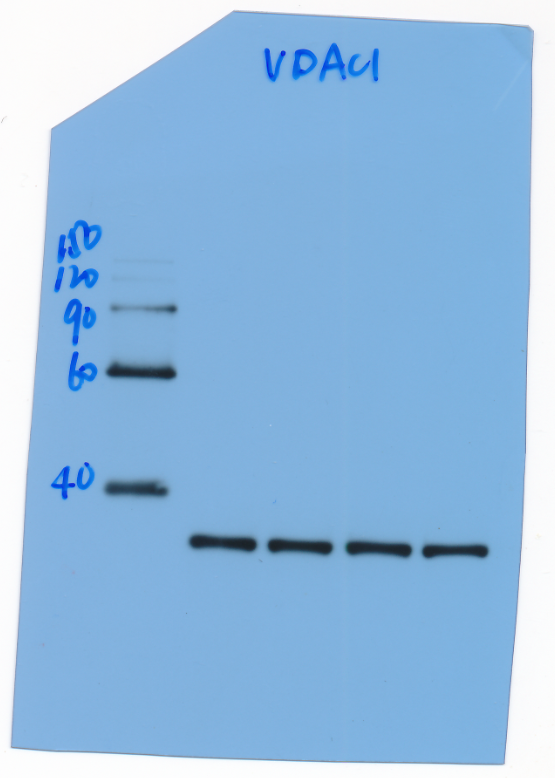


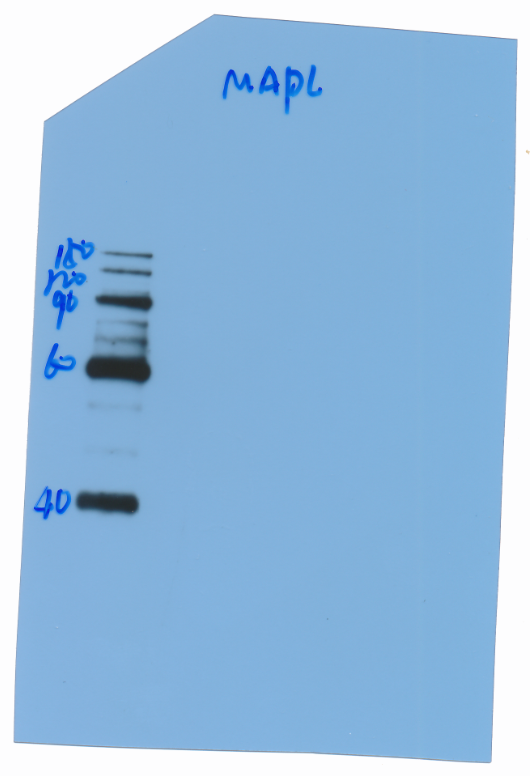

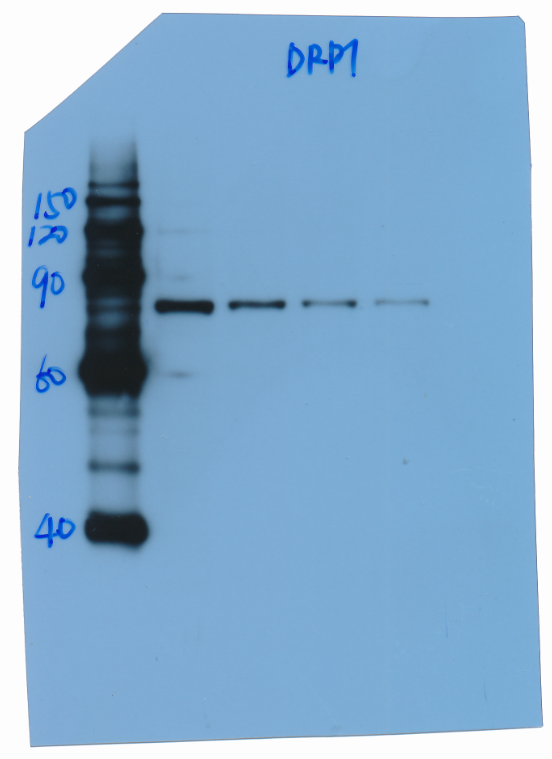

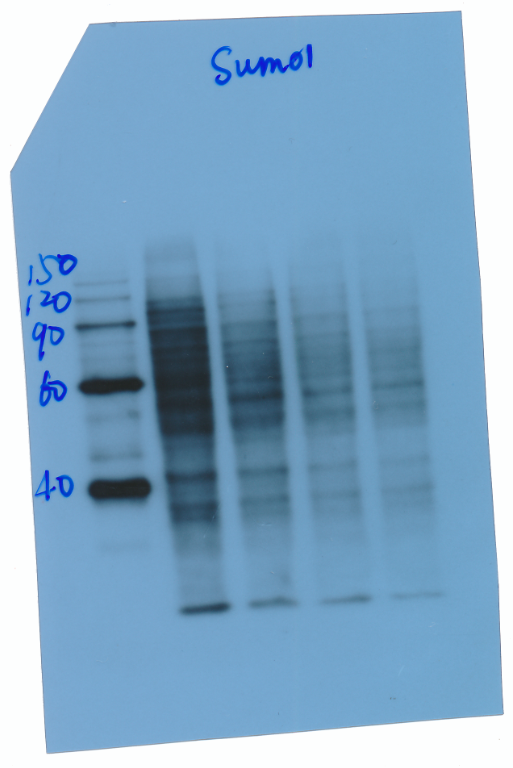

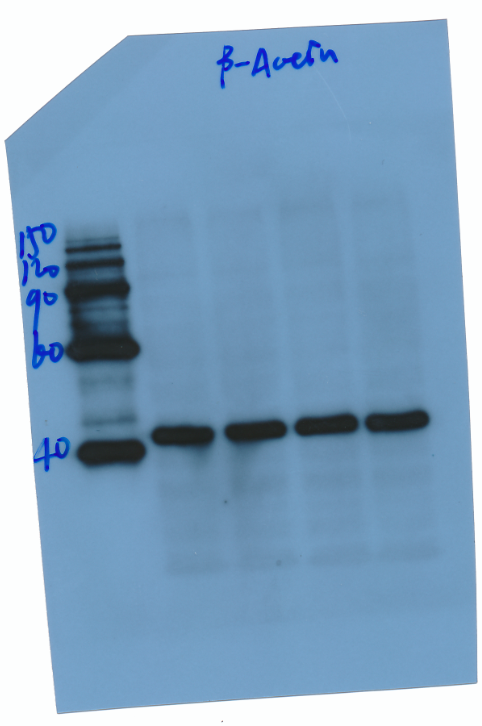


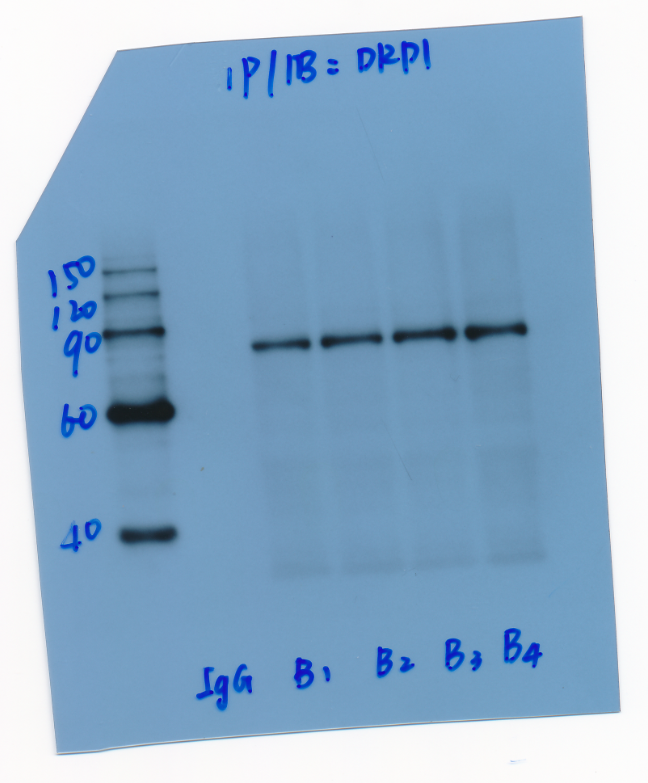

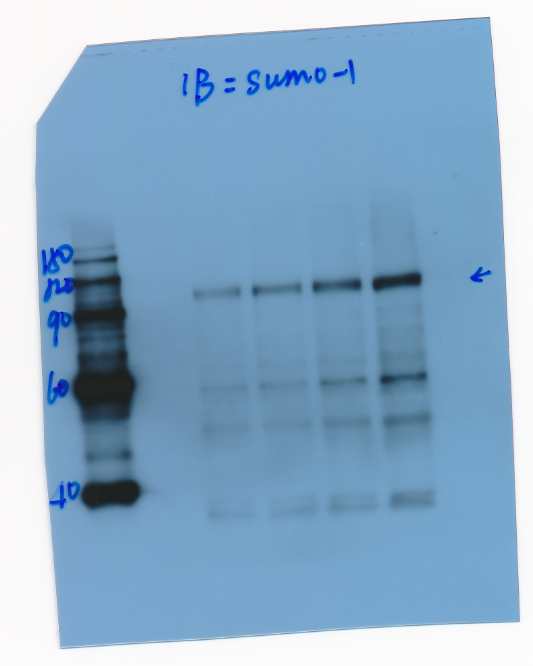

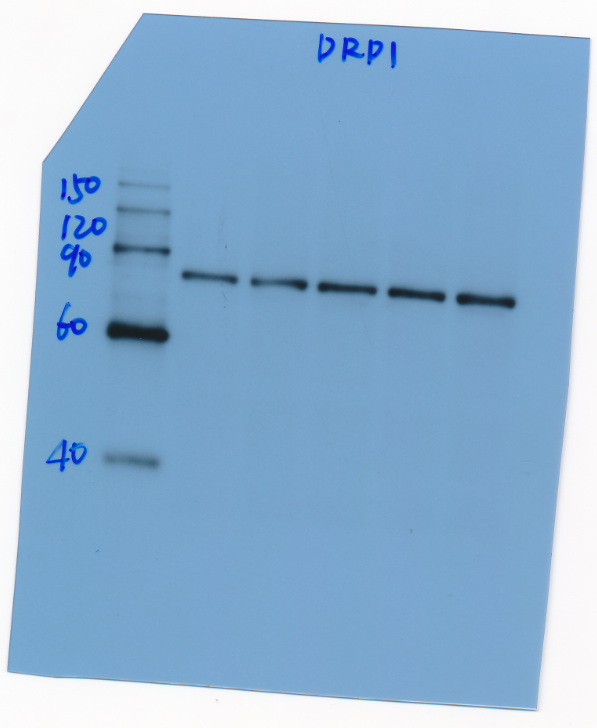


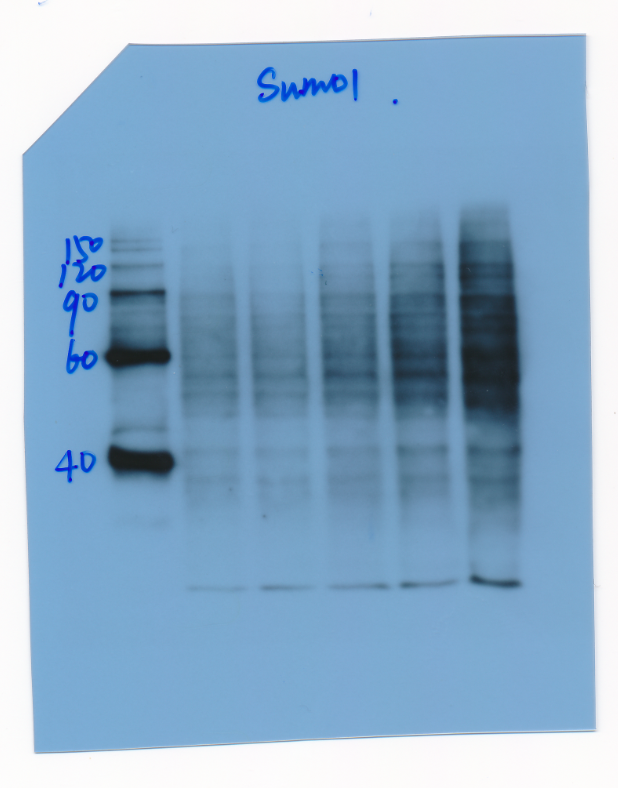

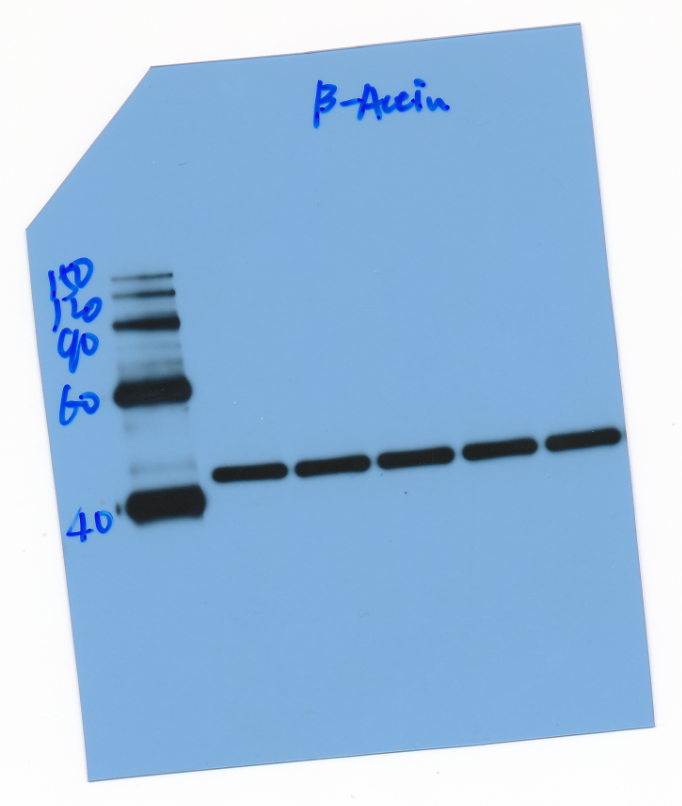


**Figure 3**


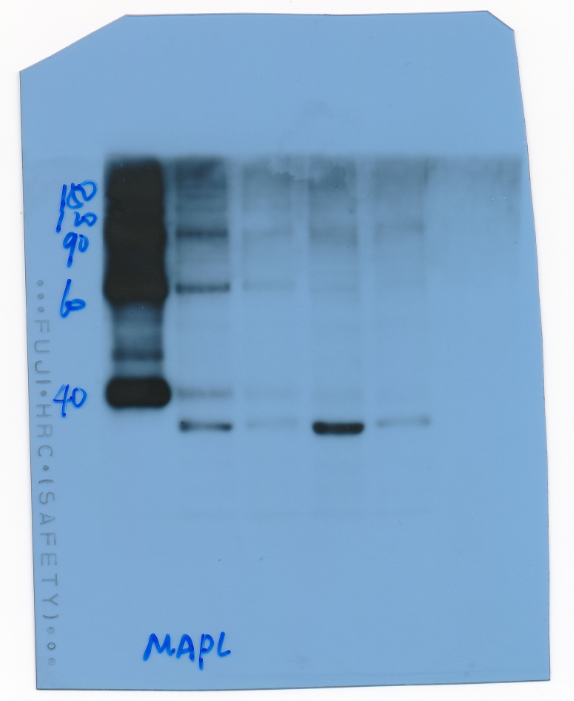

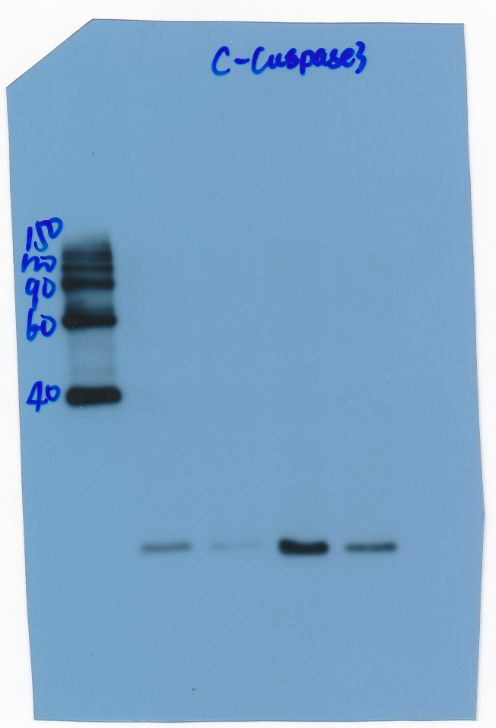

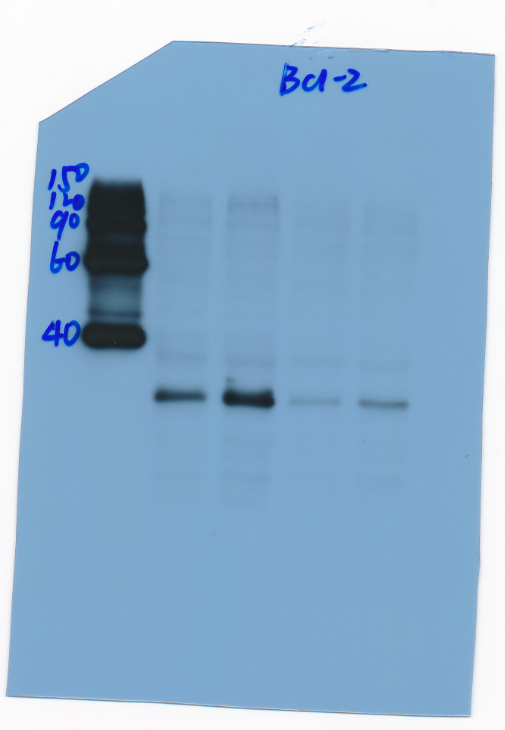

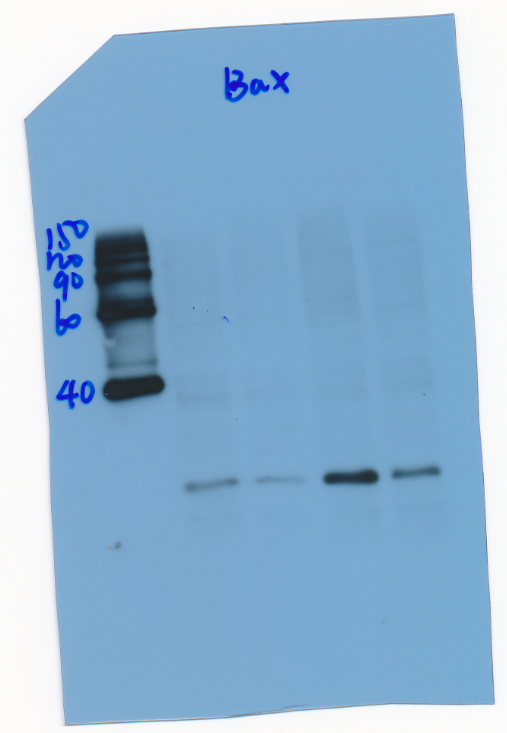

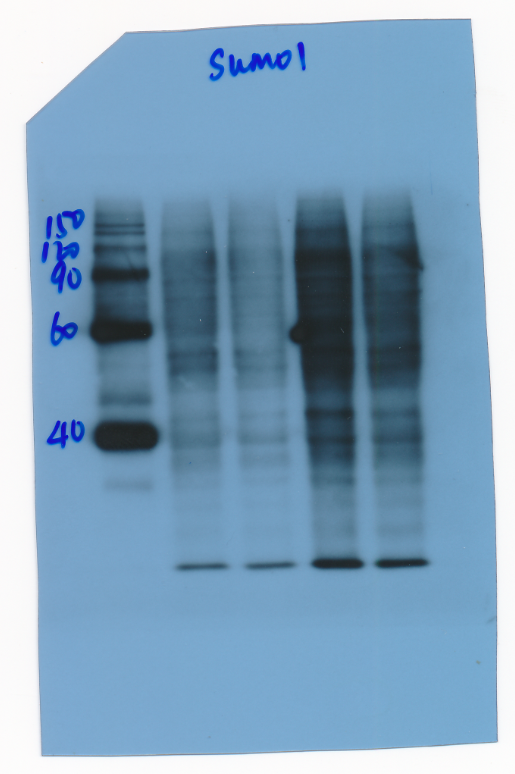

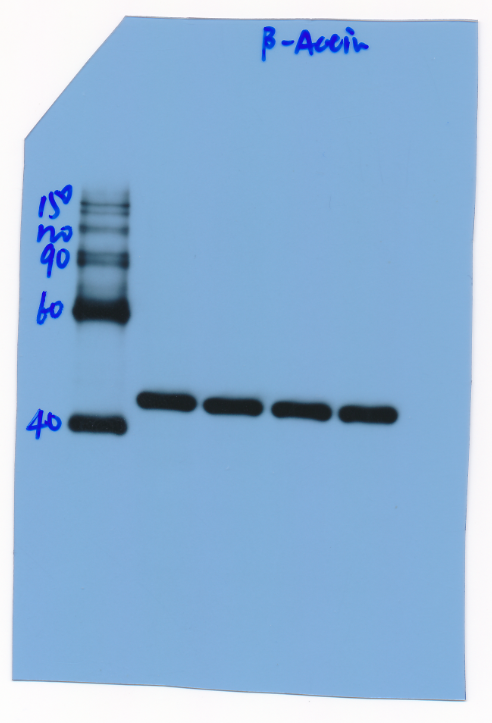


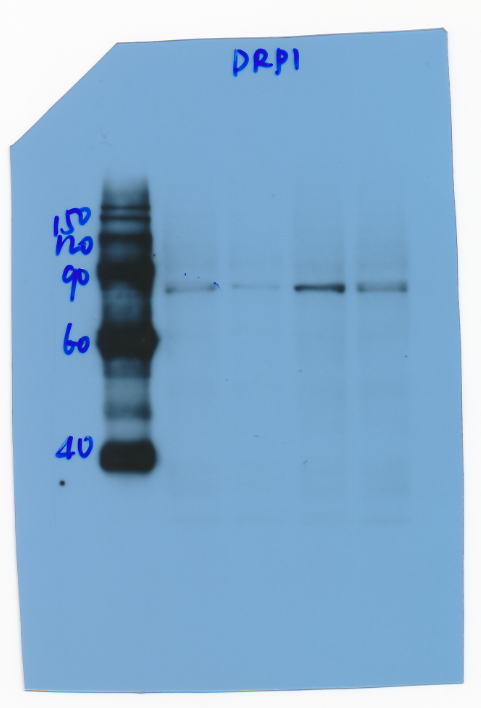

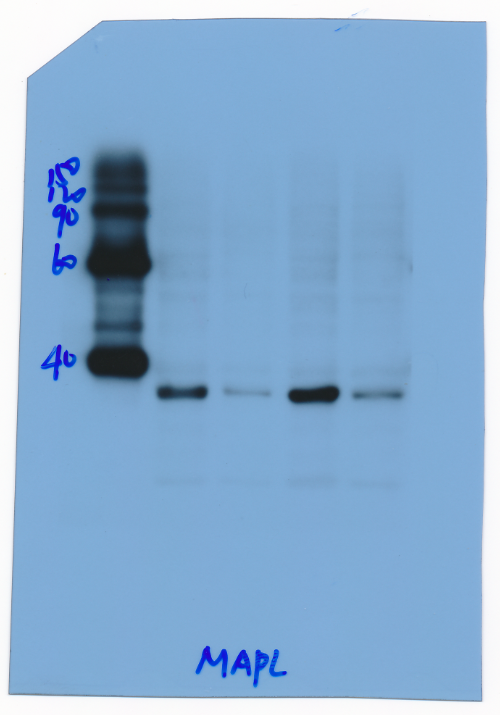

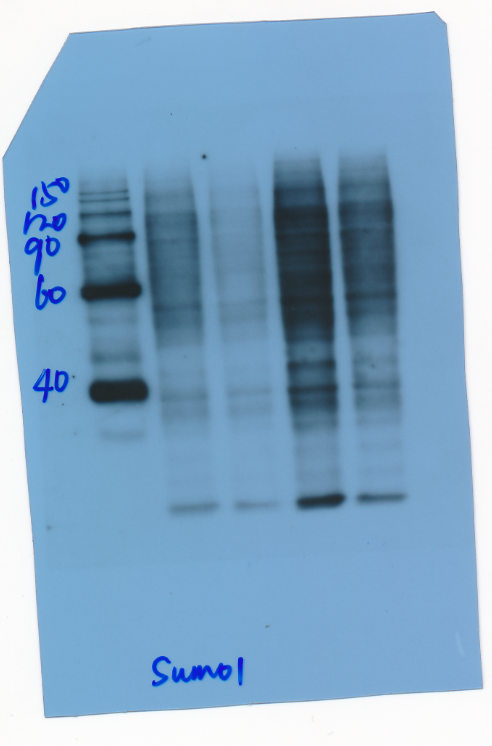

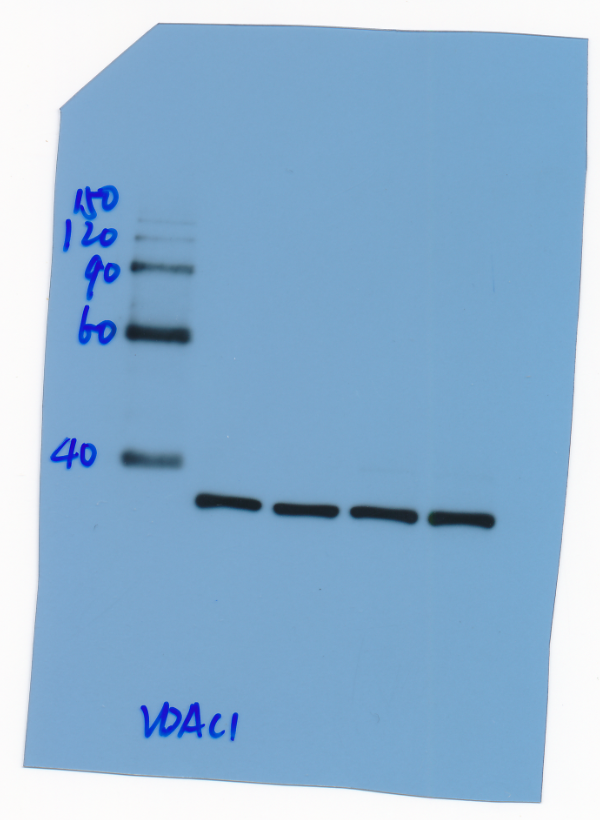


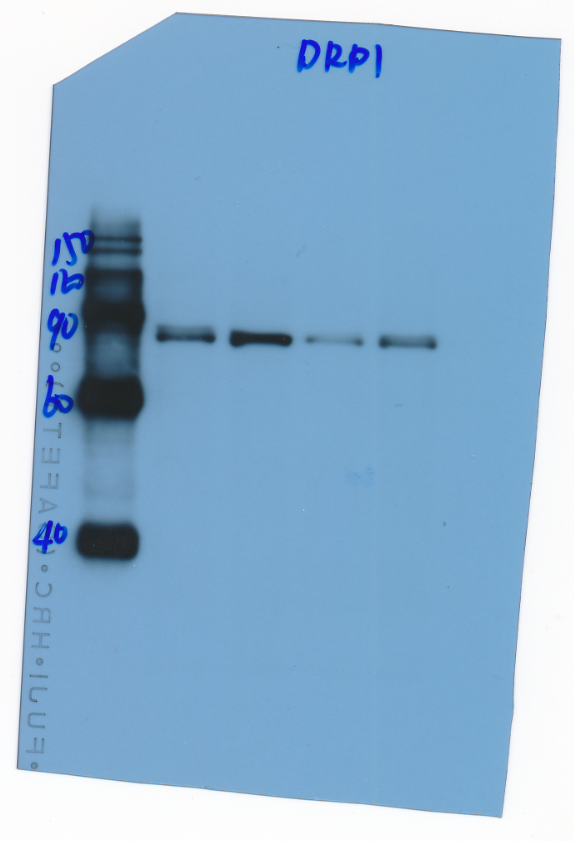

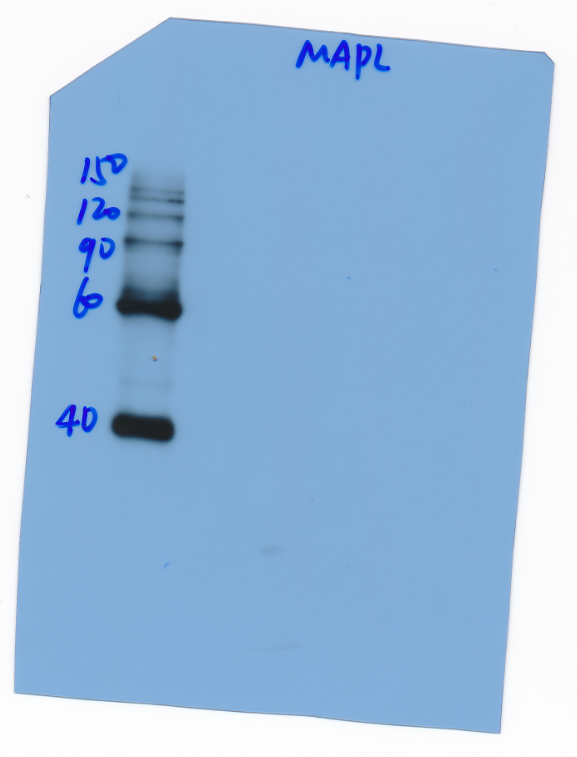

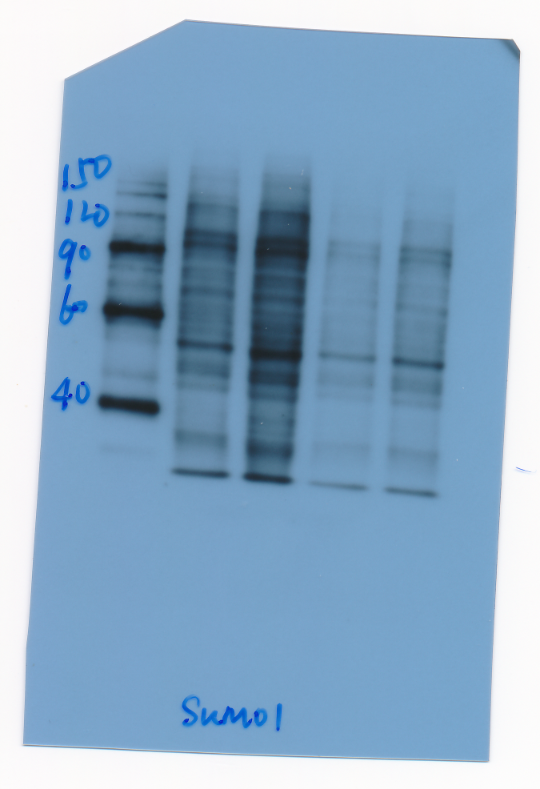

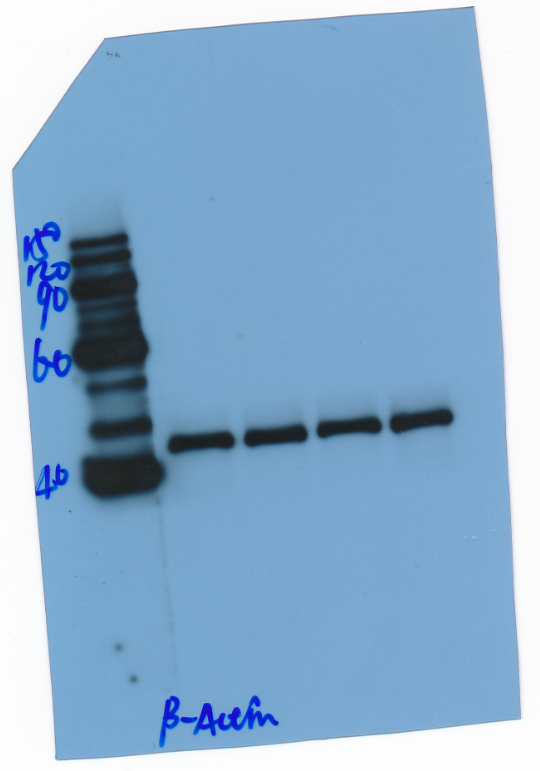


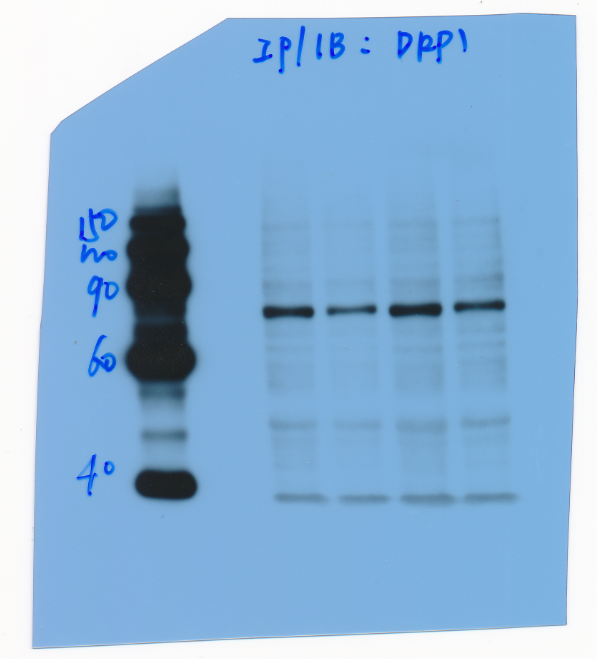

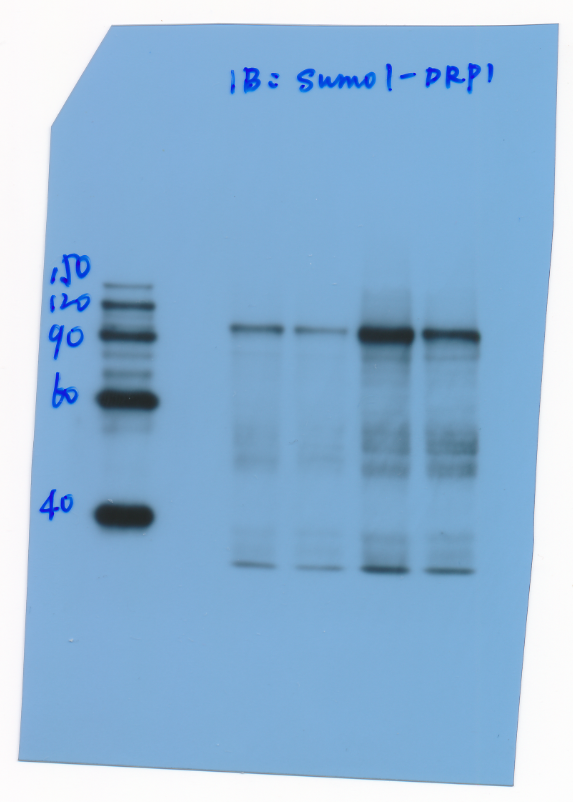

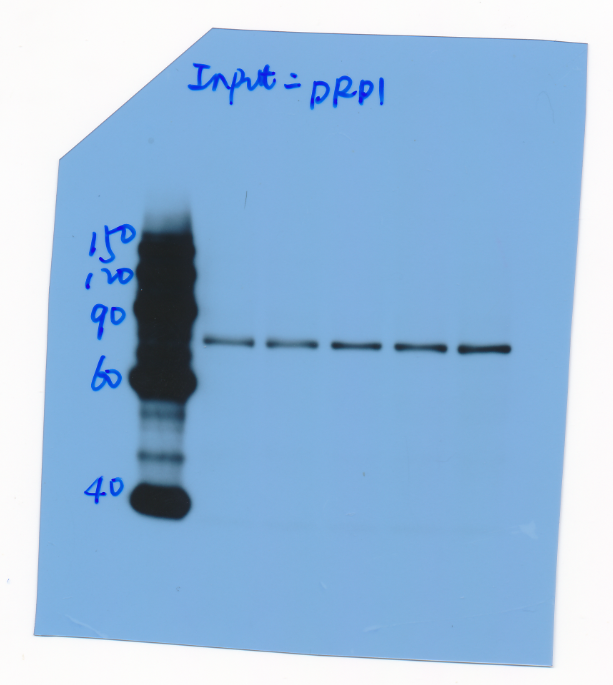


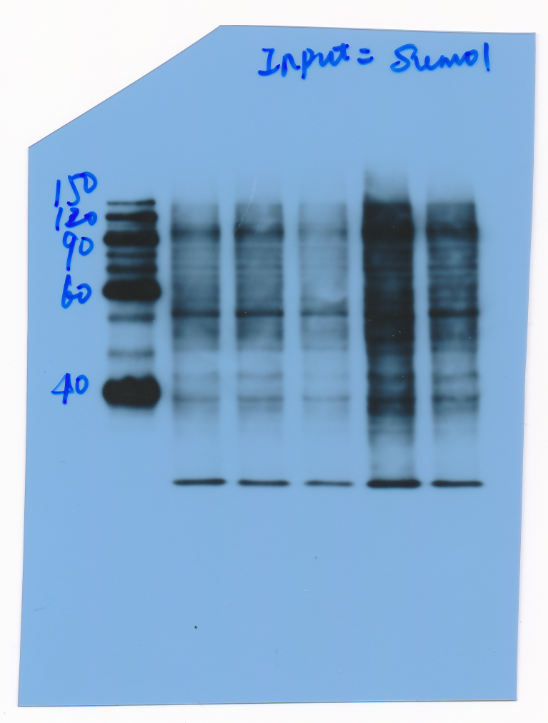

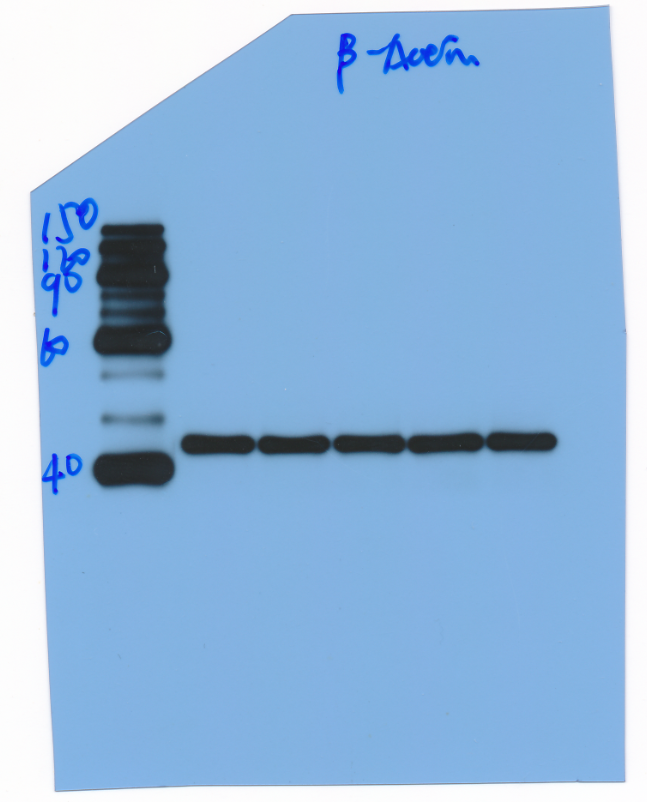


**Figure S7**

**
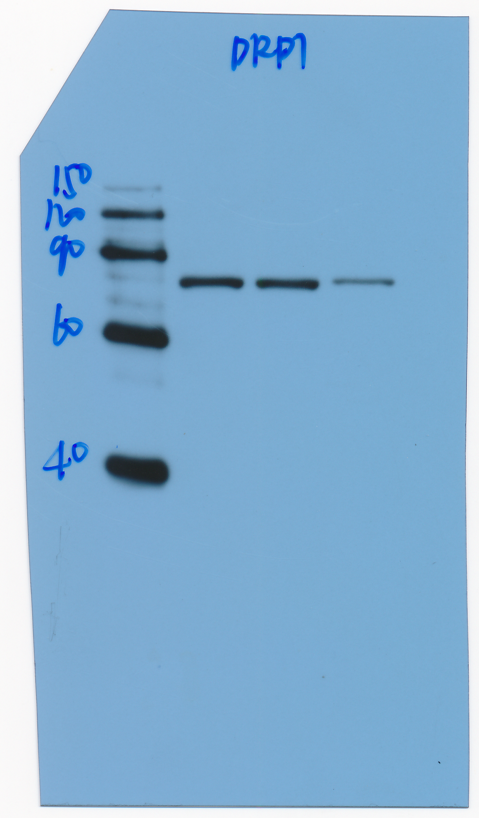
**
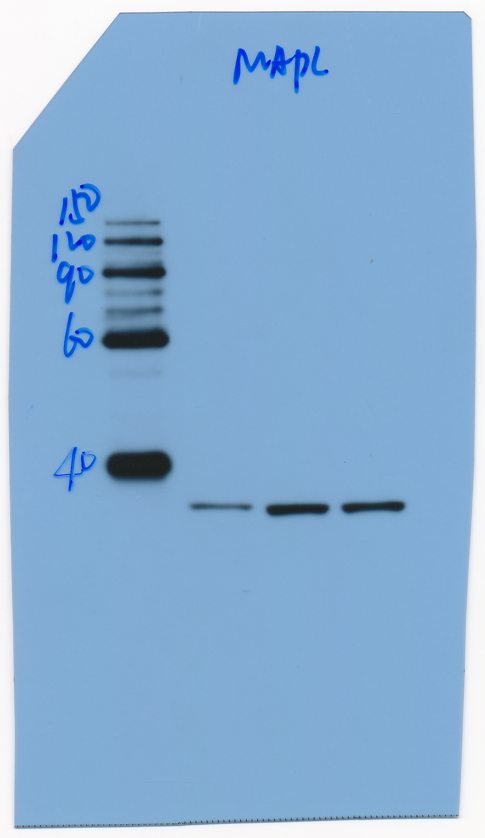

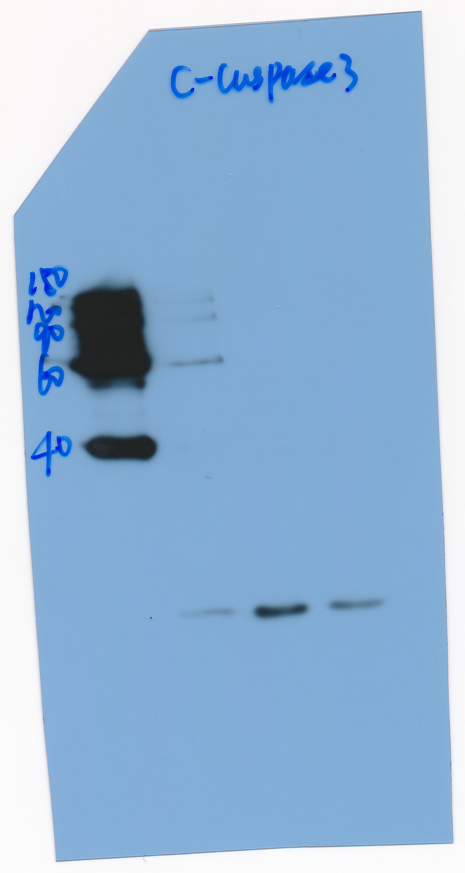


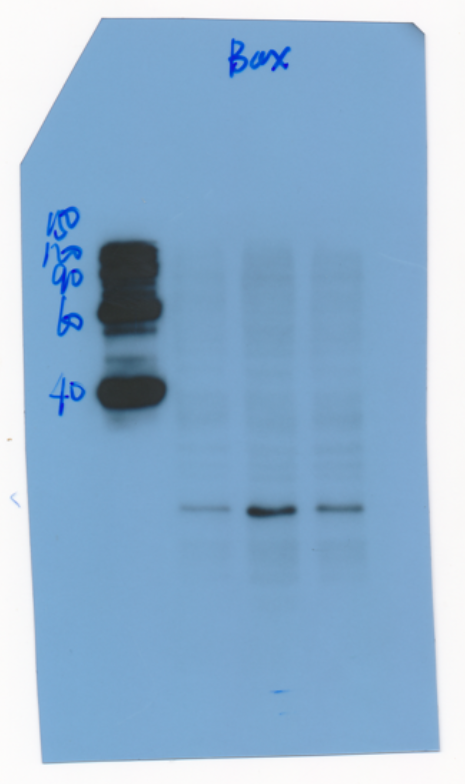

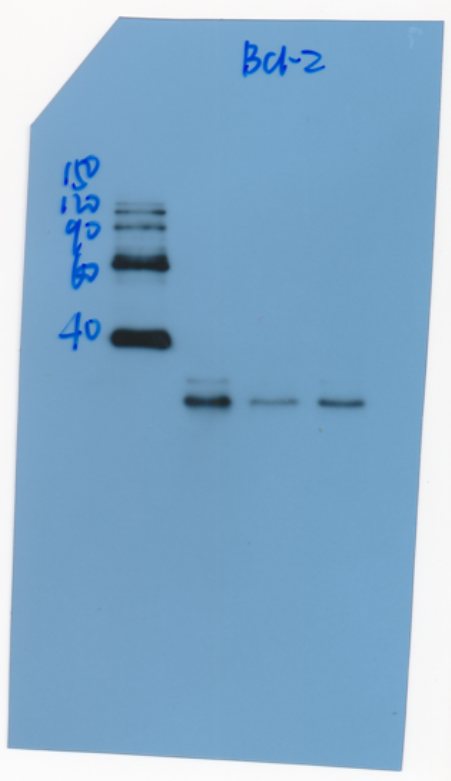

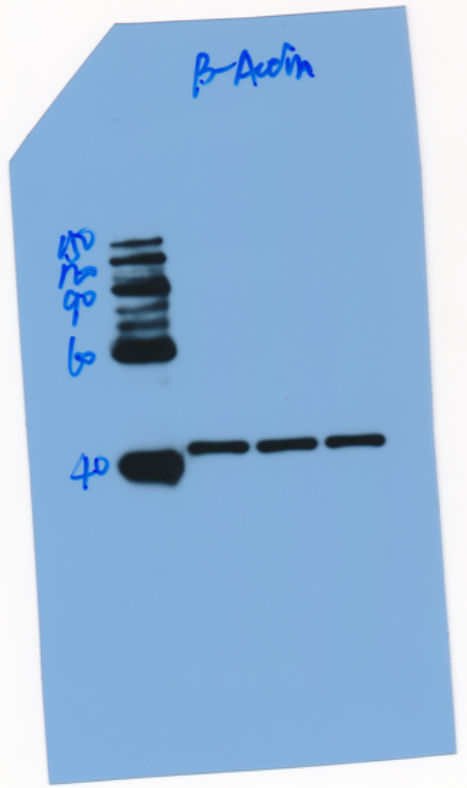


**Figure 4**


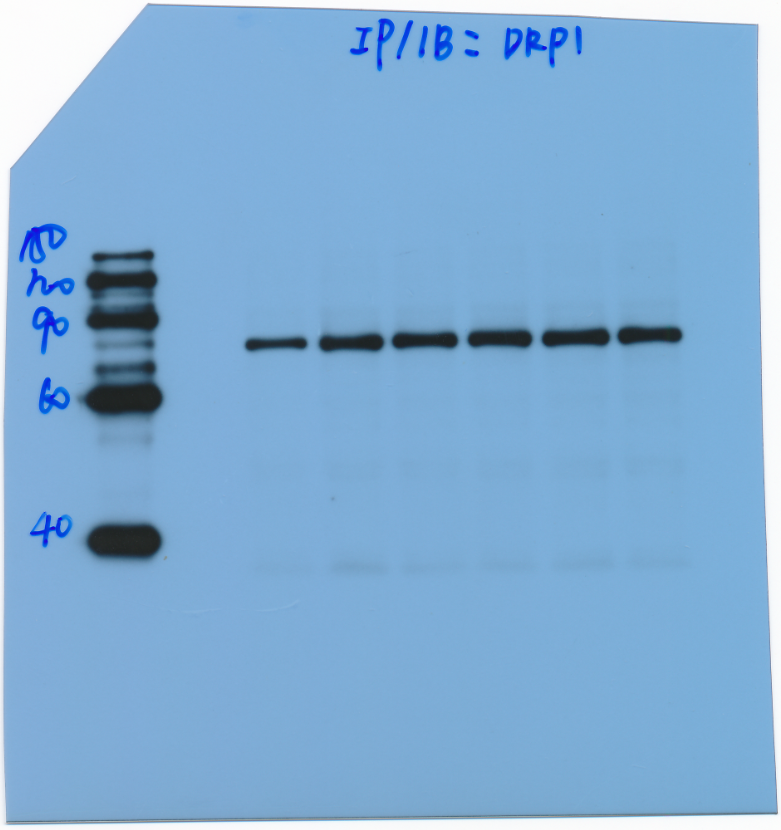

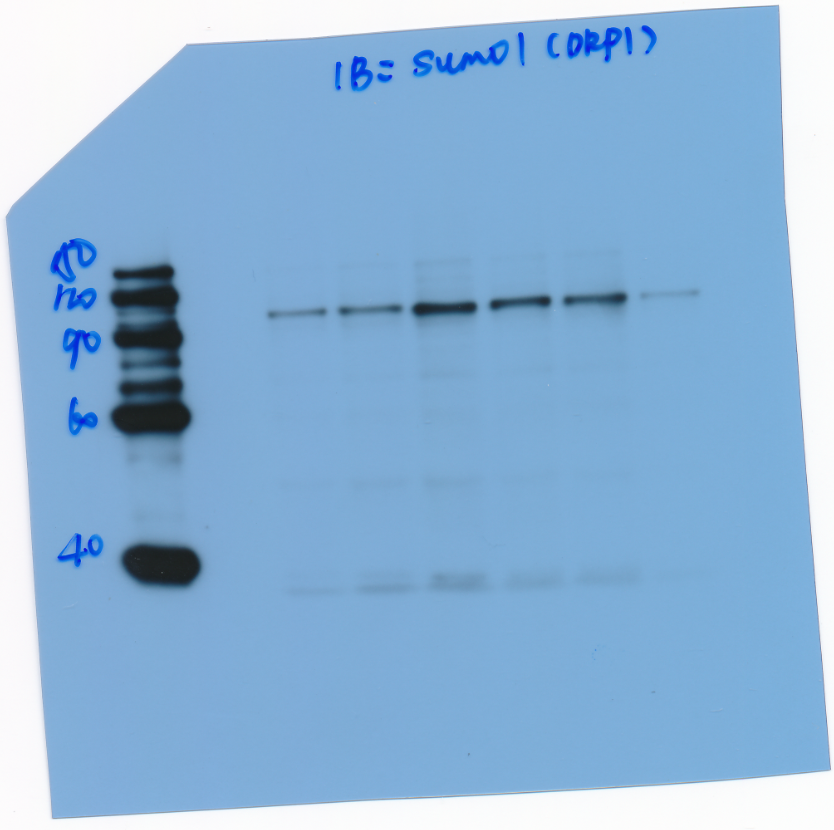

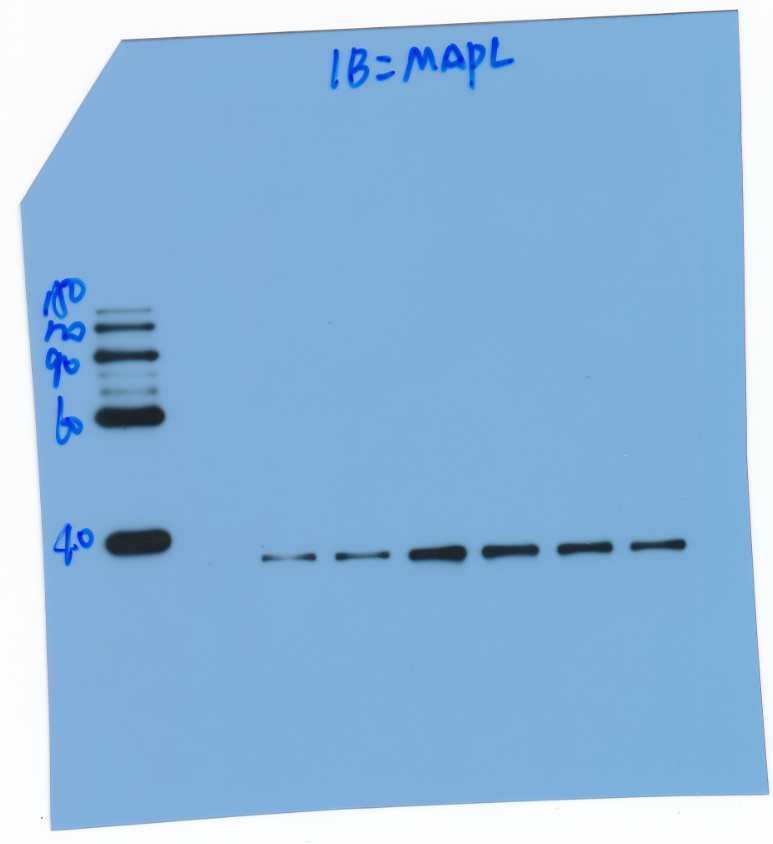


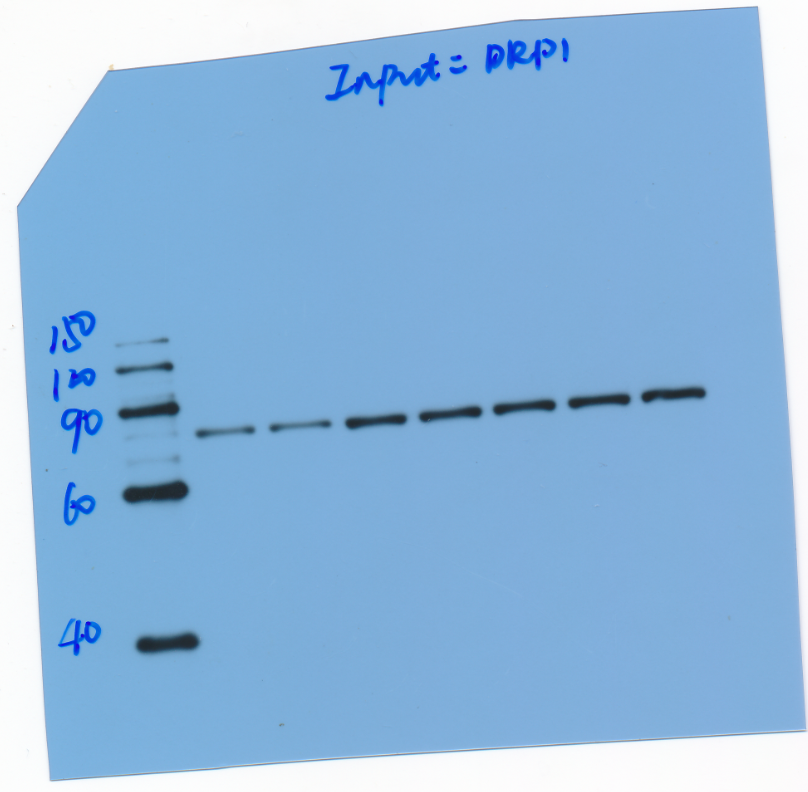

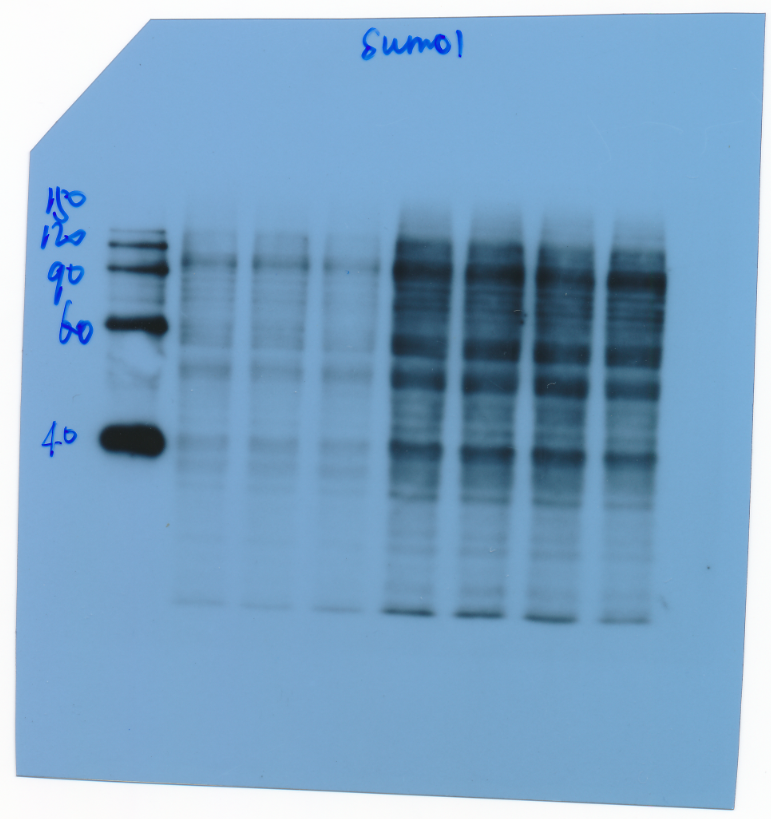

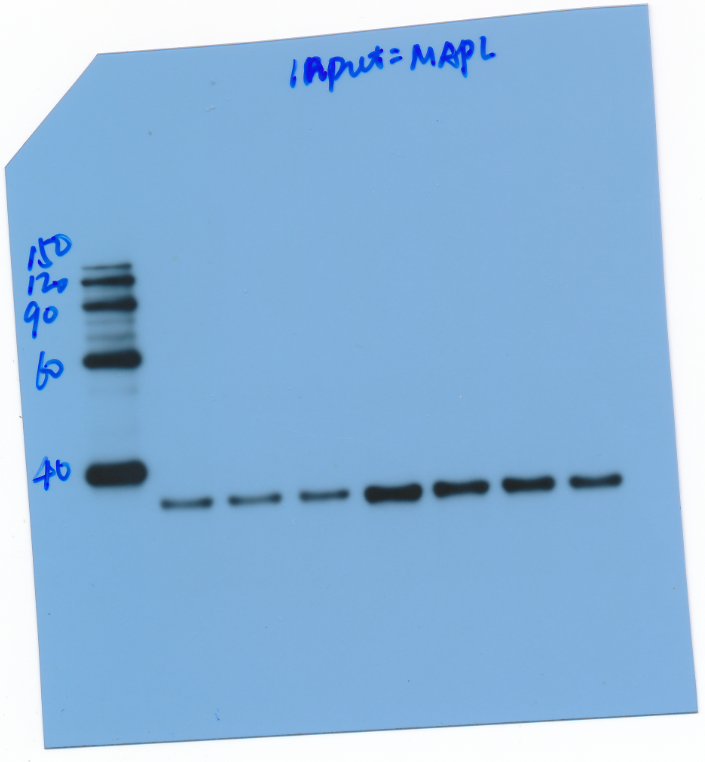


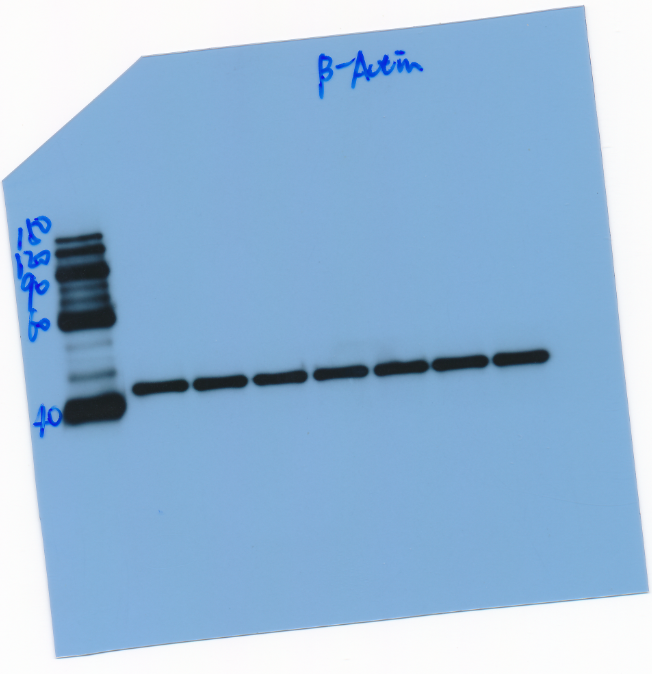


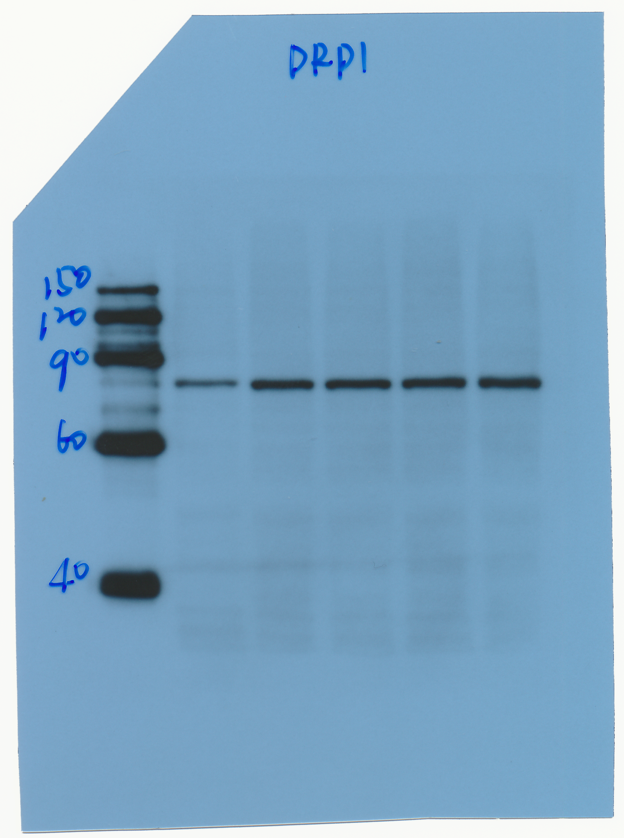

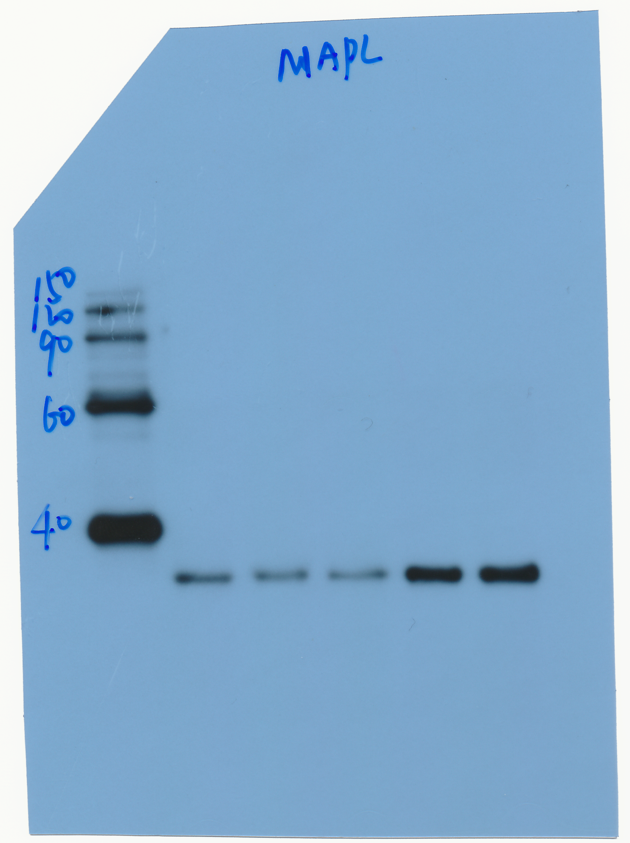

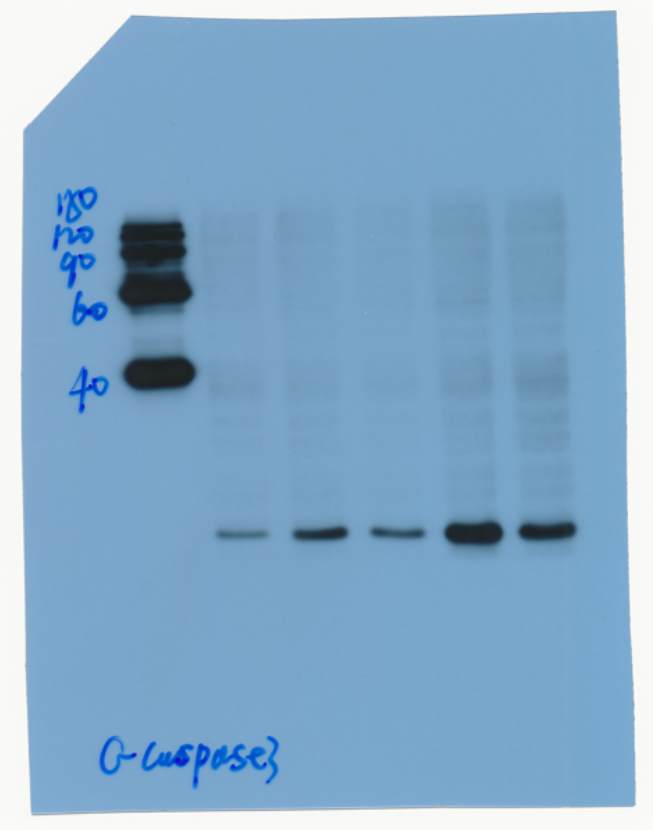


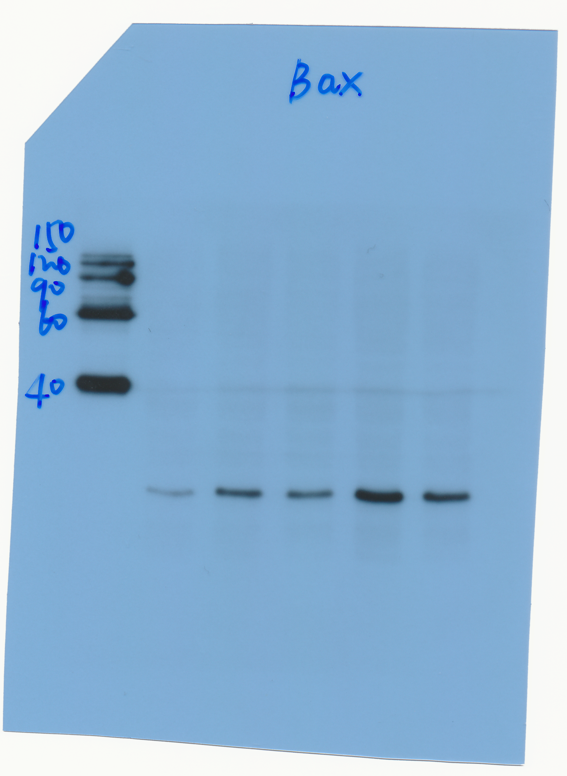

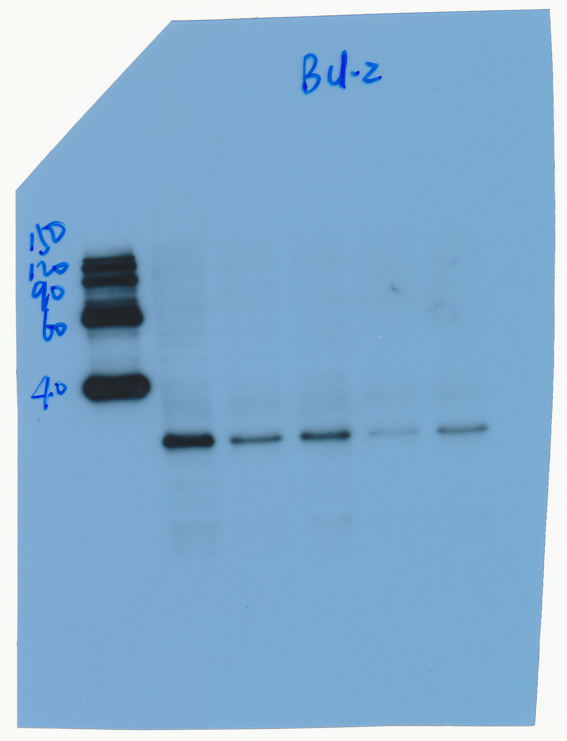

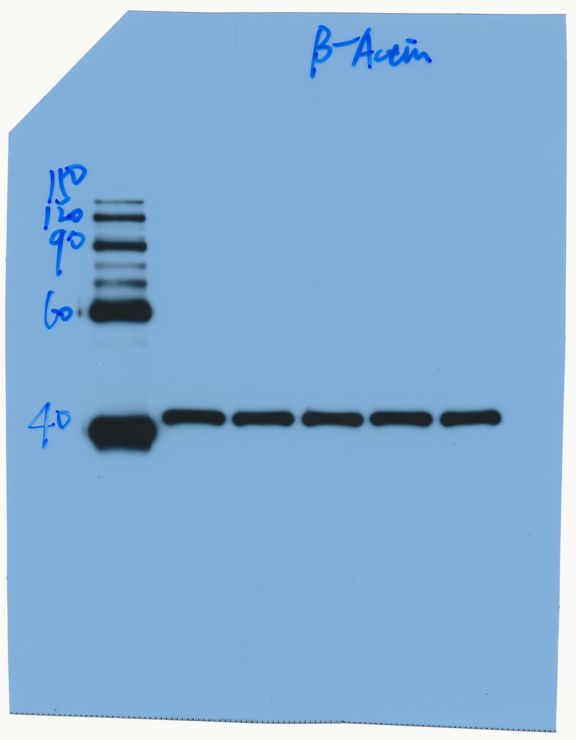


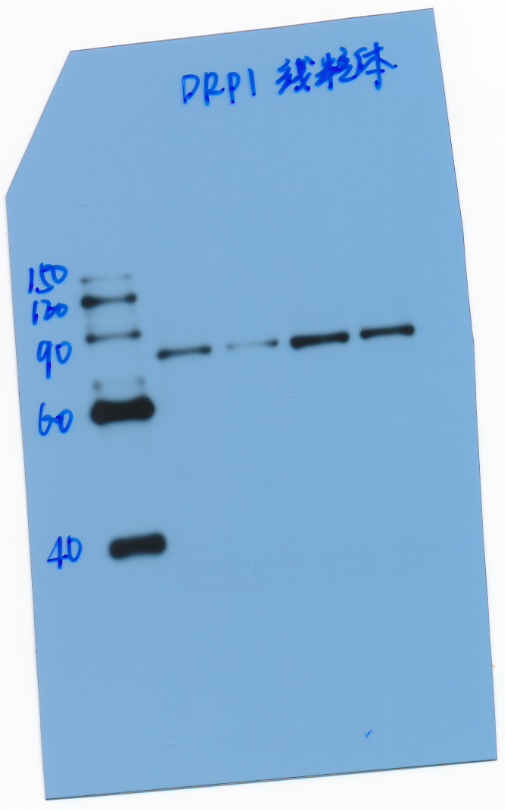

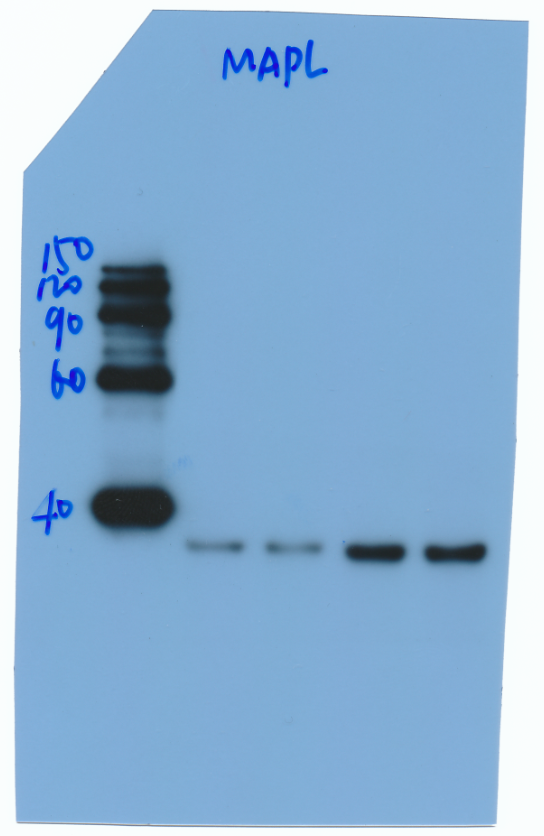

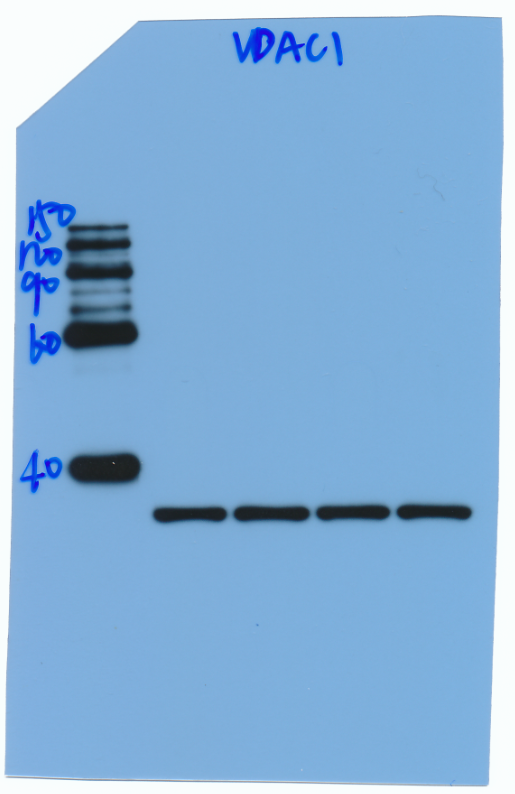


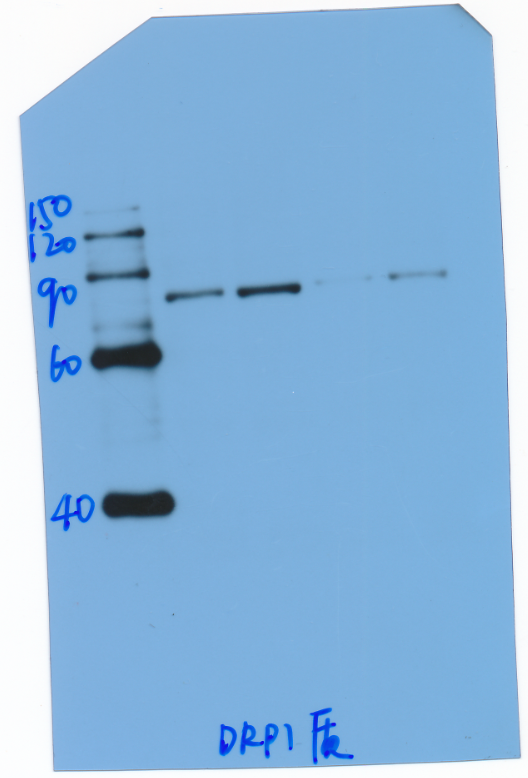

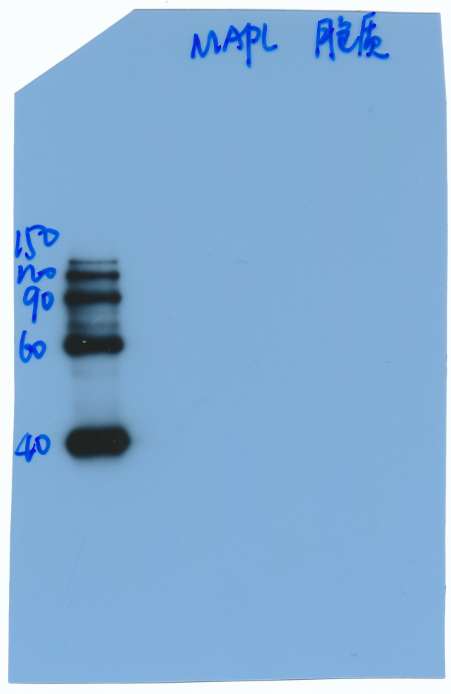

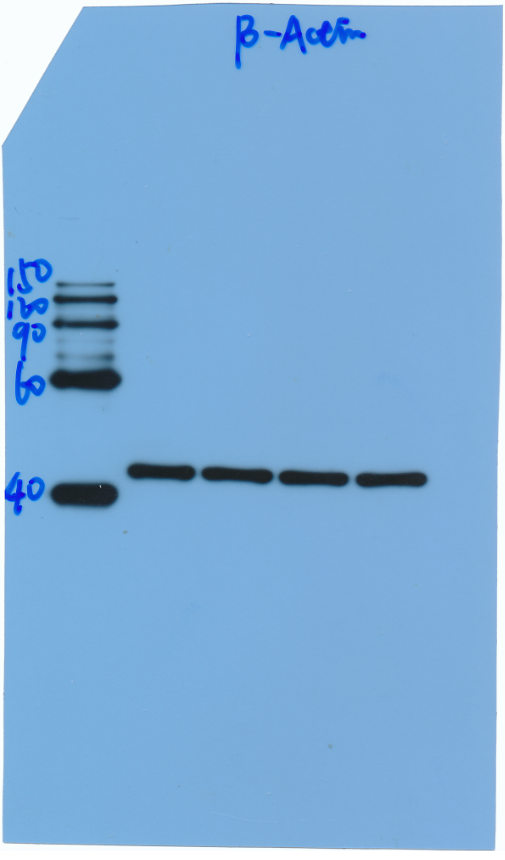


**Figure 5**


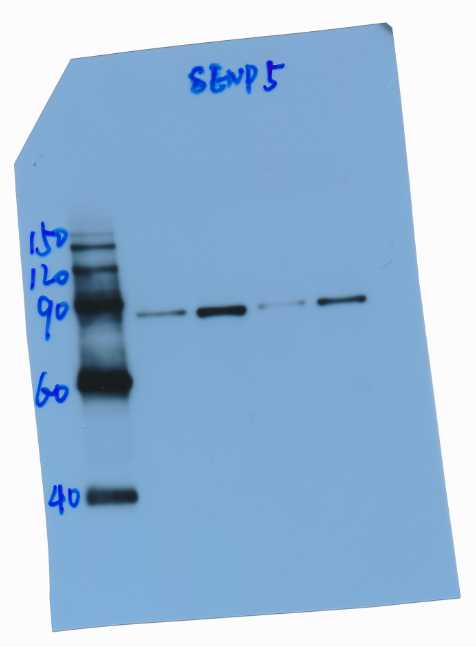

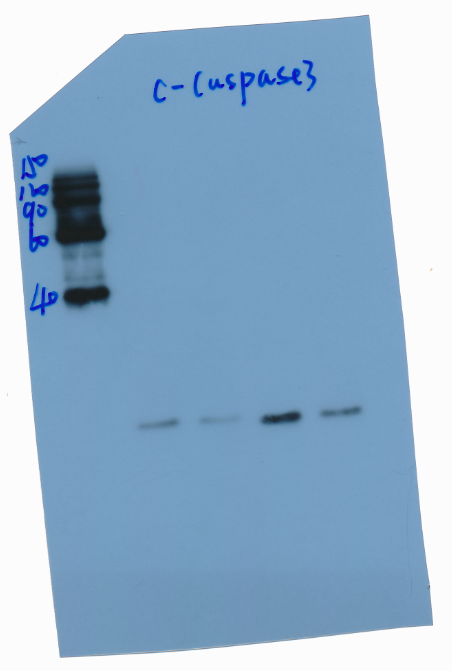

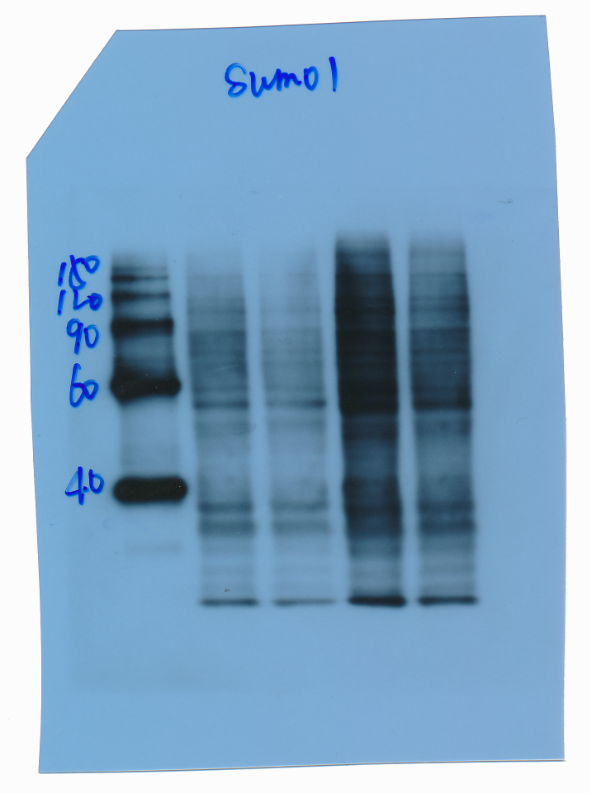

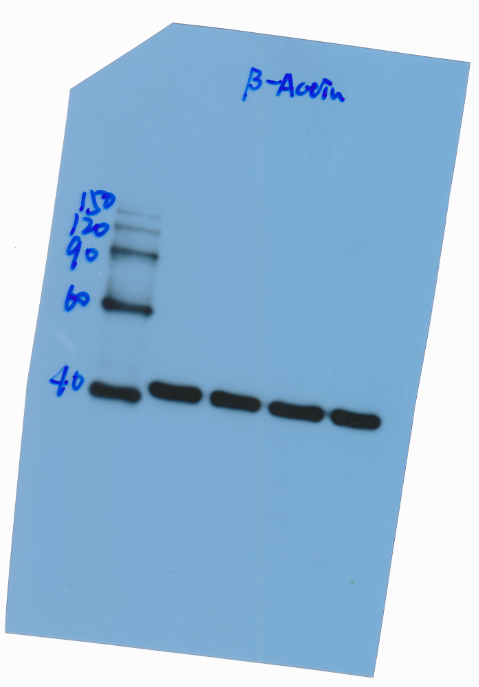


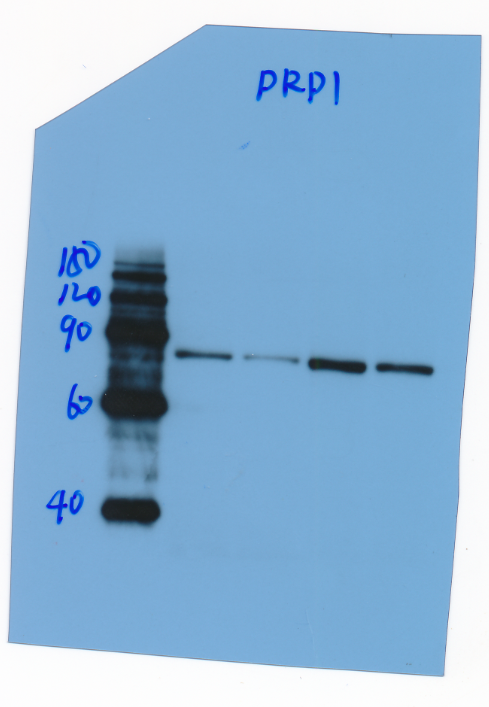

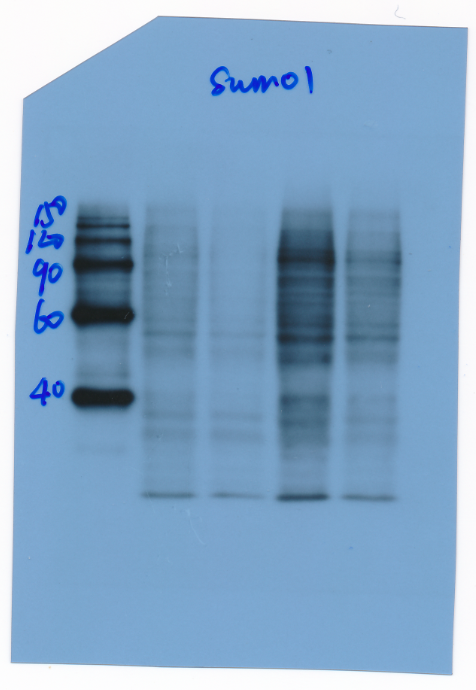

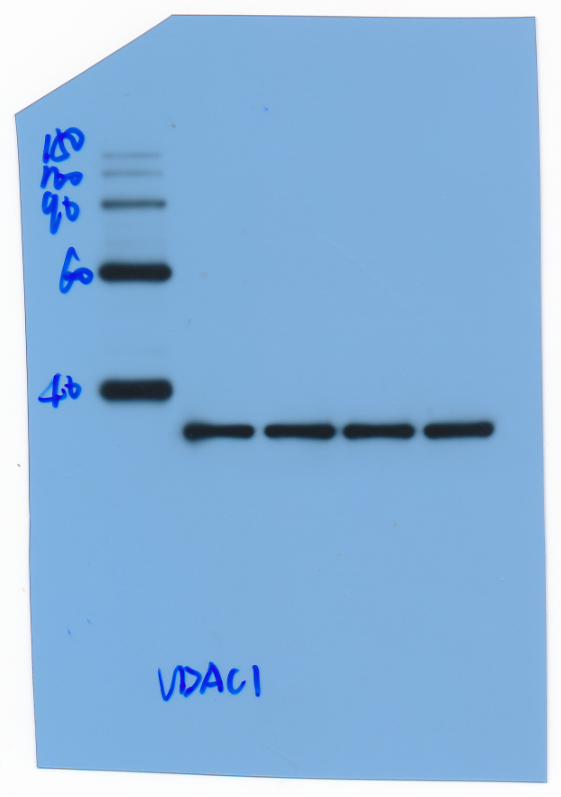


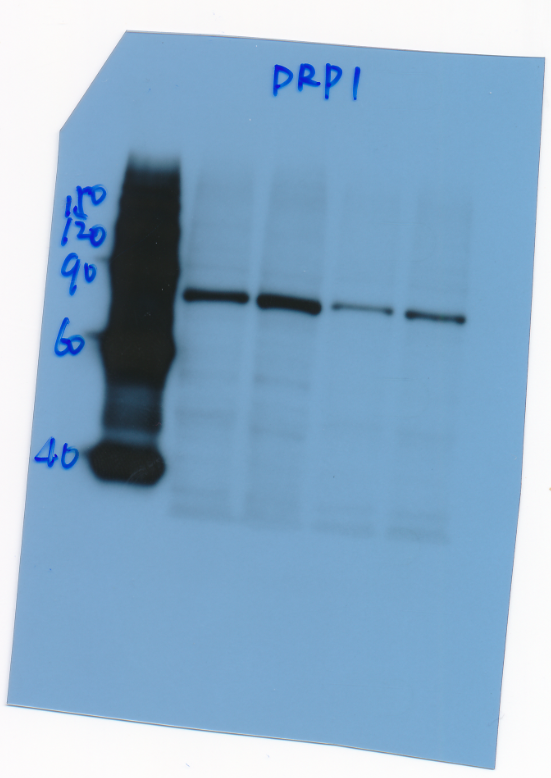

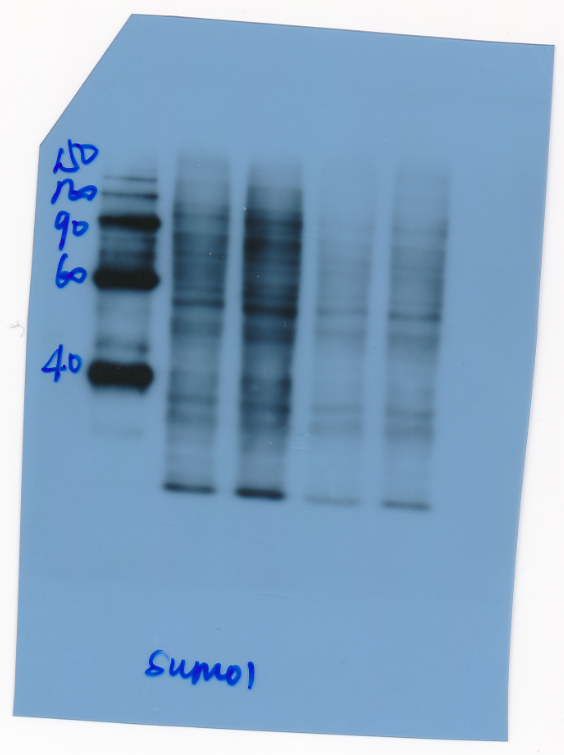

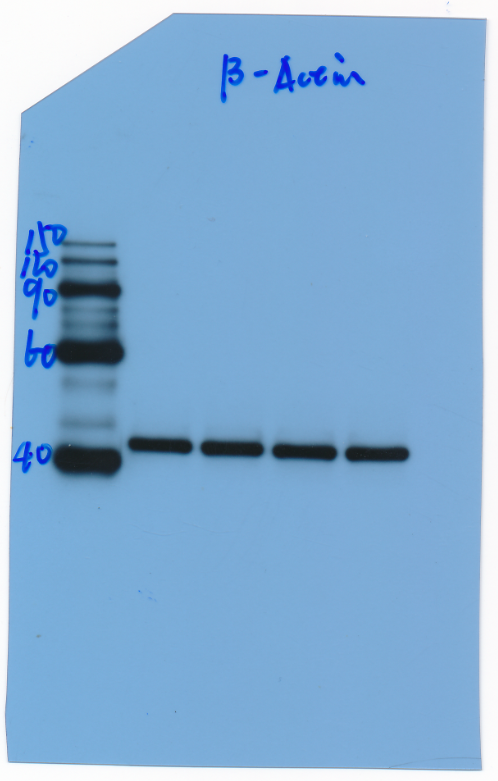


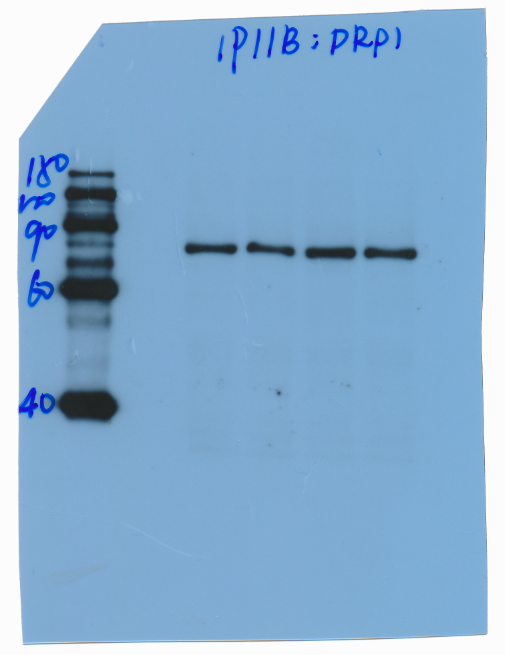

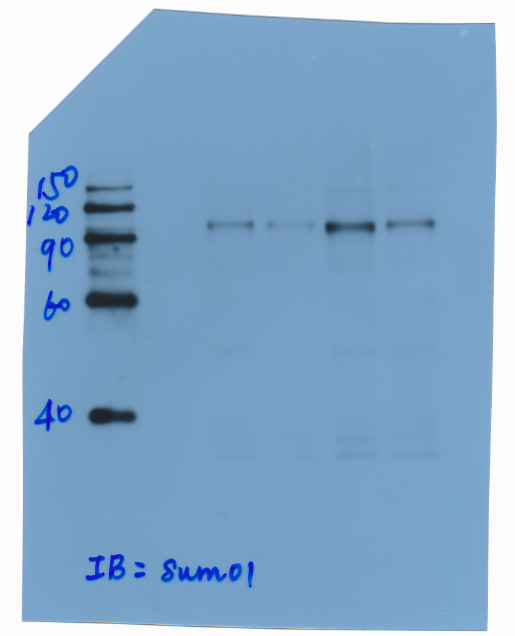

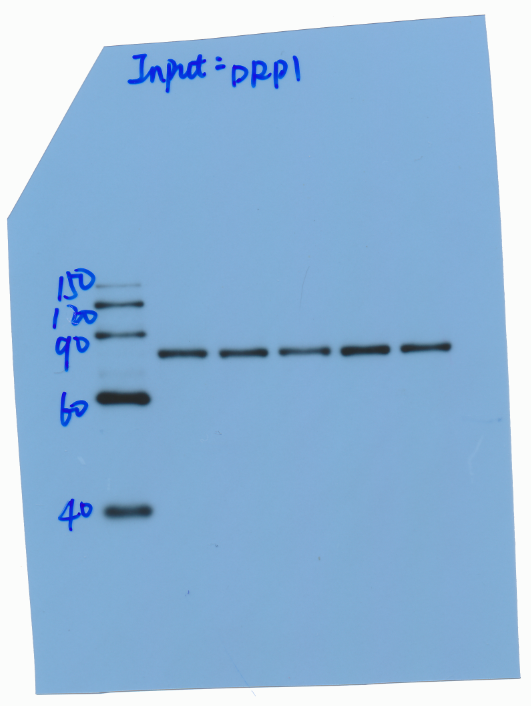

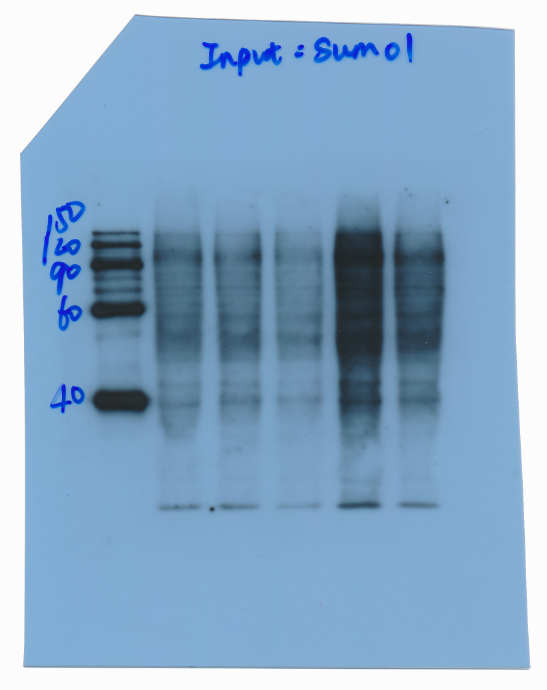

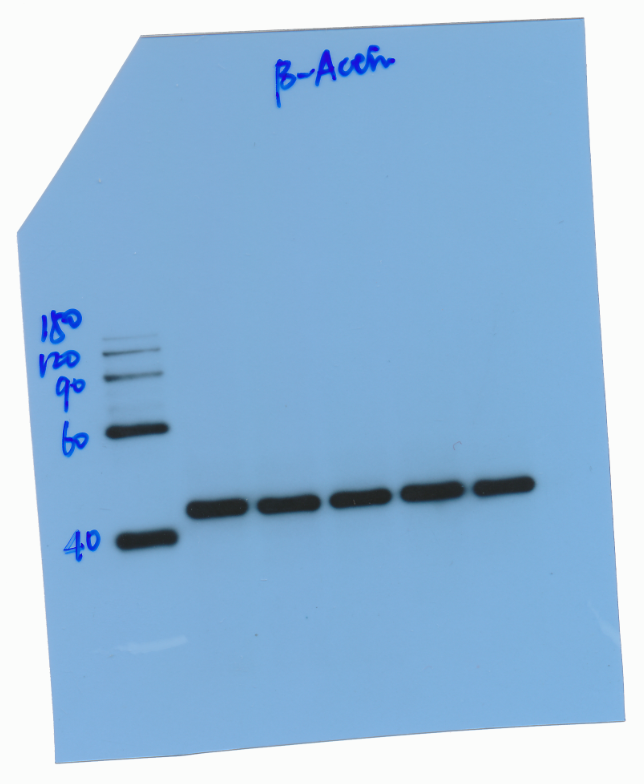


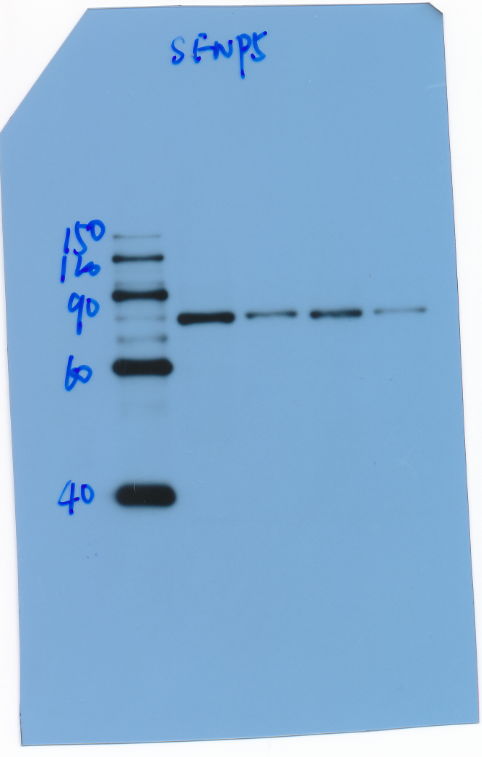

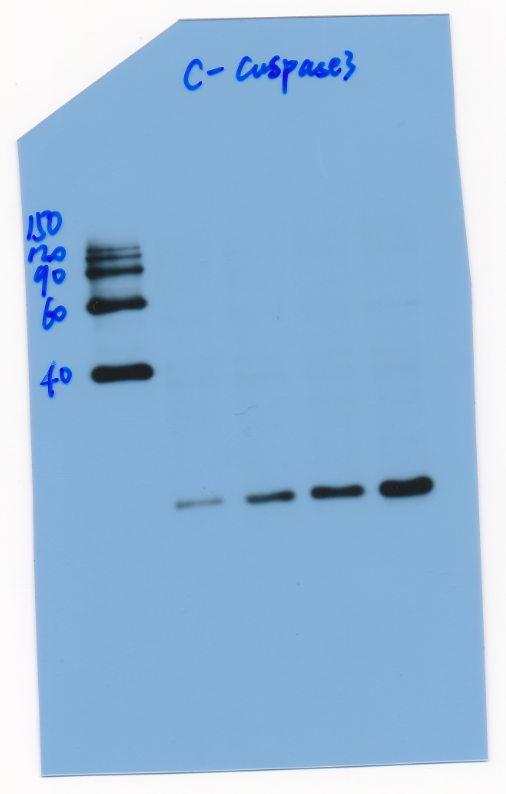

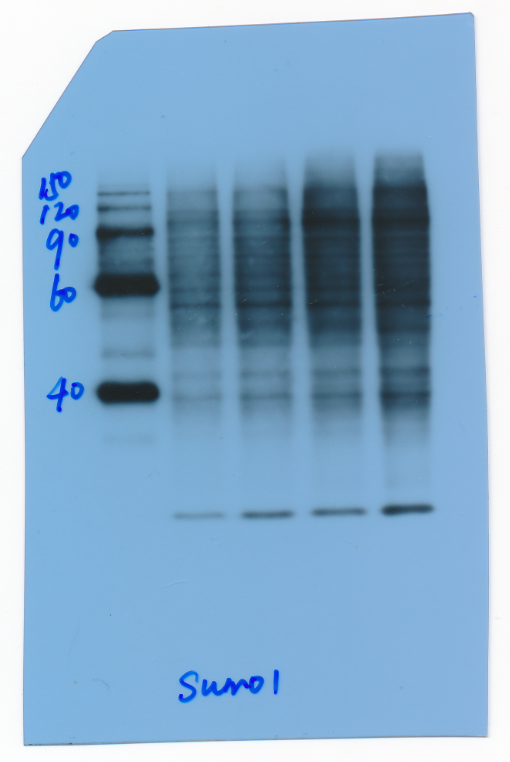

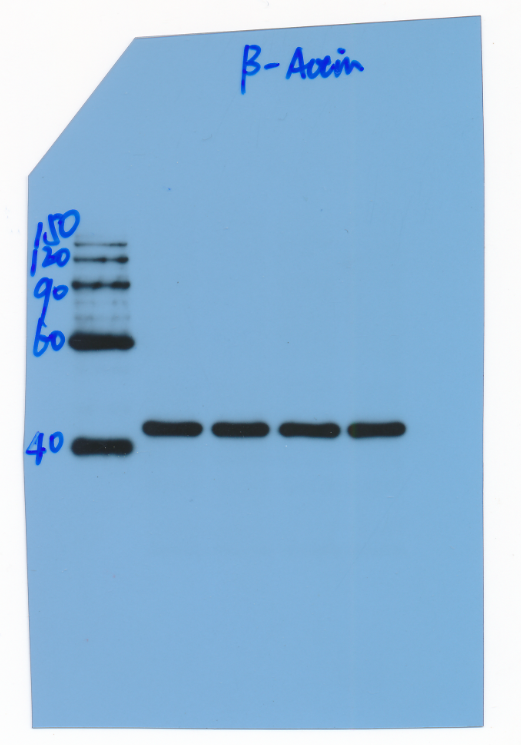


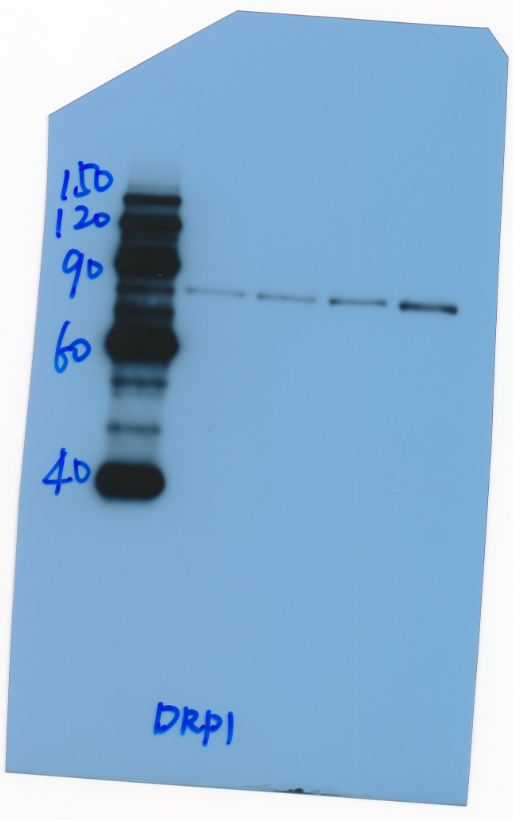

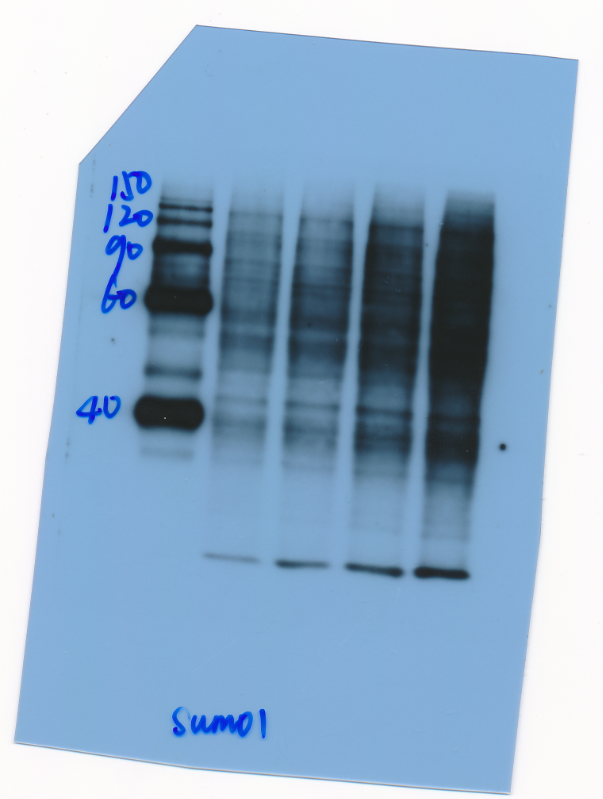

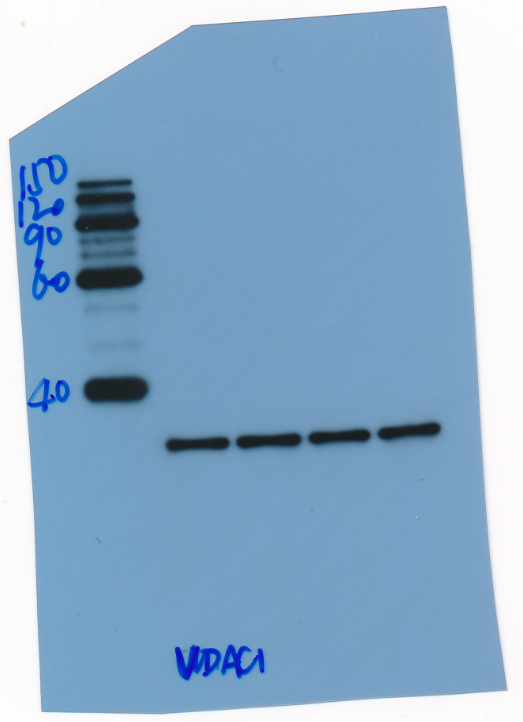


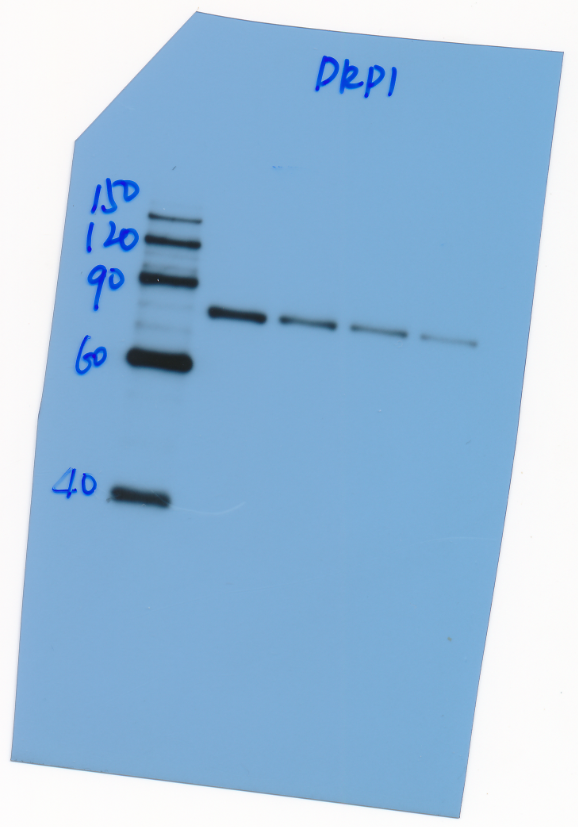

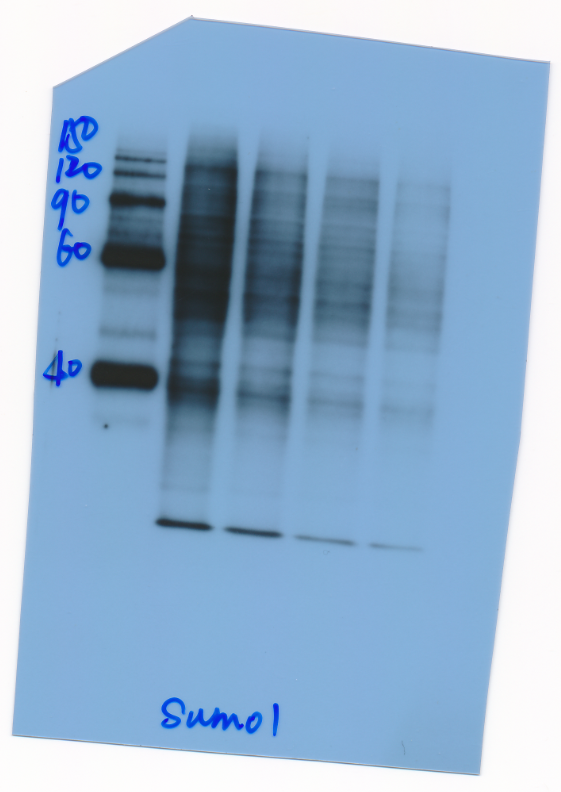


**Figure 6**

Supplement: Supplementary file 2 — Original Western Blots [file 41413_2025_449_MOESM2_ESM.docx]
